# Large-scale genomic and transcriptomic profiles of rice hybrids reveal a core mechanism underlying heterosis

**DOI:** 10.1186/s13059-022-02822-8

**Published:** 2022-12-22

**Authors:** Jianyin Xie, Weiping Wang, Tao Yang, Quan Zhang, Zhifang Zhang, Xiaoyang Zhu, Ni Li, Linran Zhi, Xiaoqian Ma, Shuyang Zhang, Yan Liu, Xueqiang Wang, Fengmei Li, Yan Zhao, Xuewei Jia, Jieyu Zhou, Ningjia Jiang, Gangling Li, Miaosong Liu, Shijin Liu, Lin Li, An Zeng, Mengke Du, Zhanying Zhang, Jinjie Li, Ziding Zhang, Zichao Li, Hongliang Zhang

**Affiliations:** 1 Key Laboratory of Crop Heterosis and Utilization, the Ministry of Education / Beijing Key Laboratory of Crop Genetic Improvement, China Agricultural University, Beijing, 100193 China; 2 State Key Laboratory of Hybrid Rice, Hunan Hybrid Rice Research Center, Changsha, 410125 China; 3 Sanya Nanfan Research Institute of Hainan University, Sanya, 572024 China; 4 Sanya Institute of China Agricultural University, Sanya, 572024 China; 5 State Key Laboratory for Agrobiotechnology, China Agricultural University, Beijing, 100193 China

**Keywords:** Hybrid rice, Heterosis, Nonadditive, HoIIB model

## Abstract

**Background:**

Heterosis is widely used in agriculture. However, its molecular mechanisms are still unclear in plants. Here, we develop, sequence, and record the phenotypes of 418 hybrids from crosses between two testers and 265 rice varieties from a mini-core collection.

**Results:**

Phenotypic analysis shows that heterosis is dependent on genetic backgrounds and environments. By genome-wide association study of 418 hybrids and their parents, we find that nonadditive QTLs are the main genetic contributors to heterosis. We show that nonadditive QTLs are more sensitive to the genetic background and environment than additive ones. Further simulations and experimental analysis support a novel mechanism, homo-insufficiency under insufficient background (HoIIB), underlying heterosis. We propose heterosis in most cases is not due to heterozygote advantage but homozygote disadvantage under the insufficient genetic background.

**Conclusion:**

The HoIIB model elucidates that genetic background insufficiency is the intrinsic mechanism of background dependence, and also the core mechanism of nonadditive effects and heterosis. This model can explain most known hypotheses and phenomena about heterosis, and thus provides a novel theory for hybrid rice breeding in future.

**Supplementary Information:**

The online version contains supplementary material available at 10.1186/s13059-022-02822-8.

## Background

Hybrid breeding is a revolutionary technology in agricultural production and for food security. Due to their dramatic increased yield by tens of percent and even double compared to inbreds [1], hybrids have been the important and even the main variety type for agricultural plants and animals. Rather different from traditional inbred breeding, which mainly exploit accumulation of homozygous beneficial alleles, hybrid breeding takes advantage of a phenomenon called heterosis or hybrid vigor, which shows that the hybrid from two genetically distantly related inbred lines show superior performance than their parents [2, 3].

Although heterosis has been utilized extensively in agriculture, its mechanistic understanding is still fragmentary and challenging [4]. Regarding its genetic basis, there are three classical hypothesis, including dominance [5], overdominance [6, 7], and epistasis [8, 9]. Through quantitative trait loci (QTL) mapping and genome-wide association studies (GWAS), previous study of some crops has identified a large number of genetic variants with various types of genetic effects [10–13]. Meanwhile, several well designed studies at the transcriptome level have been carried out in plant hybrids such as *Arabidopsis*, maize, and rice, and many genes appear to be dominant, overdominant, or parent-specific in expression [14–16]. At the single gene level, genes with partial or complete dominance effect are commonly observed in many species, such as *PMA1* and *MSB2* in yeast [17], *PCSK9* in human heart diseases [18], *Dw3* in sorghum, and *GS3* and *Ghd7* in rice [19, 20]. There are also several cases that one gene displays overdominance effect, such as the *SFT* gene affecting fruit yield of tomato [21], the SHELL gene controlling the oil yield in oil palm [22], and the *FNS* gene impacting flower color in *Mimulus lewisii* [23]. The second typical view on heterosis suggested that the pleiotropic functions of one gene or factor with compromise and balance [24, 25], or the cumulation or interaction between or among multi-factors, such as at the levels of alleles, genes, traits, and so on [26–28], represent the important genetic mechanism underlying heterosis. The third explanation is that hierarchical effects at different levels or aspects contribute to heterosis, such as the multiplicative effect on yield by its component traits, where accumulation of partial dominance usually occurs [29]. However, the theories mentioned above are challenged to address such a question: how does a single gene function as nonadditive effect at the molecular level, and is there a core mechanism under it?

Rice is one of the crops that successfully utilize heterosis in breeding. Numerous studies have been carried out to investigate genetic and molecular mechanisms of heterosis in rice; however, there is still no consensus on such mechanisms [30–32]. Early QTL analysis in rice hybrid suggested that dominance accumulation, overdominance, and epistasis all contributed much to heterosis [10, 30]. The subsequent research based on an immortalized F_2_ population from an *indica-indica* rice hybrid indicated that the contributions of the dominant factors varied by traits and single-locus dominance has relatively small contributions in all traits [31]. Recent studies using 1495 commercial hybrids and 10,074 F_2_ individuals from 17 crosses demonstrated that the heterosis mainly attributes to accumulation of numerous rare superior alleles with positive dominance [32, 33]. Although these researches have made great progress, two issues still need to be addressed in these studies. First, most of these studies mainly focused on commercial hybrids or their derived populations [32], such as the “immortalized F_2_” derived from Zhenshan97 and Minghui63 [31], and the BCF_1_ population derived from Peiai64S and 9311 [34]. Further extensive studies using combinations derived from a wider spectrum of rice germplasm resources may provide more common or general mechanistic understanding of the heterosis. Second, most of them mainly focused on the proportions and contributions of various genetic component (including dominance and overdominance) based on three traditional hypotheses to heterosis. The more challenged and interested question is what is the intrinsic mechanism of additive and nonadditive effects underlying heterosis.

To get insight into rice heterosis, we generate and record the phenotypes of 418 hybrids from crosses between two testers (*japonica* variety Nipponbare and *indica* variety 9311) and 265 rice varieties from a mini-core collection [35] (Additional file 1: Figure S1 and Additional file 2: Table S1). The transcriptomes of Liangyoupei 9 hybrid from 1 to 3 mm young panicle [34], transcriptomes of 4 Arabidopsis combinations [14, 36], phenotypic data of 1404×2 and 265×1 maize hybrids [37, 38], 120×15 wheat hybrid [39], and phenotypic data of 5918 yeast mutants were collected and used [40]. The analysis of these large-scale hybrid phenomes, genomes, and transcriptomes indicated that it is a common phenomenon that the hybrids biased from the mean performance of their parents at phenotype of the traits, genetic effect of QTLs, or transcription level of genes, which we call them nonadditive phenomena here; and it was found that these nonadditive phenomena exhibit more dependence on the background than additive phenomena. Simulation and a series of evidences from phenotypic, QTL, transcription, and yeast experiments demonstrated that background insufficiency is a core mechanism of heterosis (Additional file 1: Figure S2). Therefore, we proposed a novel model that shows the dependence of heterosis on background, i.e., homo-insufficiency under insufficient background (HoIIB). As indicated by HoIIB, it is the genetic background insufficient to maximize the function of two homo-alleles in parents, but relatively or even completely sufficient to maximize the function of one allele in F_1_, thus resulting in the insufficient function of two homo-alleles in parents, but the relatively or completely sufficient function of one allele in F_1_, that renders the target locus nonadditive in effect, as contributing to heterosis. So heterosis in most cases is not the heterozygote advantage but the homozygote disadvantage (insufficiency in function and performance) under the insufficient genetic background. The model can explain the most known hypotheses and phenomena about heterosis, thus providing a novel theory for future hybrid rice breeding.

## Results

### Heterosis is found to depend on environment and genetic background

We identified 4,625,141 SNPs in the parent panel (*N* = 267), after excluding SNPs with minor allele frequency (MAF) less than 5% and missing rate larger than 50%. According the neighbor-joining tree of 267 parental lines based on the above SNPs and the posterior validation errors in different number of run K in admixture, all the 267 lines could be classified into *japonica* and *indica* subspecies (Additional file 1: Figure S1). Thus, our obtained 418 hybrids include two kinds of intra-subspecific combinations, i.e., *japonica*×Nippponbare (J×Nip) and *indica*×9311 (I×9311), and two kinds of inter-subspecific combinations, i.e., *japonica*×9311 (J×9311) and *indica*×Nipponbare (I×Nip).

We phenotyped the 418 hybrids and their 267 parents in 2013 at Changsha (CS) (28° 13′ N, 112° 58′ E, a long-day environment) and Sanya (SY) (18° 10′ N, 109° 28′ E, a short-day environment) of China. Six yield-related traits were investigated, including spikelet number per panicle (SPP) and its two component traits (both primary and secondary branch numbers per panicle (PBP and SBP)), 1000-grain weight (KGW), panicle number per plant (PNP), and grain weight per plant (GWP) (Additional file 2: Table S2).

**Fig. 1:**
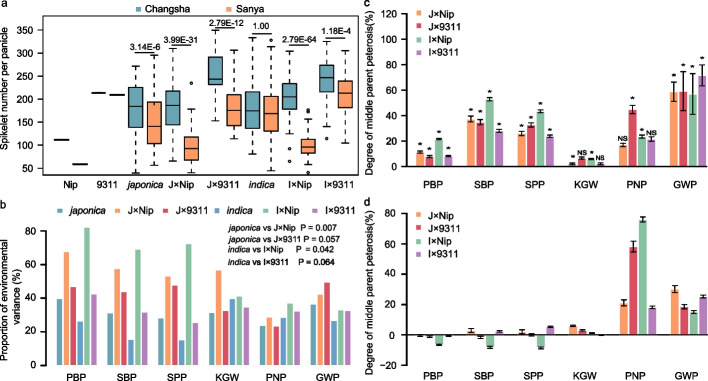
Architecture of yield traits and heterosis among 418 combinations. **a** Spikelet number per panicle (SPP) of inbred parents and their hybrids in Changsha and Sanya. **b** Proportion of environment variance (including environment-additive and interaction of genetic by environment variance) for yield-related traits in panels of inbred parents and hybrids. **c** Degree of middle-parent heterosis of four types of combinations for yield-related traits in Changsha. **d** Degree of middle-parent heterosis of four types of combinations for yield-related traits in Sanya. J×Nip, J×9311, I×Nip, and I×9311 represent the four types of combinations for *japonica*×Nipponbare, *japonica*×9311, *indica*×Nipponbare and *indica*×9311, respectively. PBP, SBP, KGW, PNP, and GWP represent primary branch number per panicle, secondary branch number per panicle, 1000-grain weight, panicle number per plant and grain weight per plant, respectively. The labeled value in **a** is the *p* value of two-tailed heteroscedasticity *T*-test. The asterisk labeled in **c** means the significant level at 0.05 between Changsha and Sanya, and NS means the difference is not significant

Obviously, both the phenotype of inbred parent and hybrid were influenced by the environmental condition; however, the influence of environment on hybrid was generally stronger than that of inbred parents (Fig. 1a). All six traits exhibit the same phenomenon, except for KGW of *indica* hybrids and PNP of *japonica* hybrids in Changsha and Sanya, respectively (Additional file 1: Figure S3c-d). To further investigate the environmental effect on yield traits of inbred parents and hybrids, we first evaluated the correlations of phenotypes between the two environments. The result showed that the correlation of parental phenotype between the two environments is generally much higher than that of hybrid except for PNP and GWP trait, which show a larger proportion of residual errors than the other four traits (Additional file 1: Figure S4a), implying that hybrids are more variable than inbred parents across environments. We then performed the two-way analysis of variance (ANOVA) including environment as a factor. The results indicated that the proportion of environment effect on hybrids was generally much higher than that on the corresponding inbreds, especially for SPP and its related traits (Fig. 1b and Additional file 2: Table S3). These observations also indicated that hybrids are more sensitive to the environment than their parents. Considering that there were only two environments in the current study, there may be deviations; hence, in order to ascertain whether this observation is a common phenomenon, we further analyzed the published data of 2808 maize hybrids and their parents across five environments [37], 265 maize hybrid and their parents across 4 environments [38], and 1800 wheat hybrid and their parents across 11 environments [39], the results showed that the effect of environment on hybrids was generally stronger than that of the inbred parents (Additional file 1: Figure S5). Taken these observations together, we concluded that hybrids are more sensitive to the environment than their inbred parents is a predominant phenomenon across species.

Examining the strength of heterosis in terms of different traits, combinations, and environments, the result indicated that heterosis, especially the better-parent heterosis, are just the potentiality rather than the inevitable result of hybridization, despite which is predominant over the cases (Additional file 1: Figure S6). It was clear that not all combinations showed hybrid vigor. On average, 10.65% of intra-subspecific hybrids and 10.29% of inter-subspecific hybrids even displayed hybrid weakness (Additional file 1: Figure S6a-h). The degree of middle-parent heterosis (dHmp) from all combination types, in terms of SPP-related trait and GWP, ranged from 7.87 to 70.13% with an average of 35.7% in Changsha, this was apparently higher than that in Sanya (−8.53 to 53.30% with an average of 8.21%) (Fig. 1c, d). However, for PNP and KGW, the dHmp of all combinations generally appeared to be weaker difference between the two locations (Fig. 1c, d). Particularly, the proportion of positive overdominant (POD) heterosis of all traits across the combinations in Changsha (averagely 63.19%) was much higher than that in Sanya (averagely 32.40%). And the decrease of POD from long-day to short-day environments was distinctly represented by spikelet number-related trait (PBP, SBP, and SPP), compared to the other traits (Additional file 1: Figure S6a-h). Compared to the intra-subspecific combinations across two environments, the proportion of POD heterosis of inter-subspecific combinations for all traits was apparently higher than that of intra-subspecific combinations in Changsha, except for the PNP trait with consistent heterosis across the two environments (Fig. 1c and Additional file 1: Figure S6a-d). In contrast, for SPP-related traits and GWP, the proportion of POD heterosis of inter-subspecific combinations was even lower than that of intra-subspecific combinations in Sanya (Fig. 1d; Additional file 1: Figure S6e-h). These lines of evidence indicated that the degree of heterosis is apparently dependent on environments, traits, and combinations. Thus, it is of significance to uncover the genetic basis and mechanism underlying inbreds, hybrids, and especially heterosis so as to highlight the opportunity to produce strong heterosis and elite hybrids.

### Genome-wide identification of QTLs affecting yield traits of rice hybrids

We carried out genome-wide association studies (GWAS), using 120 sets of genetic and phenotypic data. The phenotypic data consists of three types of datum panels evaluated for six yield traits (PBP, SBP, SPP, KGW, PNP, and GWP) under two environments (Changsha and Sanya). The three types of datum panels include 20 sets of data for each of the traits, i.e., (1) four sets from parents (both *japonica* and *indica* in two environments), (2) eight sets from F_1_ (four types of combinations in two environments), and (3) eight sets of calculated middle-parent heterosis value (Hmp) (four types of combinations in two environments) (see “Materials and methods”). The Manhattan plot of the results of association analysis is shown in Additional file 1: Figure S7 to S18.

Totally, we identified 635 and 624 QTLs in Changsha and Sanya, respectively, from the parental datum panel (P_QTL), 828 and 895 QTLs from the F_1_ datum panel (F_1__QTL), and 636 and 818 QTLs from the Hmp datum panel (Hmp_QTL) (Additional file 2: Table S4). When comparing the two environments, P_QTLs appeared apparently to be more environment-stable (proportion of shared QTLs, 38.4% on average), than F_1__QTLs (9.8% on average) and Hmp_QTLs (6.6% on average), regarding the traits related to grain number (PBP, SBP, and SPP) and grain size (KGW). As for PNP, the situation is combination-dependent. The three panels of QTLs related to grain weight per plant (GWP) were rather environment-specific (Additional file 1: Figure S19).

Comparing the shared QTLs from the three panels (P_QTL, F_1__QTL, and Hmp_QTL), we found that the genetic architecture affecting hybrids synchronizes more with that impacting heterosis (24.09±21%), compared to that affecting inbred parent (12.28±10%), but there were some exceptions for some combinations, environments, and traits (Additional file 1: Figure S20). The situation with more colocalized F_1__QTL and P_QTL than colocalized F_1__QTL and Hmp_QTL was more in Sanya than Changsha, more for spikelet number than PNP and GWP, more for 9311 combinations than Nipponbare ones. These results implied that the improvement of hybrids should concern both heterosis and the genetic background of the inbred lines, but their respective contribution varied depending on the combinations, environments, and traits.

### Nonadditive-preferred QTLs, which are more variable than additive-preferred ones, are the main contributors to heterosis

As expected according to the quantitative genetic theory, we can detect the QTLs showing significant additive effect in the parental datum panel, nonadditive effect in the Hmp datum panel, and both additive and nonadditive effects in the F_1_ datum panel. The above results indicated that one QTL can be detected based on different types of genetic effects (additive or nonadditive) in different types of panels by GWAS. Therefore, in order to estimate the relative degree of additive and nonadditive effect of each QTL and throw light on the understanding of the genetic basis underlying heterosis, we estimated the additive effect (a) and dominance effect (d) of each QTL on six yield traits using the parental, F_1_ and Hmp datum panels (see “Materials and methods” for detail). A QTL is referred as overdominance preferred if the absolute ratio of dominant effect to additive effects (|*d/a*|, degree of dominance) is no less than 1.5, and dominance preferred if 0.5≤|*d/a*|<1.5 (including partial dominance), and additive preferred if |*d/a*|<0.5 (see “Materials and methods” for detail). We called those QTLs being dominance preferred or overdominance preferred as nonadditive QTLs.

Among the 44 scenarios (five traits of four types of combinations under the two environments, plus GWP of two types of intra-subspecific combinations under the two environments), both F_1__QTLs and Hmp_QTLs showed apparently more nonadditive effects than P_QTLs (Additional file 1: Figure S21 and Additional file 2: Table S5), except for primary branch number per panicle of J×9311 in Sanya. Particularly, the majority of F_1__QTLs and Hmp_QTLs displayed overdominant effects (69.27% and 77.71%, respectively), with only a small portion of QTL represented additive effect (10.66% and 7.55%, respectively). Conversely, the majority of P_QTLs demonstrated additive (44.16%) and dominant (37.08%) effects, and only a small proportion (18.74%) showed overdominance. Consistent with the observation that SPP-related trait (PBP, SBP, and SPP) did not exhibit obvious heterosis in Sanya (Fig. 1d), fewer overdominant F_1__QTLs and Hmp_QTLs were identified in Sanya than that in Changsha. On average, 75.7% of the F_1__QTLs identified in Changsha expressed as overdominant for the SPP-related traits, while the proportion significantly reduced to 42.6% in Sanya (Additional file 1: Figure S21a-c). Comparing the two subspecies, we found that the reduction is more remarkable in *japonica* hybrids (from 71.0% in Changsha to 22.3% in Sanya) than that in *indica* hybrids (from 80.4% in Changsha to 62.8% in Sanya) (Additional file 1: Figure S21a-c). These observations indicated that the effects of environment on SPP-related trait in *japonica* hybrid was stronger than that in *indica* hybrid. When regarding the trait of KGW and PNP, the proportion of overdominance identified in F_1__QTLs and Hmp_QTLs did not show such consistent changes between subspecies and between environments, and varying by combinations (Additional file 1: Figure S21d-e).

To compare the response of different genetic components to the environment and their genetic background, comparative analysis from the levels of environmental and genetic background were conducted. First, we examined the environmental stability among the QTLs of additive, dominant, and overdominant ones, the result showed that a larger proportion of additive QTLs showed environment-stable than nonadditive QTLs, regarding most types of combinations for all traits except for PNP. Meanwhile, a higher proportion of dominant QTLs showed environment-stable than overdominant QTLs for most of the combinations and traits (Additional file 1: Figure S22). These results indicated that the higher magnitude of the dominant effect of a QTL, the stronger its environmental sensitivity. The distinctively larger proportion of unstable factors including overdominant and dominant QTLs identified in hybrids or heterosis than that in inbreds, consistent with the fact that the response of hybrid to the environment was generally stronger than that of inbreds (Fig. 1b and Additional file 1: Figure S4 and S5).

Second, in order to compare the stability of the genetic effect for each QTLs with additive, dominant, and overdominant under different genetic background (different combinations), we estimated the phenotypic coefficient of variation of the same genotype under different genetic backgrounds of individuals in the tested population, the result showed that homozygous genotypes of QTLs with nonadditive effects exhibited higher variability, as compared to those with additive effects (Additional file 1: Figure S23). Examining the variance of QTLs identified in an immortalized F_2_ population, in which the frequency of each genotype was more balanced, a similar phenomenon was observed (Additional file 1: Figure S24) [31]. Thus, these observations implied that the higher degree of dominance exhibited stronger background response and variability for most of the QTLs.

### Nonadditive expressed genes are highly dependent on the expression of their upstream transcription factors

**Fig. 2:**
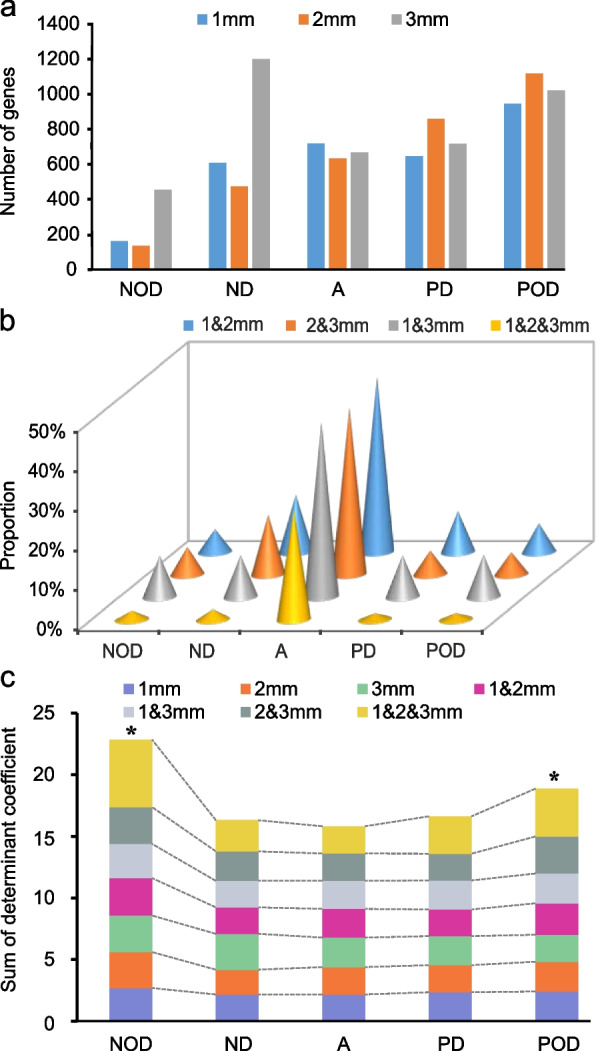
Evidences for background dependence of nonadditive phenomena at the transcriptional level. **a** The expression patterns in 1, 2, and 3mm of rice young panicles among 9311, Peiai 64s (PA64S), and their hybrid Liangyoupei 9 (LYP9); A, D, OD, POD, and NOD represent the expression patterns, additive, dominant and overdominant, positive overdominant, and negative overdominant, respectively. **b** Consistency for different expression patterns among different tissues (including 1, 2, and 3-mm young panicles) in the combination of LYP9. 1&2mm means the same expression pattern in 1 mm and 2 mm panicles, and similar for other symbols of 2&3mm and 1&2&3mm. ND and PD mean negative and positive dominant effect, respectively. NOD and POD mean negative and positive overdominant effect, respectively. **c** Sum of determination coefficient between transcription factors and their target genes with different expression patterns in rice young panicles among 9311, Peiai 64s (PA64S), and their hybrid Liangyoupei 9 (LYP9)

As mentioned in introduction and above, the heterosis, dominant and overdominant phenomenon at the phenotype level are often resulted from the integrated effects of multi-factors at various intermediate and fundamental levels (such as different genes, QTLs, gene expression, and physiological traits), thus it is challenging to investigate the molecular mechanism of heterosis at the phenotypic level. Transcription is such an intermediate step for a gene to perform its functions in development of complex phenotypes. Therefore, it is informative to explore gene expression patterns between parents and their F_1_ hybrid, in order to understand molecular mechanisms underlying heterosis. Here we investigated transcriptome profile of young panicles from the hybrid LYP9 and its two parents PA64S and 9311. As a whole, 8248 genes showed differential expressions between the two parents and their F_1_ hybrid in at least one of three tissues (1 mm, 2 mm, 3 mm young panicles). Expression patterns can be classified as additive preferred (A) (13%), dominant preferred (D) (39%), and overdominant preferred (OD) (48%) in at least one of three tissues (Fig. 2a, Additional file 2: Table S6). When compared the stability of genes with additive and nonadditive expression, we found that the dominant and overdominant expression showed dramatically more variability across tissues than the additive expression (Fig. 2b); in another words, nonadditive or heterosis of expression is more tissue-specific and may be more background-dependent. This is consistent with the results mentioned above, where hybrids are more variable than the inbred and nonadditive QTLs are more variable than additive QTLs.

We investigated the possible direct relations between nonadditive effect and genetic background by analyzing the correlation of expression levels between the target genes and their direct background, i.e., the transcription factors. The results indicated that the expression of genes with dominant and overdominant effects represented apparently stronger dependency on their upstream transcription factors than those genes with additive effects (Fig. 2c). We also analyzed the transcriptomes of three *Arabidopsis thaliana* combinations and observed the same phenomenon (Additional file 1: Figure S25) [12]. Thus, these results indicated an important phenomenon that expression of genes with nonadditive effects are more sensitive to the dosage changes of their upstream genetic backgrounds.

### One core molecular mechanism of dominance and overdominance—homo-insufficiency under insufficient background (HoIIB)

Does the genetic background dependency of nonadditive effects represent an essential molecular mechanism underlying heterosis? It is well known that no factor is absolutely independent in the biology system and that ligand-receptor binding, including the binding of transcription factor to a target gene, is obviously the most common dependent relationship between molecules, where the ligand and receptor can be the genetic background of one another and their binding reaction is described by the Hill equation [41]. In order to investigate the possible internal relationship between genetic background and the occurrence of dominance and overdominance, simulated genetic effects of one polymorphic site of one receptor were compared among the diploid parents and their F_1_, according to the Hill equation with different ligand concentrations as the background. We here considered the following three major regulation scenarios with the assumption that the ligand concentration is consistent among parents and their F_1_ for simplicity (see Additional file 3: Simulation1 for details).

Scenario 1: Null allele vs one functional allele of one polymorphic site under one genetic background, that is, one of two alleles of one polymorphic site of the receptor is loss-function and the other allele can be bound by one ligand as the background of the receptor (Additional file 1: Figure S26).

**Fig. 3:**
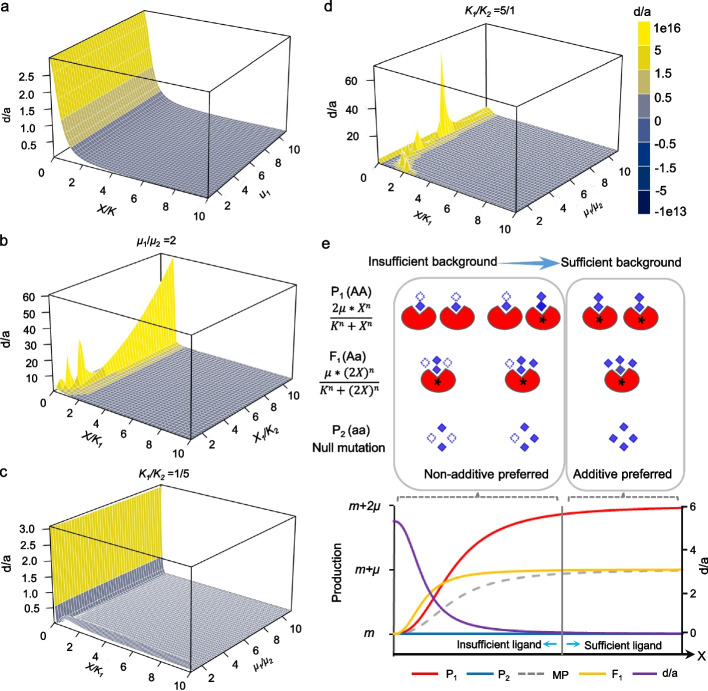
The diagram of Hill reactions illustrates the model of homo-insufficiency under insufficient background (HoIIB). **a** The simulated dominant degree of the target site under the activator background with different sufficiencies (X/K) and different *μ*_1_ with null allele and one functional allele under one regulator background. **b** The simulated dominant degree of the target site with same homologous backgrounds, but the two alleles in F_1_ are regulated by different factors in the background for positive regulation. **c** The simulated dominant degree of the target site with the same positive regulators or responsors as the background when allele 1 showing larger maximum function and higher affinity and allele 2 showing smaller maximum function and lower affinity (*μ*_1_ > *μ*_2_ and *K*_1_ / *K*_2_=1/5). **d** The simulated dominant degree of the target site with the same positive regulators or responsors as the background when allele 1 showing larger maximum function but lower affinity and allele 2 showing smaller maximum function but higher affinity (*μ*_1_ > *μ*_2_ and *K*_1_ / *K*_2_=5/1). **e** The red notched ellipse represents the target factor, and that with black star * indicates the target factor whom is activated by its activators (as the background of the target factor), which are represented by the blue diamonds. Here, we assume that one allele of genotype A can be bonded and activated by at least two units of activators, but the allele of genotype a is loss-function and cannot be bonded by the activator, and the activators can be randomly attached by each of two alleles in homozygote AA. The dotted blank diamonds are the required units of activators to activate all two alleles of AA in parent 1 (P_1_). The target factor will show overdominance (OD), where the production of heterozygote (orange line, F_1_) is higher than that of P_1_ (red line), when the quantity of activator is too insufficient to activate even one allele of P_1_ but can activate the allele A in F_1_ in most cases; and the target factor will show (partial-) dominance (D), where the production of heterozygote is higher than the middle-parent (grey dotted line), when the quantity of activator is relatively insufficient to activate all two alleles of P_1_ but can activate the allele A in F_1_ in most cases; and when the quantity of activator is sufficient to activate all two alleles of P_1_, the target factor will show additive effect, where the production of F_1_ is similar to or equal to middle-parent (almost overlap between dotted grey line and orange line); so the dominance degree (*d/a*) of target factor (purple line) will decrease along with the increase of activator (i.e., from insufficient to sufficient). The parameters used here are *μ*=1, *n*=2, and *K* = 1

For the positive regulation, when the activator as the background is insufficient (smaller X/K) for the functional allele of the receptor, the receptor will express as positive (partial-) dominance. In contrast, when the background is sufficient (larger X/K), the receptor will express as additive effect (Fig. 3a). Apparently, it is the insufficient ligand background that can only activate partial function of two homo-alleles in parents, but relatively full function of one allele in the F_1_, which results in the positive (partial-) dominance. For the negative regulation, the performance is similar, but the receptor expresses as negative (partial-) dominance, when the insufficient ligand background can only suppress partial function of two homo-alleles in parents, instead relatively full function of one allele in the F_1_ (Additional file 1: Figure S27). It is common between positive and negative regulations that the reaction is dramatically more sensitive to the ligand (activator or repressor) concentration change under insufficient ligand background, where the (partial-) dominance is observed easily. It should be noted that there is no overdominance for this scenario, if no synergistic effect were involved (when *n* is equal to 1).

Scenario 2: Two alleles of one polymorphic site under two independent backgrounds, that is, two alleles of one polymorphic site of the receptor, can be bound by two respective and independent ligands as the backgrounds of the receptor (Additional file 1: Figure S28).

For the positive regulation, as expected in Scenario 1, the receptor easily appears positive dominance when the activator background for the allele with the larger maximum function of the receptor is insufficient (smaller X/K). As different from Scenario 1, we can also observe positive overdominance under Scenario 2, when the receptor in F_1_ can cumulate the effect from the (partial-) dominant allele with a larger function and that from the other allele with a smaller function. When both backgrounds of two alleles are sufficient (higher X/K), the receptor in both parents and F_1_ can express the full function as two alleles and one allele, respectively, as a result the receptor expresses as additive (Fig. 3b and Additional file 1: Figure S29). The performances of negative regulation are similar, but the receptor expresses as negative (partial-) dominance or overdominance under insufficient background (Additional file 1: Figure S30). It is common between positive and negative regulations that the reaction is dramatically more sensitive to the ligand (activator or repressor) concentration change under insufficient ligand background (smaller X/K), where the nonadditive effect is easy to be observed.

Scenario 3: Two alleles of one polymorphic site with shared background, that is, two alleles of one polymorphic site of the receptor can be bound by the same ligand as the background of the receptor (Additional file 1: Figure S31). But these two alleles may have different affinities (*K*) to the ligand and show different maximum functions (*μ*). Thus, we considered two situations: (1) One allele has higher affinity and shows a larger maximum function, and the other has lower affinity and shows a smaller maximum function (abbreviated as HALF/LASF) (Fig. 3c and Additional file 1: Figure S32). (2) One allele has higher affinity, but shows smaller a maximum function, and the other has lower affinity, but shows a larger maximum function (abbreviated as HASF/LALF) (Fig. 3d and Additional file 1: Figure S33). Before considering the above two situations, we found from the simulation that there is only additive effect if the ligand randomly and equally binds to two alleles (see Additional file 3: Simulaiton1). In spite of positive or negative regulations (Additional file 1: Figure S34 and S35), the performance is similar to scenario 2 that the reaction tends to appear nonadditive under insufficient ligand background, especially for the allele with a larger maximum function. The insufficient ligand background renders the reaction dramatically more sensitive to the ligand (activator or repressor) concentration, compared to the sufficient ligand background. But we can only observe the nonadditive effect, when the background is dramatically insufficient under HALF/LASF (Additional file 1: Figure S33). In addition, the degree of nonadditive effect is apparent weaker in the HALF/LASF situation, compared to the HASF/LALF, because in the latter situation the background in F_1_ can be reallocated to the allele with LALF from the allele with HASF when the background for the latter has been saturated (Additional file 1: Figure S32). Taken together, we suppose that overdominance results from the cumulation or compensation between the (partial-) dominance of the allele with a larger function and the effect of the other allele with a smaller function.

According to the above simulations, we put forward one model that explains a core molecular mechanism underlying the nonadditive effects and heterosis: homo-insufficiency under insufficient background (HoIIB) (Fig. 3e). As indicated by HoIIB, it is the genetic background insufficient to maximize the function of two homo-alleles in parents, but relatively or even completely sufficient to maximize the function of one allele in F_1_, thus resulting in the insufficient function of two homo-alleles in parents, but the relatively or completely sufficient function of one allele in F_1_, that renders the target locus nonadditive in effect, as contributing to heterosis. And there were three main features of this theoretic model, according to the simulation. First, the background insufficiency for the allele with a larger function is the driving force for nonadditive effects. What we see dominance and heterosis is not the consequence of a stronger heterozygous, but the consequence of the weakened parent with homo-allele of larger function. In other word, the observable function of two homo-alleles is lower than their maximum function due to insufficient background. Second, if there is no synergy (*n* = 1), the overdominance can only be found when both alleles are functional, which result from the cumulation or complementation between the (partial-) dominance of the allele with a larger function and the effect of the other allele with a smaller function (Additional file 1: Figure S33). Third, we observed one general phenomenon in the three scenarios mentioned above, that is, the reaction is dramatically more sensitive to the ligand (activator or repressor) concentration under insufficient ligand background, where the nonadditive effect is easy to be observed.

### The HoIIB model was supported by different levels of evidence

**Fig. 4:**
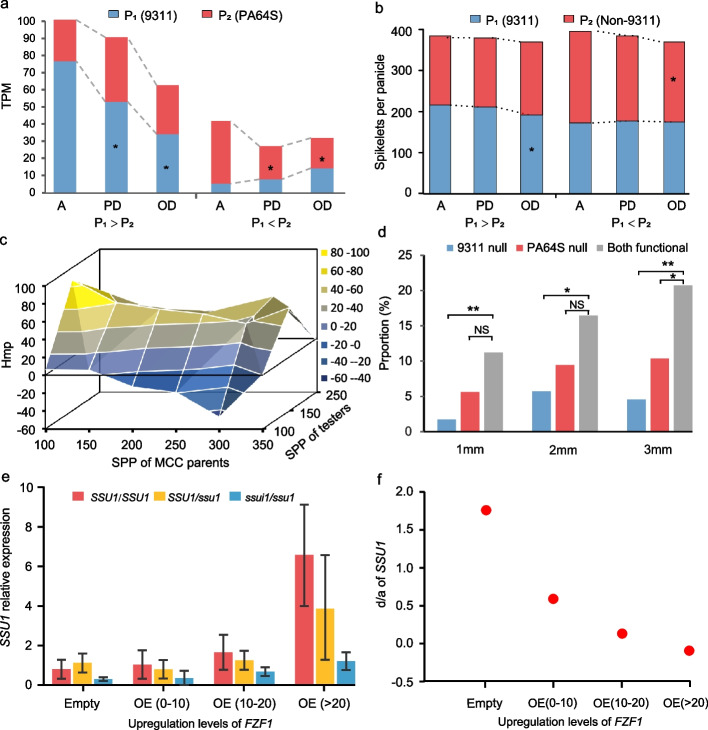
The background effect on additive, dominant, and overdominant effect at levels of transcription, QTL, and trait and the result of validation experiments conducted in *Saccharomyces cerevisiae.* **a** The expression of genes with different expression patterns in 3-mm young panicles of two parents; the star means significant difference from additive effect. **b** The effects of 9311 genotype (P_1_) and non-9311 genotype (P_2_) for QTLs of spikelet number per plant (SPP) with different genetic effect types for combination of *japonica*×9311 in Changsha; P_1_ > P_2_ means that 9311 genotype (P_1_) has higher effect than non-9311 genotype in QTL, and vice versa for P_1_ < P_2_. **c** The middle-parent heterosis of SPP for combinations MCC parents and testers with different SPP value. **d** The proportion of overdominance expressed genes in rice hybrid LYP9 under the condition of one parental allele is functional and both parental alleles are functional. Genes completely not expressed in the 1, 2, and 3-mm young panicles of 9311, but expressed in all of 1, 2, and 3-mm young panicles of PA64S were defined as 9311 null. Conversely, genes completely not expressed in the 1, 2, and 3-mm young panicles of PA64S, but expressed in all of the 1, 2, and 3-mm young panicles of 9311 were defined as PA64S null; The genes expressed in all of the 1, 2, and 3-mm young panicles of both 9311 and PA64S were defined as two parental alleles are functional (Both functional). The level of statistical significance was derived from the chi-square test. **e** The relative expression of gene *SSU1* in different *SSU1* genotypes under different expression levels of its transcription factor (*FZF1*) in Saccharomyces cerevisiae BY4743; here, *SSU1/SSU1*, *ssu1/ssu1* and *SSU1/ssu1* represent the homologous genotype of wild type, the homologous genotype of mutant, and their heterozygous genotype, respectively; OE (0–10) means the strain with upregulated *FZF1* by 0–10 folds, and similar for OE (10–20) and OE (>20), and Empty means the strain with empty vector free of *FZF1*. **f** The dramatically decreased dominance degree of *SSU1* along with the increase of upregulation levels of its transcription factor FZF1 in Saccharomyces cerevisiae BY4743

It is intriguing that in the observed experiments we have found extensive evidence that can represent the three features of the HoIIB model mentioned above. First, we observed the homo-insufficiency of the allele with a large function and the cumulation or complementation from the allele with a smaller function at various levels including transcription, QTL, and traits. Using transcriptome profile from the 1, 2, and 3 mm young panicles of 9311, PA64S, and their hybrids (LYP9), we investigated expression levels in the two parents for those genes with additive, positive dominant, and positive overdominant effects, respectively. The homo-insufficient expression was substantially observed in the higher parent for genes with dominant and overdominant transcription, compared to those with additive transcription (Fig. 4a and Additional file 1: Figure S36). Meanwhile, the homozygous genotypes in lower parent showed increased expression for the positive overdominance in most cases. Then we compared the QTL with different types of genetic effects that were identified by our GWAS of the three main yield components (SPP, KGW, and PNP). Apparently, the parents of genotypes with lager effects of the dominant and overdominant QTLs represented decreased phenotype, compared to those of the additive QTLs (Fig. 4b and Additional file 1: Figure S37 to S39). We also compared the QTLs that were identified by the 278 immortal F_2_ lines from the crosses between randomly selected RILs derived from Minghui 63 and Zhenshan 97 [31]. The four yield traits showed apparent HoIIB phenomenon for the dominant and overdominant QTLs, that is, the genotype with higher effect for dominant and overdominant QTLs represented decreased effect, compared to the additive QTLs (Additional file 1: Figure S40). We further investigated the distribution of the degrees of middle-parent heterosis for the five yield traits (SPP, PBP, SBP, PNP, and KGW) among the MCC combinations evaluated under the two environments. The stronger heterosis tended to be found among the combinations whose higher parents show decreased phenotypes (Fig. 4c and Additional file 1: Figure S41). Secondary, as predicted in HoIIB model, overdominance is more likely to occur when both alleles are functional. Examining the occurrence of overdominance in the transcriptomes of rice hybrid 9311×PA64S and *Arabidopsis* hybrid Col×C24, the results showed that overdominance was more frequently observed when both parental alleles were functional than that when only one parental allele was functional (Fig. 4d and Additional file 1: Figure S42). Thirdly, the HoIIB model implied that the expression or the observable function of those genes with stronger heterosis are subject to more serious homo-insufficiency background and thus will show a stronger response to the change of background, compared to those with weaker heterosis. This explains previous observation that the coefficient of variation of the QTLs identified in the MCC hybrid or by the immortalized F_2_ mapping panel showed that both homozygous and heterozygous genotypes of QTLs with (over-) dominant effects exhibited higher variability [31], compared to those with additive effects (Additional file 1: Figure S23 and S24). At the expression level, the instability of the genes with (over-) dominant expressions was reflected by their higher variance of expression across the 3 tissues, compared to the genes with additive effects (Additional file 1: Figure S43). Phenotypically, the combinations with higher degree of dominance also showed higher variability for most of the traits (Additional file 1: Figure S44).

### The HoIIB model was experimentally validated in yeast

To validate the HoIIB model, we designed an experiment to see whether we can manipulate the performance of heterosis of one gene by changing its background sufficiency within a living organism. In order to reduce the experimental complexity as much as possible, we used the transcription level as the performance indicator (phenotype) of the target gene and the transcription factor as its background, and carried out the experiment in the simple diploid organism, yeast. We screened the reported transcription factors and its target genes in yeast according to the following criteria: (1) the promoter region being bound by a transcription factor has been clearly validated; (2) there is strong and simple regulatory relationship between the transcription factor and its target gene. After investigating the co-expression of six pairs of genes (*WAR1* vs *PDR12*, *VHR1* vs *VHT1*, *VHR1* vs *BIO5*, *AZF1* vs *CLN3*, *AFT1* vs *FIT3* and *FZF1* vs *SSU1*), we found that *SSU1* showed a strong co-expression with its transcription factor FZF1 in strain BY4743 of *Saccharomyces cerevisiae* (*R*^2^ = 0.88, see Additional file 2: Table S7 and Table S8). So we selected *FZF1* and its target gene *SSU1*. According to the reported binding features between two genes [42], we knocked out the *FZF1* recognition motif 5′-CGTATCGTATAAGGCAACAATAG-3′ in *SSU1* promoter region, then constructed the heterozygous (*SSU1/ssu1*) and homozygous (*ssu1/ssu1*) knockout strain of *SSU1* in BY4743 (Additional file 1: Figure S45a-c). The *ssu1/ssu1* genotype showed apparently decreased expression compared to wild genotype of *SSU1* (*SSU1/SSU1*), indicating the effective mutation. Apparently, the *SSU1/ssu1* genotype showed obvious nonadditive expression (*d/a* = 1.899) in the system comprising genotypes *SSU1/SSU1*, *SSU1/ssu1* and *ssu1/ssu1* under normal *FZF1* background (Fig. 4e and Additional file 2: Table S9), implying that *FZF1* supply the insufficient background to *SSU1* in BY4743 according to our HoIIB model. We may expect that we can decrease the dominance degree of *SSU1* if we can regulate up the expression of its background *FZF1*. In the strains with native or overexpressed *FZF1*, we investigated the transcription level (representing the phenotype) of genotypes *SSU1/SSU1*, *SSU1/ssu1* and *ssu1/ssu1*, and the transcription level of *FZF1* (representing the background sufficiency). We really observed dramatically decreased dominance degree of *SSU1* along with the increasing of background, i.e., expression level of *FZF1*, and *SSU1* even nearly transited into additive expression when the expression of *FZF1* upregulated more than 10 folds (Fig. 4e, f). The results can be confirmed by a repeat experiment (Additional file 1: Figure S45d-e and Additional file 2: Table S10).

To validate our HoIIB model at a more complex phenotype level, we tried to investigate the effect of *SSU1* on growth rate of yeast. Given that *SSU1* may regulate the growth rate of yeast in the sulfur environment [43], we first examined the relationship between the transcription level of *SSU1* and yeast maximum growth rate in the liquid medium. It showed that the transcription level of *SSU1* was linearly correlated with the maximum growth rate of yeast, but when the transcription of *SSU1* was upregulated higher than 10 folds, the growth rate decreased to some extent. This indicates that high expression may not be beneficial to growth, and the relationship between them is complicated [44] (Additional file 1: Figure S46a-f). We thus selected the events with *SSU1* expression upregulated less than 10 folds to validate the HoIIB model. The HoIIB phenomenon was observed in all conditions including normal, 2 mM and 4 mM K_2_S_2_O_4_-treated SD-Ura medium (Additional file 1: Figure S46g-h and Additional file 2: Table S11). Thus, our designed experiments using both transcription and growth rate as the phenotype indicators indicated that the dominance degree of downstream genes can be manipulated by changing the level of background sufficiency.

### The systematic HoIIB phenomenon related to rice yield heterosis

The model and the results mentioned above revealed that insufficient background contributing to the homo-insufficiency is not only the limiting factor for (over-) dominant loci to reach their maximum function, but also the one that causes the instability of the target genes. Therefore, identification of (over-) dominant loci will provide us with a start point or hint to discover the key limiting factors along the genome, or gene regulatory network that impacts such important traits as yield, and thus guide the improvement of hybrids.

In order to investigate the possible systematic HoIIB factors impacting rice yield heterosis, we firstly compared the MCC QTLs identified from different combinations and environments (Additional file 2: Table S12), followed by gene set enrichment analysis using the candidate genes repeatedly identified by GWAS (Additional file 1: Figure S47). Results showed that the Nipponbare combinations have apparently more colocalized nonadditive QTLs than did the 9311 combinations, consistent with the fact that Nipponbare is less productive than 9311 and suggesting that Nipponbare may represent a more constrained background and thus easily result in nonadditive effect in its F_1_ hybrids compared to 9311. Regarding different subspecific combinations, negative overdominant QTLs were identified more frequently in *indica* combinations for traits related to SPP and PNP but in *japonica* ones for KGW; however, negative dominant and positive nonadditive QTLs tended to be detected in *japonica* combinations for all traits. Regarding different environments, the colocalized nonadditive QTLs tended to be detected in Sanya compared to Changsha (Additional file 2: Table S12). These results indicated that the HoIIB appeared to be taxa- and environment-systematic to some degree, but mainly determined by two specific parents in the combination investigated. Secondly, the GO enrichment indicated that those genes within additive QTLs seldom show enrichment, but those genes within nonadditive (dominant and overdominant) QTLs are frequently involved in many kinds of catalytic activities and binding functions (Additional file 1: Figure S48 and Additional file 2: Table S13). Compared to those genes with nonadditive performance in nonlethal deletion yeast strains grown in five different media [43], we also found that they enriched in the GO terms of catalytic activity (Additional file 1: Figure S49). The enrichment in catalytic activity for nonadditive genes may be explained by the reports that most enzymes in organism usually operate at an unsaturated substrate concentration [44], i.e., at the lower level of substrates, which may result in the insufficient background of these enzymes and thus their nonadditive performance. Further checking those genes encoding rate-limiting enzymes (RLE) showed that the proportion of RLE genes in nonadditive QTLs was generally higher than that in additive QTLs (Additional file 1: Figure S50), consistent with the fact that most of the RLEs usually contain the distinctly larger Kcat values compared to the available concentration of substrate [45]. These results suggested that the background/substrate of RLE may be the kind of important limiting factors that confer the systematically enriched catalytic activity in the pathway of these RLE genes. Thus, identifying and improving these limiting factors may provide the chance to make breakthroughs in future breeding of both inbreds and hybrids.

## Discussion

### HoIIB—a novel model revealed the core molecular mechanism underlying heterosis of single polymorphic locus

Utilization of heterosis has been a revolutionary technology in plant and animal breeding for a century. Regarding genetic basis of heterosis, three classical hypotheses, including dominance, overdominance, and epistasis, are well noted. As mentioned in the introduction, many lines of evidence have confirmed the appearance of those hypotheses. But different reports asserted different contribution of those “mechanism” to heterosis [10, 30, 46, 47], and as has pushed Kaeppler to insist “Heterosis: many genes, many mechanisms—end the search for an undiscovered unifying theory” [48]. In fact, there is impossibly heterosis if we cannot find dominance and overdominance [49], so we should recognize that understanding the internal mechanism of dominance and overdominance is the basic premise of understanding heterosis. In the present study, as evidenced by the results from yield heterosis of many rice hybrids, QTL mapping, and transcriptome profiling, as well as from the theoretical kinetic simulation, we propose one core molecular mechanism underlying heterosis of single polymorphic locus, the homo-insufficiency under insufficient background (HoIIB).

The HoIIB model suggests that the nonadditive effect is not the intrinsic feature of the gene under study; instead, the nonadditive effect is a phenomenon that two alleles of the homozygote show insufficiency in function under the insufficient background, but under which one allele of the heterozygote shows relative sufficiency in function. Under the HoIIB model, we can explain why the nonadditive QTLs are unstable across combinations and environments, because the nonadditive QTLs are under an insufficient background and thus easily subject to the changes of background or environment. In the present study, we have found extensive evidence that supports the HoIIB model, at the levels of phenotypes QTLs, transcription, and designed experiment in yeast. First, we observed apparent decrease in function for the homozygote of the allele with larger function under the situation of nonadditive effect, compared to the situation of additive effect. Second, we observed that when both alleles are functional, overdominance could be generated from the accumulation or complementation between the alleles with a smaller function and that with a larger function under the situation of background insufficiency. Third, the stronger response of a gene showing nonadditive expression to its transcription factors (TF), compared to that showing additive expression, suggested the greater impact of the insufficient genetic background exerted by the TF to the expression of its target gene. Indeed, besides TF vs its target as demonstrated in yeast experiment, the principle of HoIIB can also be extended to the actions between other biological molecules, such as kinase vs its target, small RNA vs its target, signaling molecule vs its target, and so on.

The HoIIB can not only give a unique explanation to dominance and overdominance, but also can interpret most known mechanisms, models, and phenomena about heterosis. The nonlinearity of the enzyme catalytic system was frequently described to explain heterosis [50]. But as our simulations indicated, the nonlinearity is not the absolute feature of enzyme catalytic activity, it just occurs when the substrate is insufficient to support the full function of the enzyme. In fact, the gene related to the enzyme can also be linear or additive when the substrate concentration is sufficient. BÄurger and Bagheri pointed that the output gain curve will change from non-linear to relative linear, and the dominance will transit to additive effect, if one mutation results in a decrease in Kcat that leads to a lower saturation level (i.e., A status that substrate saturates the enzyme at relative lower concentration level) [51]. For instance, genes with dl binding site are activated or repressed by dl at low threshold levels when dl has a low Kcat [52]. If *dl* null mutations possess larger Kcat, female flies heterozygous for *dl* null allele will express as dominant [53].^.^Of course, according to our HoIIB model, the additive effect also can transit to dominance or overdominance, when the background changes from sufficiency to insufficiency. In fact, sufficiency and insufficiency are relative and dynamic. The transition from insufficiency to sufficiency for one factor may cause new insufficiency for its counterpart factor. This may explain the challenge to the dominance and epistasis hypotheses, that is, why does heterosis not decrease along with the pyramiding of superior alleles [54].

The balance between genes involved in a biological complex system is another important hypothesis about heterosis. This hypothesis suggests that an imbalance in the concentration of the subcomponents of a protein–protein complex / pathway / network can be deleterious. The typical example of gene balance indicated that mutation of the subunit in a complex (or the factor in an interacting pair) can result in imbalance and thus is harmful, which might indicate the impact of gene imbalance on dominance [55]. Obviously, this hypothesis reveals an important mechanism of nonadditive under the situation that multiple factors have interactions. In this study, we simulated the genetic effects using the model of complex assembly to approximate the multifactor interaction, and investigate the influence of the background sufficiency on the target factors (Additional file 3: Simulation2). Our results showed that, if the background of the investigated subunit is non-bridging factor (such as A in ABA complex), nonadditive effect only occurs when the background is not sufficient, and it will become weaker and even loss when the background of the investigated subunit gets sufficient (Additional file 1: Figure S51a-c). Nevertheless, if the background of the investigated subunit is a bridging factor (such as B in ABA complex), the situation is complicated, both insufficient and over-supplied bridging factors may cause relative insufficiency of the background for one of the counterpart factors, which theoretically increases the possibility of dominance or overdominance [25] (Additional file 1: Figure S51d-e). These lines of observation indicated that heterosis is the result of low function of homozygote under insufficient backgrounds, rather than the heterozygote advantage.

There are plenty of other examples that indicate the dependency of heterosis on genetic background and can be explained by the HoIIB. First, in the comparisons of functional categories of enzymes, binding proteins, and transcription regulators, the proportion of haplosufficient genes (i.e., dominant genes) is the highest among genes that encode proteins with enzymatic functions [56], which is highly consistent with the fact that most of the enzymes work in low saturation levels, due to insufficient substrate. Second, an increased gene dose or gene mutations lead to an enhanced function, the metabolic background usually not synchronized with the target gene, which results in a more insufficient state of the background. A common observation is that the increased dose or function is not harmful, while its potential is severely unrealized in parent, due to the insufficient background. Thus, heterozygote is often observed as dominant [57]. Third, a decreased dosage or function often leads the background to a more sufficient state, compared to the original state, which frequently results in a dosage sensitive phenomenon, such as additive and haploinsufficiency [53]. In summary, our HoIIB is a fundamental mechanism and can interpret most models, hypotheses, and phenomena about heterosis. Of course, it is certain that there might be other complex situations beyond the scope of the HoIIB model, for instance, one complex trait can be nonadditive through the multiplication among additive subcomponent factors [58].

### The performances of rice yield gene *Hd3a* can validate the HoIIB model

In the main text, although we designed experiments in yeast to verify the HoIIB model, for rice, it is mainly derived from indirect evidences. In order to find the possible link between rice yield gene and HoIIB model, we set three standards to screen rice gene: (1) the regulation relationship between the target gene and its upstream gene had been clarified in literature; (2) the upstream gene is strongly affected by environment condition, and we thus do not have to take a long time to manipulate the background gene like the experiment conducted in yeast; (3) the genetic effect of the alleles had been verified and the gene expression level and phenotypic performance is linear correlated. We checked the heading date genes identified in SPP-related QTLs and found that *Hd3a* basically meets the above mentioned three criteria. Firstly, the expression level of *Hd3a* shows significantly negative correlation with SPP, and its expression level highly depends on its background regulator *Ehd1* (Additional file 1: Figure S52 a-c). Secondly, it was reported that under the long-day environment *Ehd1* is severely repressed and under the short-day environment *Ehd1* will be less repressed and keeps a relatively high expression level [59, 60]. So, *Hd3a* will be in a relatively insufficient background under long-day environment but sufficient background under a short-day environment, and we may expect that *Hd3a* will show nonadditive effect under a long-day environment but additive effect under short-day environment. We investigated the SPP performance of *indica*×Nipponbare hybrids in Changsha (long day) and Sanya (short day) and 1439 hybrids in Hangzhou (long day) and Sanya (short day) [32]. The results showed that *Hd3a* appears nonadditive phenomenon under long-day environment, where its background factor *Ehd1* is seriously insufficient (Additional file 1: Figure S52 d-e). And under short-day environment, where the background regulator *Ehd1* is relatively sufficient, *Hd3a* mainly expresses as additive (Additional file 1: Figure S52 f-g). The phenotypic performance of *Hd3a* under long-day and short-day environments is basically consistent with the expectation of the HoIIB model.

### Implication of the HoIIB model for genetic improvement of hybrid rice

The HoIIB model may affect future utilization of heterosis in several aspects. First, our HoIIB model indicated that in most cases heterosis is not the consequence of heterozygote advantage, but the homozygote disadvantage under insufficient background. This implies that current utilization of heterosis is not the best way to take advantage of maximum function of target genes [32]. Therefore, we need to identify and improve the constrained factor(s), or the target genes. In the present study, we extensively investigated yield QTLs or genes affecting parental lines, hybrids, and heterosis, followed by dissection of their genetic effect (Additional file 1: Figure S21). This may provide us with references to identify the limiting factors. As inferred from HoIIB model, when one factor was always detected as additive under different backgrounds, all backgrounds are sufficient, as implies that the potential function of the additive factor itself is lower than that of its backgrounds. When one factor was detected as additive under some backgrounds but nonadditive under other backgrounds, some backgrounds are sufficient and other backgrounds are insufficient, as implies that the potential function of the factor itself is lower than that of its backgrounds when additive but higher when nonadditive. According to this logic, the frequently detected additive factors may represent the systematic limiting factors that constrain the dominant or even overdominant factors from maximizing their functions, because the function (such as expression level) of the frequently detected additive factors is usually lower than those of the other factors in the regulation pathway; and in reverse, the frequently detected overdominant factors may represent the systematic limited factors (showing relative higher effect) that was constrained by the limiting factor, because the function (such as expression level) of the frequently detected overdominant factors is usually higher than those of the other factors in the regulation pathway. When comparing the frequently detected additive-preferred and overdominant-preferred QTLs affecting yield traits, we observed the expected results that the candidate genes within the frequently detected additive-preferred QTLs displayed distinctly lower expression, compared to those within the frequently detected overdominant-preferred ones (Additional file 1: Figure S53). Of course, lower expression just represents one aspect of the insufficient function of the genes, we may expect to observe the other aspects of insufficient functions, such as enzyme activity and affinity. For the positive regulation, when we upregulate the systematic limiting factor, we can release a batch of factors that were limited. For the negative regulation, it may be more efficient to downregulate the systematic negative factor than the non-systematic negative factor (the non-systematic factor implies higher potential function that cannot be easily downregulated or more alternative factors that can compensate each other). These results implied that the frequently detected additive factors, rather than the overdominant factors, should be focused in future breeding programs. Theoretically, we can easily make use of homozygote that can maximize the functions of the target genes of interest, which can be achieved by the improvement of the corresponding factors as the insufficient genetic background of target genes. Of course, further studies are needed to validate the claims related to the currently observed phenomena and results.

Second, although the above discussion may illude us to think that hybrid breeding is not necessary, our point is that utilization of heterosis will still be an important breeding strategy for a long time and even forever. Firstly, from the perspective of favorable alleles accumulation, even though all insufficient factors can be improved to their maximum functions, followed by integration into an inbred line in theory, it is impossible to be realized in a short time (Additional file 1: Figure S54). Secondly, it is an extremely long process to construct the regulatory network and thus clearly understand the mutual connection and dependency between genes. Thirdly, the mechanism of HoIIB implies that nonadditive is a common phenomenon in life system. The reason is simple, that is, it is not expected that the factors in a system operate on the exact required dependency on each other. Thus, one most insufficient factor will result in a batch of factors that present different degrees of homo-insufficiency. So we may expect to find less additive factors than nonadditive ones, including partial dominance, dominance, and overdominance. This is consistent with previous report and our observations [61]. We detected distinctly less additive QTLs (about 19%) than nonadditive ones (including partial dominance), and less genes with additive expression pattern (about 13%) than nonadditive ones. Fourth, genetic improvement is a dynamic process, involving the alleviation of insufficiency for one factor, followed by induction of insufficiency for the counterpart factor, that is, breakdown of old balance along with the establishment of new unbalance, plus the background change under different environments. For example, it has been proved that improvement of corn hybrids is mainly attributed to the improvement of their inbred parental lines. The high performance of inbreds did not decrease the degree of heterosis in hybrid corn breeding [1]. This phenomenon has also been observed in other organisms, such as cotton [54]. These results indicated that the dynamic breeding process contributes substantially for the continuous improvement of both inbreds and hybrids. Our results also indicated that we may consider different aspects, when we try to improve a variety. For example, we need to overcome the weakness of heterosis for SPP-related traits under short-day environment (Fig. 1d).

Third, the HoIIB model helps our understanding and utilization of general combining ability (GCA) and special combining ability (SCA), and provides guidance in breeding by genome selection. It was well known that additive effects contribute mainly to GCA, and nonadditive effects, including dominance and epistasis, to SCA [62]. Our HoIIB model suggested that the additive factors are less background-sensitive, compared to the nonadditive factors, which explain why SCA is more difficult to predict than GCA does. In addition, improvement of GCA through accumulation of additive superior alleles has proven to be an efficient strategy in hybrid breeding [63]. According the HoIIB model, the accumulation of additive superior alleles can definitely improve the genome background and thus release the potential functions of those limited factors. Our current study may suggest one possible and efficient strategy, in order to make breakthrough in hybrid rice breeding: (1) keeping on accumulation of superior alleles of the frequently identified additive factors, and try to improve them through both traditional and biotechnological methods, in order to continuously improve the genome background; (2) incorporating more subtle background effect into the model of genome selection in breeding for hybrids, in order to improve the prediction accuracy of special combining ability.

## Conclusions

To address the century-old mystery of heterosis, we assembled a large-scale of hybrid phenome, genome, and transcriptomic data to explore the mechanism. From these assembled data, we observed an important but common phenomenon that nonadditive factors are more background or environment dependent than that of additive ones. Further dynamic simulation combined with experimental results demonstrated a core molecular mechanism underlying heterosis, i.e., homo-insufficiency under insufficient background (HoIIB). The HoIIB elucidates that: heterosis in most cases is not the heterozygote advantage but the homozygote disadvantage under the insufficient genetic background; the limiting factor(s) generally exist along with the nonadditive factor(s), and the repeatedly identified additive ones must be the systematic limiting factors, which should be focused in future genetic improvement. HoIIB model can explain most known hypotheses and phenomena about heterosis, thus providing a novel informative guidance and perspective for the future hybrid breeding.

## Materials and methods

### Parental varieties and their F_1_ population construction

We used 265 world-wide varieties from the mini-core collection (MCC) of cultivated rice [35] as the parents to construct the F_1_ population. The F_1_ population was constructed by the crossing between two testers (temperate *japonica* variety Nipponbare and *indica* variety 9311) as female parent and the varieties in MCC as male parent. It took us five seasons to generate 455 combinations and to exclude the false crossing, we documented the false hybrids by comparing the phenotypic differences between hybrids and the corresponding female parent and further surveying the phenotypic segregation in the F_2_ population of each combination. Finally, we used 418 combinations with at least 100 F_1_ seeds for each combination in this study (Additional file 2: Table S2).

### Resequencing and genotyping for parental varieties and their F_1_ population

The parental varieties were resequenced as part of the 3000 rice genome project [64]. Genomic DNA was prepared from the leaves of a single young plant for each variety by a modified CTAB method. After the quality control, at least 3 μg genomic DNA of each sample was randomly fragmented by sonication and size-fractionated by electrophoresis, and DNA fragments of approximately 500 bp were purified. Each sequencing library was sequenced in six or more lanes on the HiSeq2000 platform, and 90 bp paired-end reads were generated. Subsequently, the reads from each sample were extracted based on their unique nucleotide multiplex identifiers as 83 bp reads (90 – 6 – 1, where 1 is the ligation base “T”). To ensure high quality, the raw data was filtered by deleting reads having adapter contamination or containing more than 50% low-quality bases (quality value ≤ 5).

The 83-bp paired-end reads of 267 rice varieties were mapped to the temperate *japonica* Nipponbare reference genome (IRGSP-1.0) using the BWA software with default parameters except for “aln –m 10000 –o l –e 10 –t 4”. The alignment results were then merged and indexed as BAM files [65]. SNP calling was based on the alignment using the Genome Analysis Toolkit 2.0-35(GATK) and Picard packages V1.71 [66]. To minimize the number of mismatched bases for SNP and InDel calling, all reads from each accession were further cleaned by (i) deleting the reads that unmapped to the reference in the alignment result, (ii) deleting duplicate reads, (iii) conducting alignment by the IndelRealigner package in GATK, and (iv) recalibrating realignment using the BaseRecalibrator package in GATK.

SNP and InDel calling for each sample were conducted independently using the UnifiedGenotyper package in GATK with a minimum phred-scaled confidence threshold of 50, and a minimum phred-scaled confidence threshold for emitting variants at 10. The SNP and InDel calling at the population level was performed using the UnifiedGenotyper package in the GATK pipeline with 50 for the minimum phred-scaled confidence threshold for variant calling and 30 for the minimum phred-scaled confidence threshold for variant emitting. Genotypes of the 267 rice varieties were called at the SNP sites. For the genotype datasets of all the accessions, SNPs with more than 50 % missing data and SNPs with MAF < 2% were excluded and 4,625,141 high-quality SNPs were generated. For the genotype datasets in each subspecies, SNPs with MAF < 2% were excluded and finally 3,562,187 and 1,649,161 high-quality SNPs for *indica* and *japonica* subspecies were generated respectively.

The F_1_ genotypes for each combination were inferred by the genotypes of their parents.

### Population genetic analysis

The phylogenetic neighbor-joining tree and principal component analysis were used to infer population structure of the parent panel. A pairwise distance matrix derived from the simple matching distance for 1.3 million SNP sites was calculated to construct unweighted neighbor-joining trees using the software MEGA5.0. Based on the neighbor-joining tree of 267 varieties in parental panel and the posterior validation errors in different number of run K in admixture, we divided the total panel into two major subspecies, i.e., *japonica* and *indica* subspecies (Additional file 1: Figure S1).

### Phenotyping of the parental varieties and their F_1_ population

We planted the 418 F_1_ hybrids and their 267 parents in 2013 at respective Changsha (CS) (28° 13′ N, 112° 58′ E, a long-day environment) and Sanya (SY) (18° 10′ N, 109° 28′ E, a short-day environment) of China. One combined plot including the F_1_ and the corresponding parents for each combination was planted with randomized complete block of two replicates in each environment. Each combined plot included five rows consisting of two testers (Nipponbare and 9311), F_1_ and MCC parent in sequence. The row and plant distances were 29.5 and 16.7 cm respectively, with 10 plants in each row, being wider than the general field production so as to decreasing the interface among plants as much as possible.

The yield-related traits were measured in two environments for each combination as follows. Five healthy plants in the middle of each row were used to measure six yield traits. The panicle number per plant (PNP) and grain weight per plant (GWP) was the average of all five plants. And we selected the main panicles of five plants to count the spikelet number per panicle (SPP), the secondary branch number per panicle (SBP), and primary branch number per panicle (PBP). The 1000-grain weight (KGW) was rescaled by the grain weight of 300 grains selected from five main panicles.

The middle-parent heterosis value (Hmp) for each trait of each combination was measured as: *F*_1_ − (*P*_1_ + *P*_2_)/2, i.e., the deviation of F_1_ from middle-parent performance, where F_1_, P_1_, and P_2_ represent the phenotypic values of each trait in F_1_, P_1_, and P_2_ respectively. Degree of middle-parent heterosis (dHmp) for each trait was measured as: (*F*_1_ − (*P*_1_ + *P*_2_)/2)/(*P*_1_ + *P*_2_)/2). In addition, we denoted the positive overdominant heterosis (POD) when the F_1_ shows the phenotypic value over the higher parent, range between the two parents (RBP) when the F_1_ shows the phenotypic value between two parents and negative overdominant heterosis (NOD) when the F_1_ shows the phenotypic value below the lower parent (Additional file 1: Figure S6).

### Estimation of environment and genotypic variance

For each variety, there are two environments and each environment has two replicate of phenotype data. Two parent population including *japonica* and *indica* subspecies and four types of hybrid combination (*japonica*×Nip, *japonica*×9311, *indica*×Nip and *indica*×9311, abbreviated as J×Nip, J×9311, I×Nip and I×9311) were used. The following linear model was fitted to the transformed data:

$${Y}_{ijk}=\mu +{G}_i+{E}_j+{G}_i\times {E}_j+{\varepsilon}_{ijk}$$

Here *Y*_*ij*_ is the *ij*th phenotypic observation for the *i*th rice variety under *j*th environment, *k* represents two replications, *μ* is the overall mean, *G*_*i*_ and *E*_*j*_ is the genotypic and environmental effect. *G*_*i*_ × *E*_*j*_ the genotypic and environmental interaction effect, *ε*_*ijk*_ is the random residual effects.

### Genome-wide association study (GWAS)

GWAS was conducted in GAPIT using the compressed mixed linear model (CMLM) [67]. The phenotype includes the trait value of parents in *japonica* and *indica* respectively, the F_1_ trait value of four kinds of combinations (J×Nip, J×9311, I×Nip and I×9311) respectively, and the middle-parent heterosis value (Hmp) of four kinds of combinations (J×Nip, J×9311, I×Nip and I×9311) respectively. For the CMLM analysis, we used the equation [67, 68]:

$$y= X\alpha + P\beta + K\mu +e$$

Here, *y* represents phenotype, *X* represents genotype matrix, *P* is the matrix of principal components, and *K* is the kinship matrix. *α* and *β* represent fixed effects of genotype and population structure, and *μ* and *e* represent random effects of kinship and residuals. The first five principal components were used to estimate the population structure. The matrix of genetic similarity based on simple SNP matching coefficients was used to model the variance-covariance matrix of the random effect.

To avoid the over correction of the Bonferroni method, 1000 permutation tests were used to estimate the significant *P* thresholds [69]. For each examined trait, we reshuffled the original phenotype data, and then performed association analysis with the same parameters. After 1000 permutations, we got 1000 association *p* value from permutation (p_per) for each SNP and we set the highest −log(p_per) as the FDR of that SNP. The SNP was denoted as significant association when the −log(p_GWAS) is larger than the highest −log(p_per), where p_GWAS is the association *p* value for each SNP for original phenotypic data.

### Estimation of additive and dominance effects for each significant SNP locus and QTL

Firstly, we estimated the additive and dominance effects for each significant SNP locus. We defined the tester’s genotype (Nipponbare or 9311) of each SNP as A, and the non-tester’s genotype as B. When some varieties in the MCC parental panel show as A and the others show as B, their F_1_ will show the genotypes A and H (the heterozygous genotype). For the investigated trait, we set *P*_*A*_ as the mean phenotype of parents with genotype A, *P*_*B*_ as that of parents with genotype B, *F*_*A*_ as that of F_1_ with genotype A, and *F*_*H*_ as that of F_1_ with genotype H. The additive effect of each SNP was half of the absolute difference between the two homozygotes, i.e., *a* = |*P*_*A*_ – (*P*_*A*_ + *P*_*B*_)/2|. The traditional estimation for dominance effect was expressed as *d* = *F*_*H*_ − (*F*_*A*_ + *F*_*B*_)/2, here we rescaled the dominance effects identified as *d* = *F*_*H*_ − (*F*_*A*_ − *P*_*A*_) − (*P*_*A*_ + *P*_*B*_)/2, in which *F*_*A*_ − *P*_*A*_ means the background heterozygous effects.

Secondly, in order to delimit QTLs, we firstly calculate LD blocks for all significant SNPs (SNPs showed higher association signal than the 1000 permutations) using GAB algorithm in Haploview 5.0 (with *r*^2^ ≥ 0.8). For each block, a tagSNP was selected from the largest group of strongly linked significant SNPs in that block [70]. If one block size was larger than 20kb and there were no less than 3 significant SNPs in the block, we defined the block as one QTL and the name of QTL was assigned by the ID of the selected tagSNP. Then, the additive and dominant effects of each QTL were estimated by the average additive and dominant effects of the significant SNPs within the QTL region.

Finally, considering that the degree of dominance in real data is usually continuous, it is difficult to make a rigid distinction between additive, dominant, and overdominant, we used a gradient division method to obtain an approximate description of additive, dominant, and overdominant genetic components. Specifically, we defined a QTL is referred to as overdominance preferred if the absolute ratio of dominant effect to additive effect (|*d/a*|, degree of dominance) is no less than 1.5, and (partial-) dominance preferred if 0.5≤|*d/a*|<1.5, and additive preferred if |*d/a*|<0.5. The dominant and overdominant QTLs can further be classified as positive ones when their *d* > 0 or negative ones when their *d* < 0.

### Investigation of phenotypic variation coefficients of QTLs in different genetic types

For each QTL, there are three genotypes, including two homozygous of AA and aa, and one heterozygous of Aa. We first calculate the phenotypic variation coefficient (phenotypic standard deviation / phenotypic mean) in each genotype, then taken the average of the phenotypic variation coefficient of these different genotypes (AA, Aa, and aa) as the phenotypic variation coefficient of the QTL. For each genetic type (additive, dominant, or overdominant), the average phenotypic variation coefficient of all QTLs in this genetic type is calculated as the phenotypic variation coefficient of QTL of this genetic type. According to the above calculations, the phenotypic variation coefficient of additive, dominant, and overdominant were obtained for each trait, and the cumulative value of all the investigated traits was used as the final result. The statistical significance between the ratio that the cumulative phenotypic coefficient of variation in dominant and overdominant QTLs was larger than that of in additive QTLs and the expected ratio (16:16) was obtained by chi-square test.

### Transcriptome in rice hybrid combinations and data analysis

The transcriptome was from the 1, 2, 3, and 4 mm young panicles respectively in combination of hybrid LYP9 and its parents (9311 and PA64S) in Changsha. The raw RNA-seq data of 9311, PA64S, and LYP9 were download from Genome Sequence Archive of Beijing Institute of Genomics, Chinese Academy of Sciences (gsa.big.ac.cn) under accession no PRJCA000131 [34]. All the reads were mapped to IRGSP1.0 using TopHat [71] software with parameters: minimum intron length of 20, maximum intron length of 10,000, and a maximum of two mismatches. Only unique mapped reads were extracted for the following analysis. The number of fragments per kilobase of exon model per million mapped reads (FPKM) for each gene was calculated using Cufflinks [72], and transcripts per million reads (TPM) were finally used to measure the expression level. Differentially expressed genes among two parents and the hybrid were identified using the R package DEGseq [73]. The expression patterns were determined as follows. Firstly, the standard deviation of TPM for each gene was estimated according to three replicates in 4 mm young panicles of 9311, PA64S, and LYP9, and two replicates in 3 mm young panicles of PA64S and LYP9. Secondly, the expression patterns were determined according to the significant different expression levels among 9311, PA64S, LYP9, and the middle-parents at significance level *p* =0.01. In detail, if LYP9 is significantly higher than the higher parent, the gene was classified as positive overdominance (POD); if LYP9 is significantly lower than the lower parent, the gene was classified as negative overdominance (NOD); if LYP9 is significantly higher than the middle-parent, but shows no significance from the higher parent, the gene was classified as positive dominance (PD); if LYP9 is significantly lower than the middle-parent, but shows no significance from the lower parent, the gene was classified as negative dominance (ND); if LYP9 is significantly higher than the middle-parent and significantly lower than the higher parent, the gene was classified as positive partial dominance (PPD); if LYP9 is significantly lower than the middle-parent and significantly higher than the lower parent, the gene was classified as negative partial dominance (NPD); if LYP9 is not significantly different from the middle-parent, significantly lower than the higher parent and significantly higher than the lower parent, the gene was classified as middle-parent (MP) or additive expression (A).

### Transcriptome in Arabidopsis hybrid combinations and data analysis

The raw RNA-seq data of Col×C24 Col-0×Per-1, Col-0×Aa-0, Col-0×Ak-1, Col ×C24, and their parents were downloaded according to the information provided by the original literature [12, 14]. Subsequent reads alignment, the quantification of gene expression, and the identification of expression pattern have followed the method and process as described above in rice combination LYP9.

### Estimating the determination coefficient between genes with additive, dominant, and overdominant expression and their upstream transcription factors

Rice or Arabidopsis genes were divided into additive, dominant, and overdominant expression modes according to the methods described in the above introduction. The upstream transcription factors of each gene were retrieved from the annotated database (http://plantregmap.gao-lab.org/), and the determination coefficient between genes with additive, dominant, and overdominant expression patterns and their upstream transcriptions factors were calculated separately.

### Construction of *SSU1* mutants in yeast

In this study, diploid BY4743 was used as the wild type experimental strain. In order to knock out the recognition motif of *FZF* in the promoter of *SSU1* gene, PCR amplification of vectors Pfa6a-Leu1Mx (Leu) and Pfa6a-His3Mx6 (His) were performed using primers (HRR-SSU1-F and HRR-SSU1-R) to obtain recombinant components. The recombinant component was verified by sequencing and then transformed into strain BY4743. After verification of positive clones by electrophoresis and sequencing, the heterozygous mutant strains (*SSU1/ssu1*) contain single-strand DNA substitution (Leu or His) and diploid mutant strains (*ssu1/ssu1*) that contain both Leu and His substitution were successfully constructed (Additional file 1: Figure S45a-c and Additional file 2: Table S7).

### Overexpression of transcription factor *FZF1* in yeast

RNA was extracted from BY4743 strain, and the coding sequence of *FZF1* was amplified from BY4743 cNDA and cloned into pAG416 vector by recombination methods. The constructed vector along with the empty vector pAG416GAL were transformed into *Saccharomyces cerevisiae* strain BY4743 (referred as *SSU1/SSU1*), two types of heterozyzous mutant strain (Het-leu and Het-his, referred as *SSU1/ssu1*), and diploid mutant strain (referred as *ssu1/ssu1*). The methods related to yeast cultures, transformations, and growth assay mainly referred to Gietz et al. [74]. Yeast cells were grown at 30°C in synthetic defined (SD) medium (0.67% yeast nitrogen base, Sigma) without amino acids, containing 2% (w/v) glucose or 2% (w/v) galactose (induction medium), supplemented with yeast synthetic dropout without histidine (Clontech, CA, USA), pH 5.8.

### qRT-PCR of *FZF1* and *SSU1*

Total RNA isolated from fresh yeast cultures and reversed transcribed using a protocol as previously described. RT-qPCR analysis was performed using gene-specific primers listed in Additional file 2: Table S7. *Saccharomyces cerevisiae* 18S RNA was used as reference genes to normalize the data.

### Evaluation of heterosis of *SSU1* expression under different *FZF1* expression levels

We used four kinds of strains including WT (referred as *SSU1/SSU1*), heterozygous mutant (referred as *SSU1/ssu1*), and diploid mutant (referred as *ssu1/ssu1*) which transferred the empty vector PAG416GAL as control. *FZF1* overexpression (FZF1-OE) strains were divided into 0–10, 10–20, and >20 groups according to the upregulation ratio between *FZF1* expression levels in FZF1-OE and empty event, referred as OE (0–10), OE (10–20), and OE (>20) (Fig. 4e). For each group, the average expression level of all strains with the same genotype was used as the expression of that genotype (Additional file 2: Table S9). In another parallel experiment, due to the fewer events of strain with high *FZF1* upregulation ratio, we grouped them into 0–5, 5–10, and >10 groups, referred as OE (0–5), OE (5–10), and OE (>10) (Additional file 1: Figure S45c-d and Additional file 2: Table S10). The formula of additive and dominance of expression quantity is as follows:

- Additive effect = |*SSU1/SSU1* − *ssu1/ssu1|*/2
- Dominance effect = *SSU1/ssu1* − (*SSU1/SSU1* + *ssu1/ssu1*)/2
- Degree of dominance *d/a* = Dominance effect /Additive effect

### Determination of maximum growth rate of yeast in liquid medium

Yeast cultures were incubated at 30°C. Liquid SD-Ura medium was used to keep the pAG416GAL plasmid during overnight growth. Three culture conditions were set in liquid medium, including normal SD-Ura medium, 2mM K_2_S_2_O_5_-treated liquid SD-Ura medium, and 4mM K_2_S_2_O_5_-treated liquid SD-Ura medium. Growth rate in liquid medium was monitored by determining optical density at 600 nm in a SPECTR Ostar Omega instrument (BMG Labtech, Offenburg, Germany), and the measurements were taken every 2 h for 2 days after 20s pre-shaking. Growth parameters were calculated from each treatment by directly fitting OD measurements versus time to the reparametrized Gompertz equation proposed by Zwietering et al. [75]:

$$y={A}^{\ast}\exp \left(-\exp \left(\left(\left(\mu {\max}^{\ast }e\right)/A\right)\ast \left(\lambda -t\right)+1\right)\right)$$

where *y* = ln(ODt/OD_0_), OD_0_ is the initial OD, and ODt is the OD at time *t*; *A* = ln(ODt/OD0) is the asymptotic maximum, *μ*_max_ is the maximum specific growth rate (h^−1^), and *λ* is the lag phase period (h). Phenotypic data were fitted to the reparametrized Gompertz model by non-linear least-squares fitting using the Gauss-Newton algorithm as implemented in the nls function in the R statistical software, v.4.0.

The formula of additive, dominance, and degree of dominance for maximum growth rate of yeast are the same as described in expression analysis.

### Phenotypic analysis of heterozygous and homozygous mutants in yeast

Steinmetz et al. measured growth rates of strains with precise deletions of each gene in the yeast genome using a parallel molecular bar-coding strategy [76]. We used their data (available at http://www-deletion.stanford.edu/YDPM/YDPM_index.html) for nonlethal gene deletion strains grown in YPD, YPG, YPDGE, YPE, and YPL media. Here, we consider only nonlethal mutations for which homozygous and heterozygous growth rate data are available on the media. For each media, we used the average performance of the top 10% as normal wildtype (WT), homozygous deletion of the strain as homozygous type (Hom), and the deleted gene in heterozygous strain recorded as Het. The additive effect, dominance effect and the degree of dominance was calculated as:

- Additive effect = |WT − Hom|/2
- Dominance effect = Het − (WT+Hom)/2
- Degree of dominance *d/a* = Dominance effect/additive effect

Here we defined the genes with |*d/a*| >0.5 as nonadditive performance, only the genes identified as |*d/a*| >0.5 in 4 or more than 4 kinds of media were used to further GO enrichment analysis.

### GO enrichment

GO analysis was performed using methods available at the agriGO website [77].

### Analysis of gene-related rate-limiting enzyme

The protein sequences of genes encoding rate-limiting enzyme (RLE) in human, mouse, and yeast were obtained from the database of RLEdb (http://rle.cbi.pku.edu.cn/) [45]. The candidate genes within additive, dominant, and overdominant QTLs that might encode RLE protein in rice were identified by protein sequence alignment though blastp algorithm (NCBI package blast-2.7.1) with expected threshold 1e−6. Totally, 2232 rice genes were identified as RLE-related genes according to RLEdb database.

The number of genes encoding the same rate-limiting enzyme between candidate genes within nonadditive (dominant and overdominant) QTLs and additive QTLs were compared with a two-tailed paired *T*-test (Additional file 1: Figure S50).

## Supplementary Information

**Additional file 1.** Figures S1-S54.

**Additional file 2.** Table S1-S13.

**Additional file 3.** Supplementary methods [84–87].

**Additional file 4.** Review history.

## Data Availability

The sequencing data of the 267 *O. sativa* accessions were obtained from the 3KRGP [78]. The genotypic data of 267 accessions and the phenotypic data of 418 rice hybrids are deposited in figshare [79]. The public data used in this article were downloaded from the original published papers. RNA-sequence data of rice hybrids 9311×PA64S were deposited Genome Sequence Archive (gsa.big.ac.cn) with accession number PRJCA000131 [80]. RNA-sequence data of Arabidopsis hybrids Col×C24, Col-0×Per-1, and Col-0×Aa-0 were deposited in GEO database with accession number GSE85759 [81], RNA-sequence data of Col-0×Ak-1 were deposited in GEO database with accession number GSE100595 [82], and the RNA-sequence data of Col ×C24 were deposited in GEO database with accession number GSE51578 [83]. Correspondence and request for materials and data should be addressed to H.Z. (zhangl@cau.edu.cn).
