# Supplementary material for: Large-scale genomic and transcriptomic profiles of rice hybrids reveal a core mechanism underlying heterosis
Source: Genome Biol. 2022 Dec 22;23:264. doi: 10.1186/s13059-022-02822-8 (PMC9773586; doi:10.1186/s13059-022-02822-8)
Supplement: Supplementary file 1 — Additional file 1. Figures S1-S54. [file 13059_2022_2822_MOESM1_ESM.pdf]

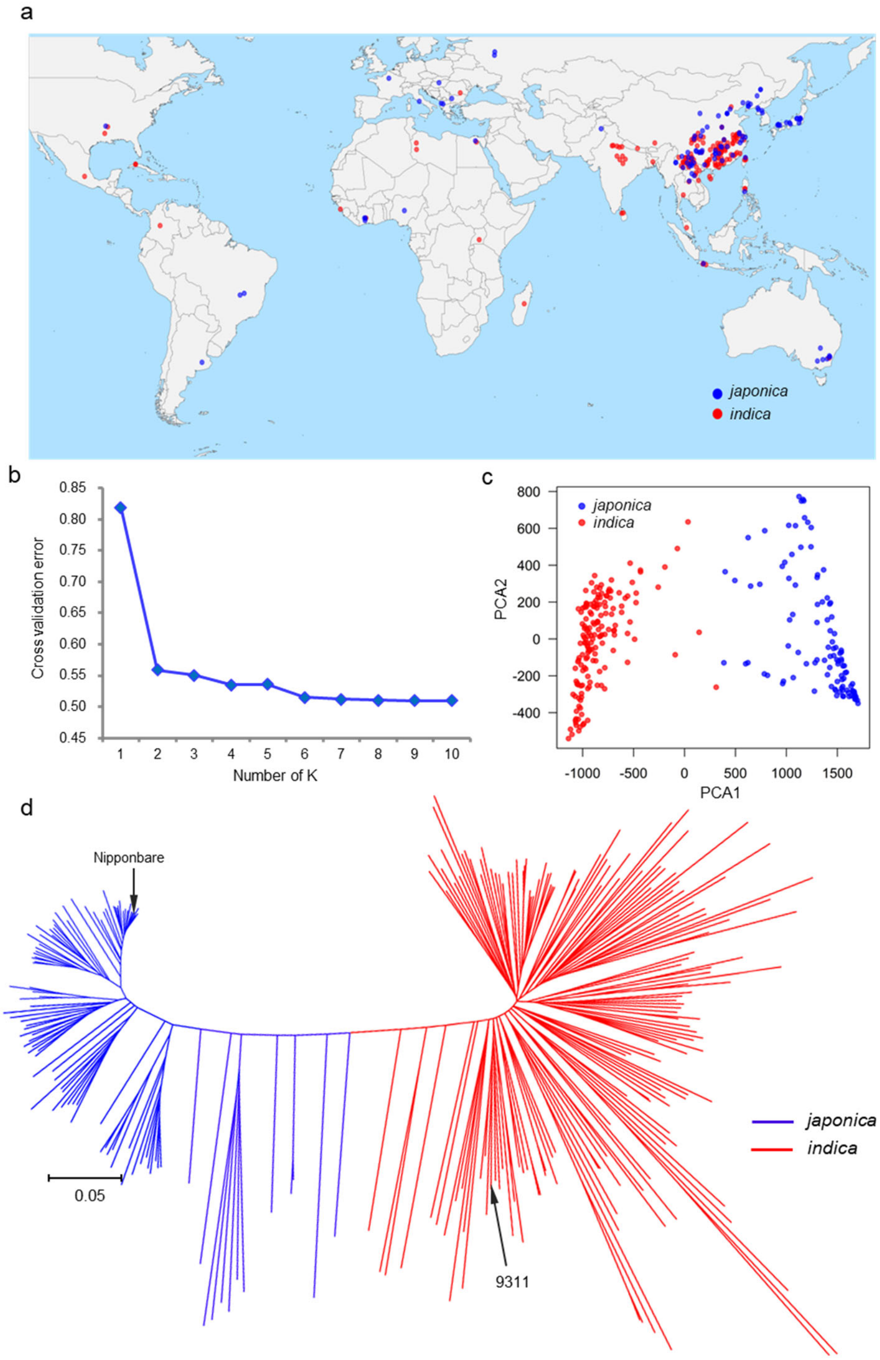


**Figure S1. Genetic structure of the 267 rice accessions.** (a) Geographical distribution of 267 rice varieties. the blue dots represent *japonica* varieties, the red dots represent *indica* varieties. (b) The posterior validation errors in different number of run K in admixture. (c) The PCA1 and PCA2 plot of *japonica* and *indica* subspecies. (d) The neighbor-joining tree of 267 rice accessions constructed from simple matching distance of 1.3 million SNPs. The blue and red lines represent *japonica* and *indica* accessions, respectively. Test lines Nipponbare and 9311are marked by black arrow.

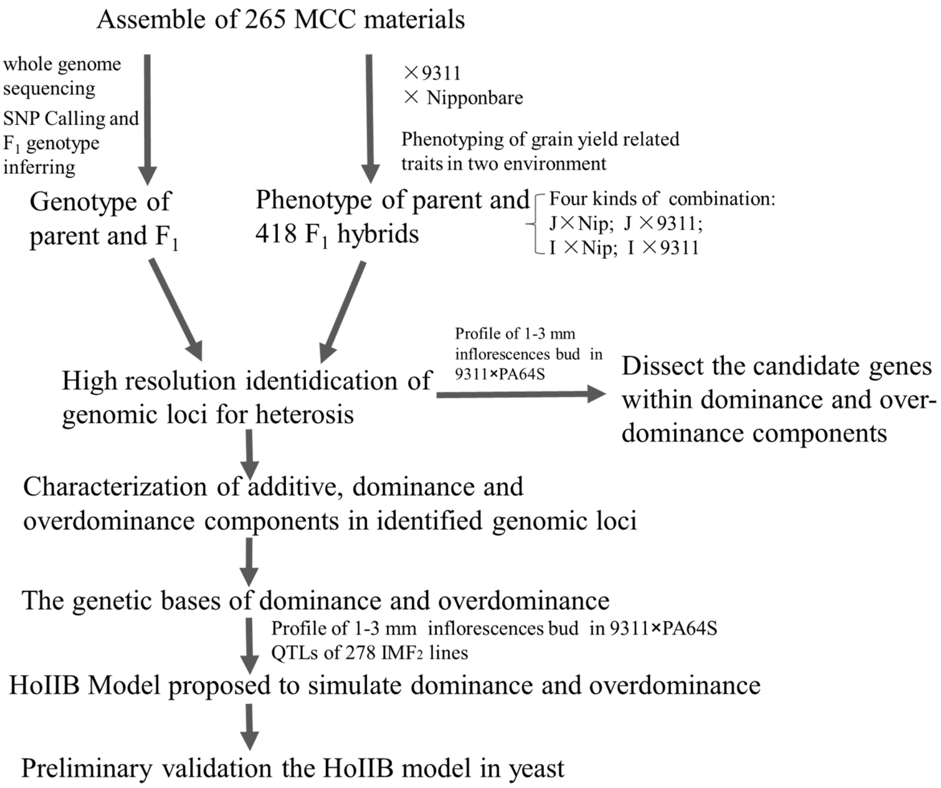


**Figure S2. The experimental design and analysis procedure used in this study.**

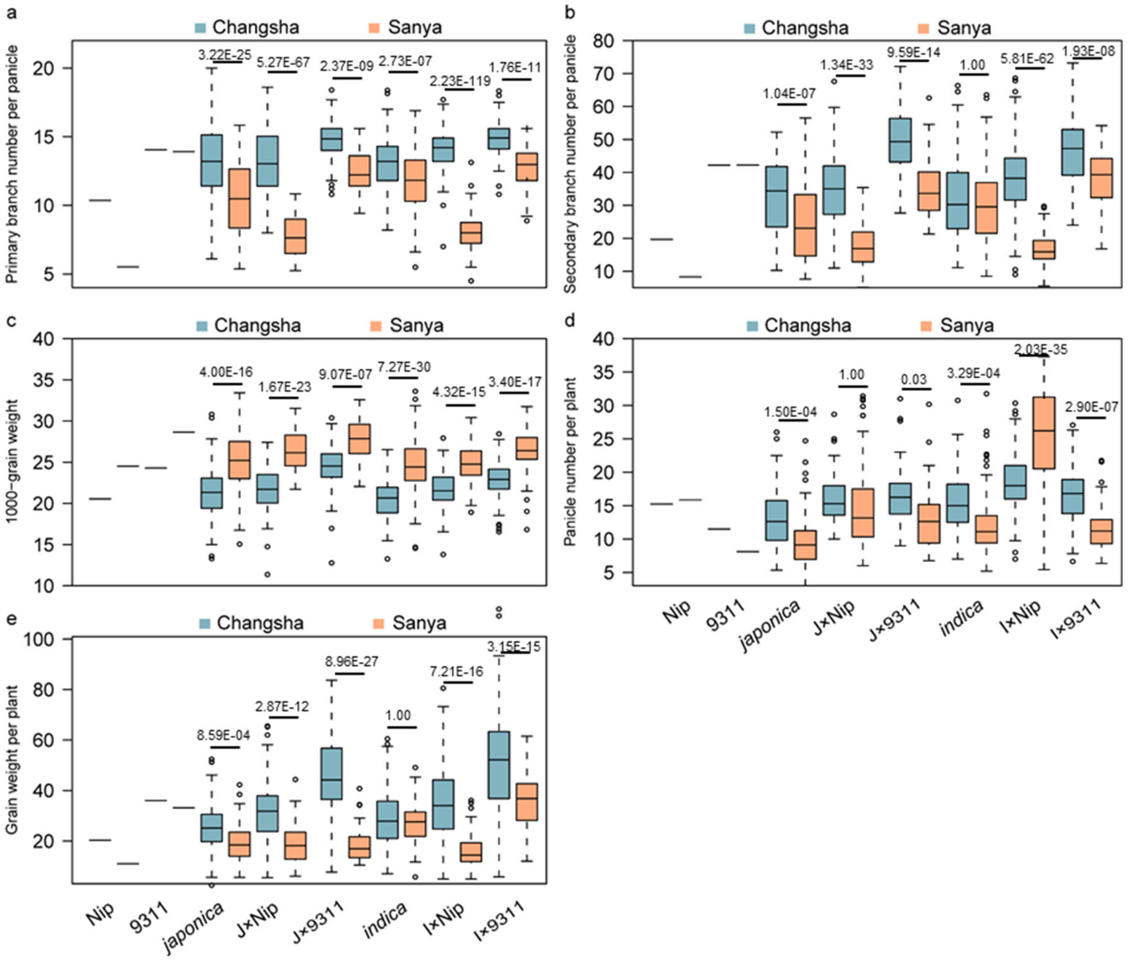


**Figure S3. The phenotypic distribution of parent and F1 for different yield traits in Changsha and Sanya.** Nip, Nipponbare; J×Nip, combination of *japonica* and Nipponbare; I×Nip, combination of *indica* and Nipponbare, and the others are similar. The labeled value in (a-e) is the P-value of two-tailed heteroscedasticity *T*-test.

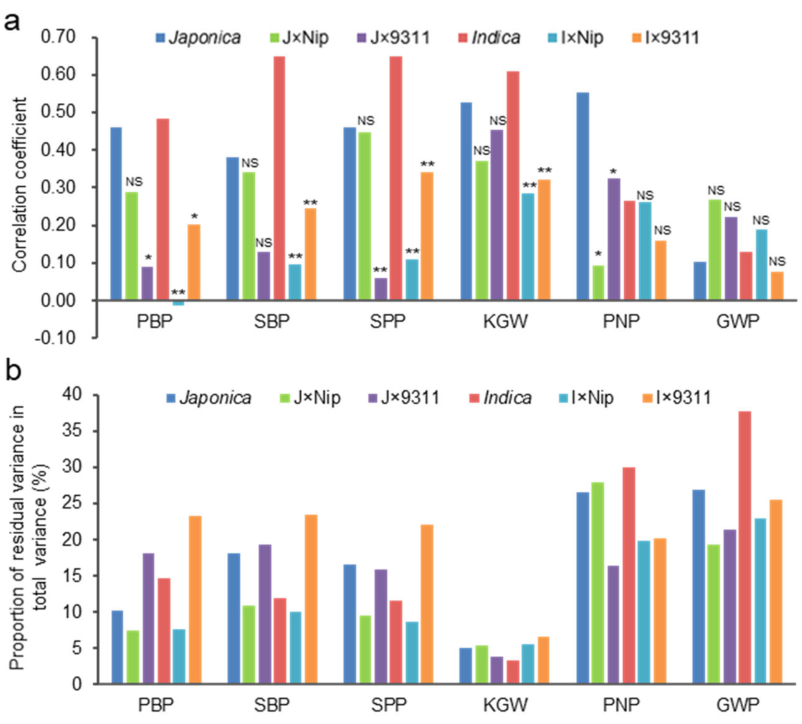


**Figure S4. The phenotypic correlation between Changsha and Sanya environment and the proportion of residual variance of yield trait in inbred parents and hybrids.** (a) The phenotypic correlation between Changsha and Sanya for yield trait in inbred parents and hybrids. (b) The proportion of residual variance of each yield related trait estimated in inbred parents and hybrid in rice. The significant test was obtained through the converted Z-score of the two correlation values (correlation coefficient between the phenotype of parent in two environment and the correlation coefficient between the phenotype of hybrid in two environment) and the number of samples (the number of samples used in parent and hybrid). One asterisk indicates the significance level of 0.05, two asterisks indicate the significance level of 0.01, and NS indicates that it is not statistically significant.

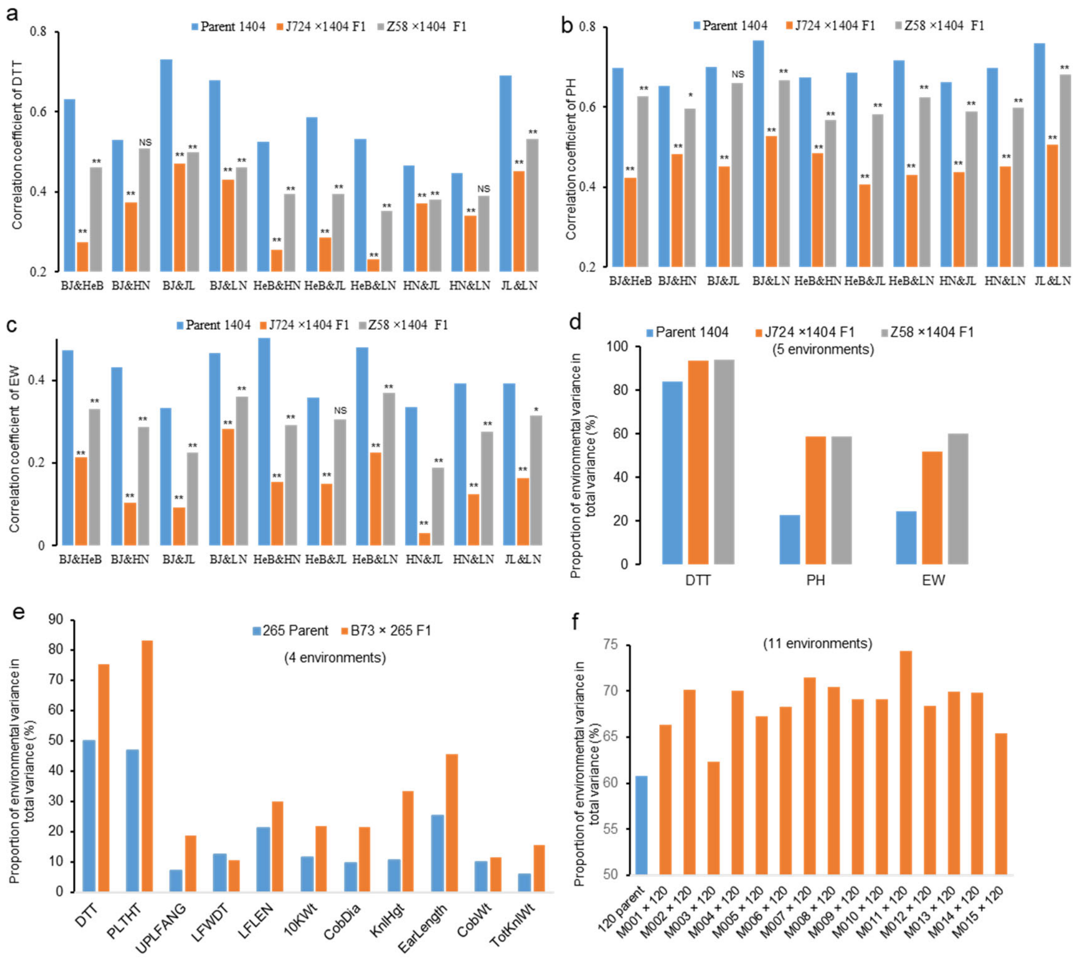


**Figure S5. The environmental effect on yield traits in inbred parents and hybrids.** (a-c) The phenotypic correlation between all possible combinations of two environment for the trait of DTT, PH and EW in inbred parents and hybrids. (The data were collected from Xiao. et. al. 2021). BJ, HeB, HN, JL and LN are the five grown locations of Beijing (40°10'N,116°21'E), Hebei province (38°39' N,115°51'E), Henan province (35°27'N,114°01'N) Jilin province (43°42'N,125°18'E) and Liaonin province (42°03'N, 123°33'E) in China; DTT, Days to anthesis; PH, Plant height; EW, ear Weight. (d) The proportion of environmental variance of each yield trait estimated in inbred parents and hybrid in maize for the trait of DTT, PH and EW across five environmental conditions. (e) The proportion of environmental variance of each yield trait estimated in inbred parents and hybrid in maize for 11 agronomic traits across four environmental conditions. (The data were collected from Sherry. et al. 2009). DTT, Days to anthesis; PLTHT, Plant height; UPLFANG, Upper leaf angle; LFWDT, Leaf width; LFLEN, leaf length; 10Kwt, 10 kernel weight; CobDia, cob diameter; KnHgt, kernel height; EarLength, Ear length; CobWt, Cob Weight; TolKnWt, Total Kernel Weight. (f) The proportion of environmental variance of grain yield estimated in inbred parents and hybrid in wheat across 11 environmental conditions. (The data were collected from Zhao. et al. 2015). The p value in (a), (b) and (c) was obtained through the converted Z-score, one asterisk indicates the significance level of 0.05, two asterisks indicate the significance level of 0.01, and NS indicates that it is not statistically significant.

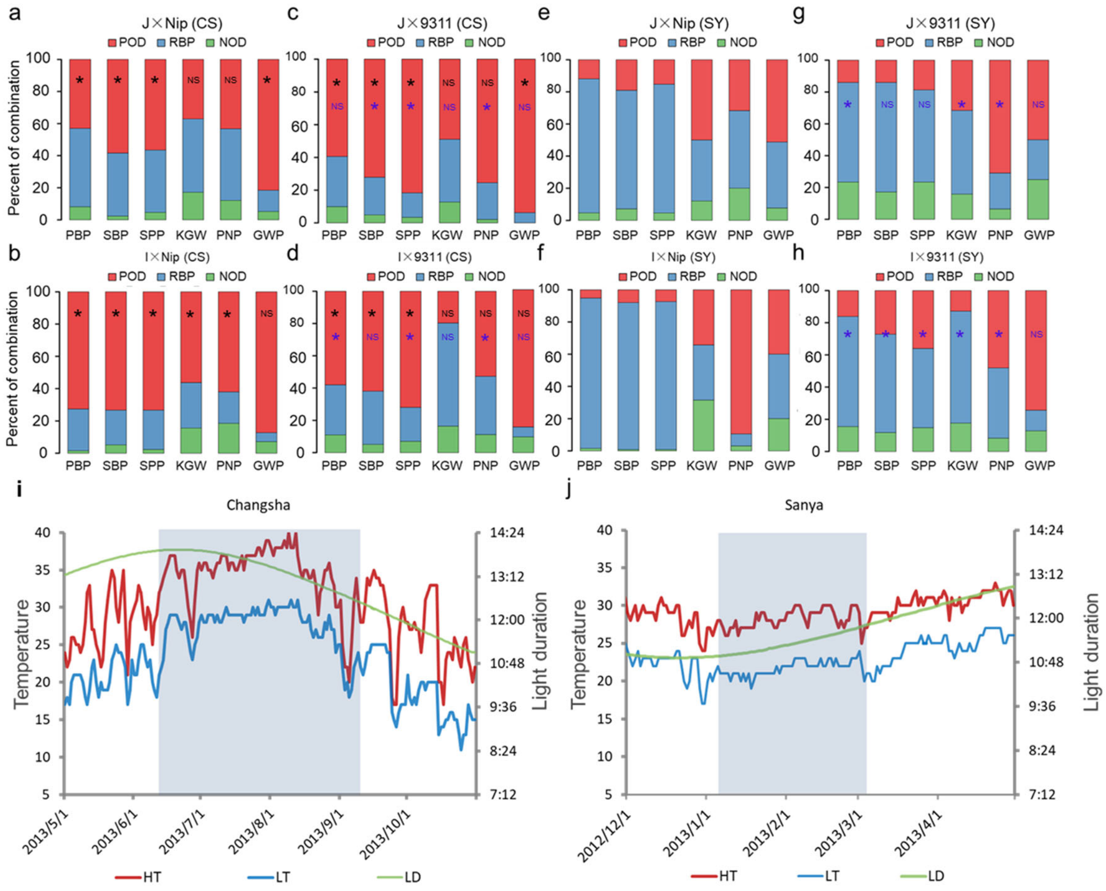

**Figure S6. The distribution of combinations showing POD, RBP and NOD phenotype for different yield traits and different kinds of combinations under two environments.** (a-d) The distribution in Changsha. (e-h) The distribution in Sanya. POD, $F_1$ showing phenotype over the higher parent, referred as positive overdominant (POD); RBP, $F_1$ showing phenotype ranging between parents referred as RBP; NOD, $F_1$ showing phenotype below the lower parent, referred as negative overdominant (NOD). The significance of the difference between the two environments was marked with black asterisk and the significance between inter and intrasubspecific combination was marked with bright blue asterisk (0.05 significant level). The specific comparisons between inter and intrasubspecific combinations were J×Nip vs J×9311 and I×Nip vs I×9311, and the level of statistical significance was derived from the Chi-square test. (i) The temperature and light duration during the growth season in Changsha. (j) The temperature and light duration during the growth season in Sanya. The grey boxes indicated the stage from reproductive initiation to grain filling. HT, high temperature; LT, low temperature; LD, light duration.

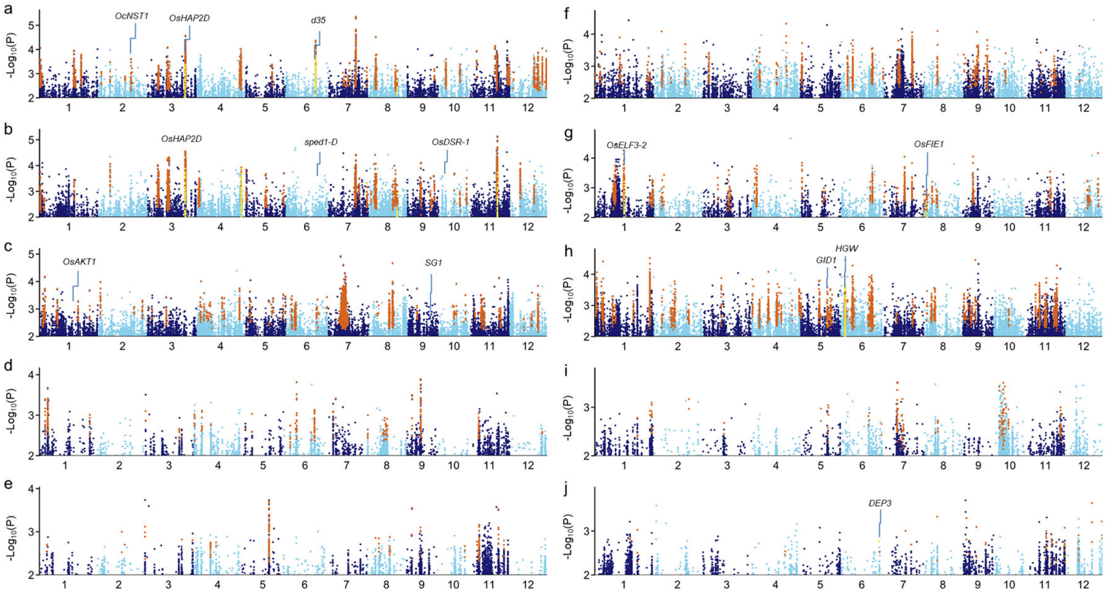


**Figure S7. Genome-wide association study of primary branch number per panicle (PBP) in *japonica* parents and their combinations using compressed MLM.** (a) Manhattan plots for J×Nip $F_1$ phenotype in Changsha. (b) Manhattan plots for J×Nip mid-parent heterosis value in Changsha. (c) Manhattan plots for phenotype of parents in Changsha. (d) Manhattan plots for J×9311 $F_1$ phenotype in Changsha. (e) Manhattan plots for J×9311 mid-parent heterosis value in Changsha. (f) Manhattan plots for J×Nip $F_1$ phenotype in Sanya. (g) Manhattan plots for J×Nip mid-parent heterosis value in Sanya. (h) Manhattan plots for phenotype of parents in Sanya. (i) Manhattan plots for J×9311 $F_1$ in Sanya. (j) Manhattan plots for J×9311 mid-parent heterosis in Sanya. Vermilion dots represent the significant SNPs in identified QTLs, yellow dots represent the SNPs in cloned genes with 2 kb promoter.

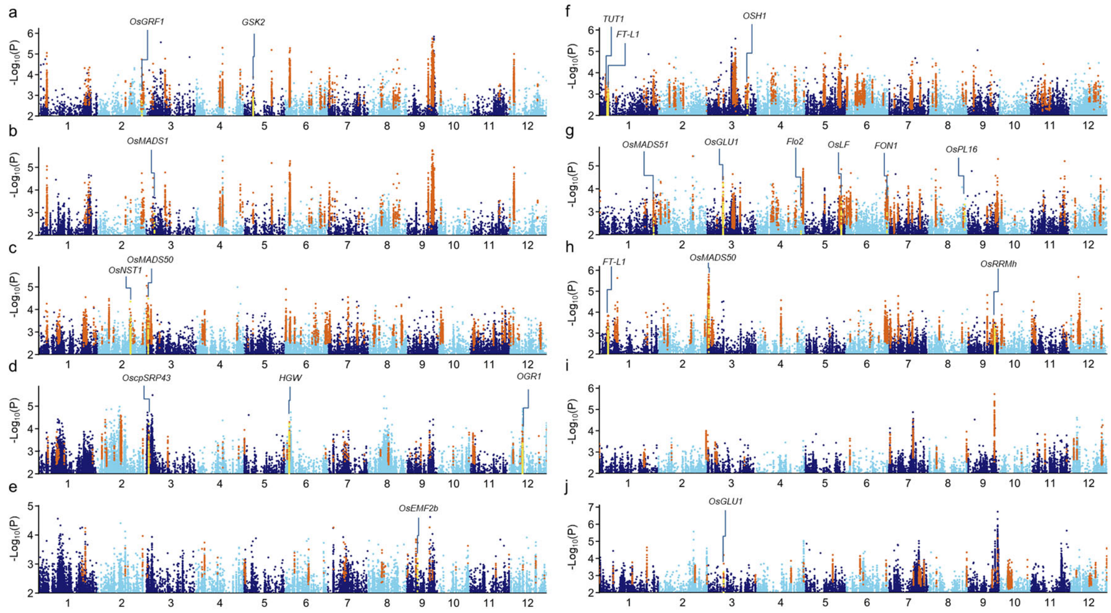


**Figure S8. Genome-wide association study of primary branch number per panicle (PBP) in *indica* parents and their combinations using compressed MLM.** (a) Manhattan plots for I×Nip $F_1$ phenotype in Changsha. (b) Manhattan plots for I×Nip mid-parent heterosis value in Changsha. (c) Manhattan plots for phenotype of parents in Changsha. (d) Manhattan plots for I×9311 $F_1$ phenotype in Changsha. (e) Manhattan plots for I×9311 mid-parent heterosis value in Changsha. (f) Manhattan plots for I×Nip $F_1$ phenotype in Sanya. (g) Manhattan plots for I×Nip mid-parent heterosis value in Sanya. (h) Manhattan plots for phenotype of parents in Sanya. (i) Manhattan plots for I×9311 $F_1$ in Sanya. (j) Manhattan plots for I×9311 mid-parent heterosis in Sanya. Vermilion dots represent the significant SNPs in identified QTLs, yellow dots represent the SNPs in cloned genes with 2 kb promoter.

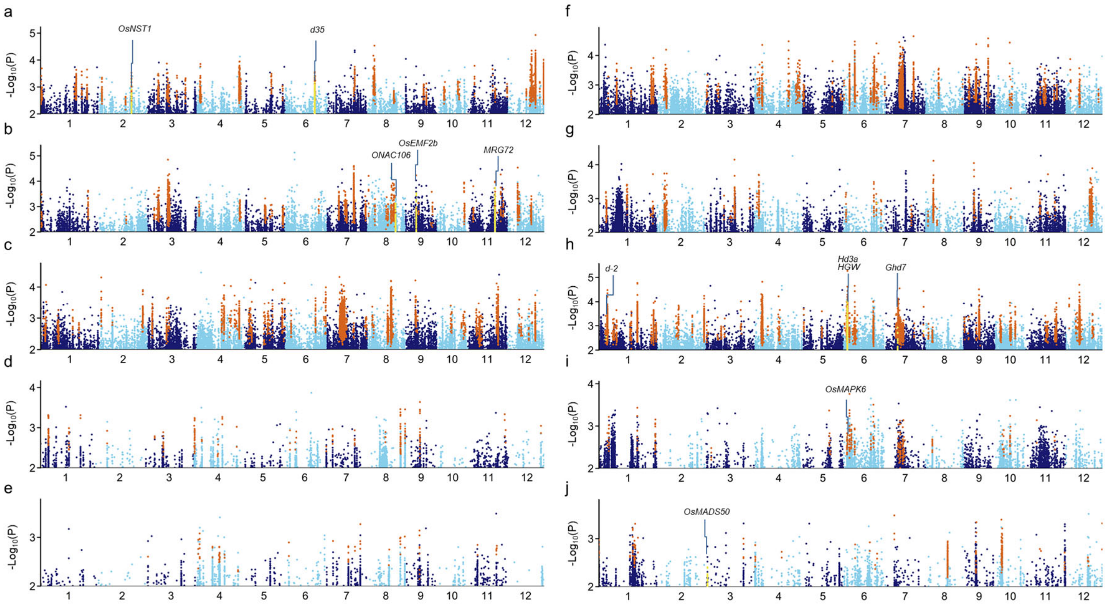


**Figure S9. Genome-wide association study of secondary branch number per panicle (SBP) in *japonica* parents and their combinations using compressed MLM.** (a) Manhattan plots for J×Nip $F_1$ phenotype in Changsha. (b) Manhattan plots for J×Nip mid-parent heterosis value in Changsha. (c) Manhattan plots for phenotype of parents in Changsha. (d) Manhattan plots for J×9311 $F_1$ phenotype in Changsha. (e) Manhattan plots for J×9311 mid-parent heterosis value in Changsha. (f) Manhattan plots for J×Nip $F_1$ phenotype in Sanya. (g) Manhattan plots for J×Nip mid-parent heterosis value in Sanya. (h) Manhattan plots for phenotype of parents in Sanya. (i) Manhattan plots for J×9311 $F_1$ in Sanya. (j) Manhattan plots for J×9311 mid-parent heterosis in Sanya. Vermilion dots represent the significant SNPs in identified QTLs, yellow dots represent the SNPs in cloned genes with 2 kb promoter.

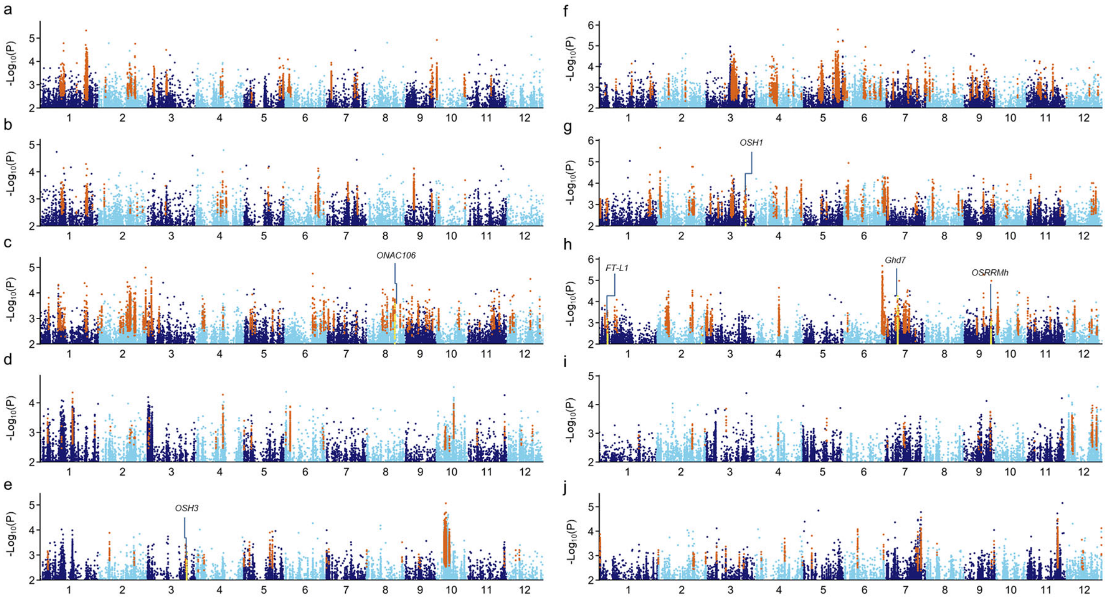


**Figure S10. Genome-wide association study of secondary branch number per panicle (SBP) in *indica* parents and their combinations using compressed MLM.** (a) Manhattan plots for I×Nip $F_1$ phenotype in Changsha. (b) Manhattan plots for I×Nip mid-parent heterosis value in Changsha. (c) Manhattan plots for phenotype of parents in Changsha. (d) Manhattan plots for I×9311 $F_1$ phenotype in Changsha. (e) Manhattan plots for I×9311 mid-parent heterosis value in Changsha. (f) Manhattan plots for I×Nip $F_1$ phenotype in Sanya. (g) Manhattan plots for I×Nip mid-parent heterosis value in Sanya. (h) Manhattan plots for phenotype of parents in Sanya. (i) Manhattan plots for I×9311 $F_1$ in Sanya. (j) Manhattan plots for I×9311 mid-parent heterosis in Sanya. Vermilion dots represent the significant SNPs in identified QTLs, yellow dots represent the SNPs in cloned genes with 2 kb promoter.

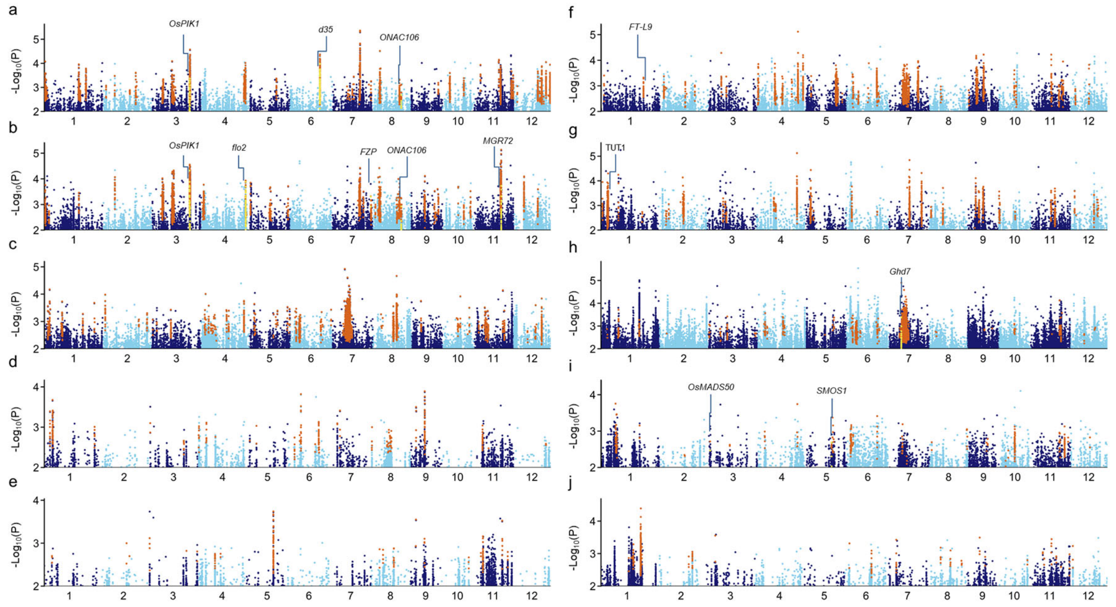


**Figure S11. Genome-wide association study of spikelet number per panicle (SPP) in *japonica* parents and their combinations using compressed MLM.** (a) Manhattan plots for J×Nip $F_1$ phenotype in Changsha. (b) Manhattan plots for J×Nip mid-parent heterosis value in Changsha. (c) Manhattan plots for phenotype of parents in Changsha. (d) Manhattan plots for J×9311 $F_1$ phenotype in Changsha. (e) Manhattan plots for J×9311 mid-parent heterosis value in Changsha. (f) Manhattan plots for J×Nip $F_1$ phenotype in Sanya. (g) Manhattan plots for J×Nip mid-parent heterosis value in Sanya. (h) Manhattan plots for phenotype of parents in Sanya. (i) Manhattan plots for J×9311 $F_1$ in Sanya. (j) Manhattan plots for J×9311 mid-parent heterosis in Sanya. Vermilion dots represent the significant SNPs in identified QTLs, yellow dots represent the SNPs in cloned genes with 2 kb promoter.

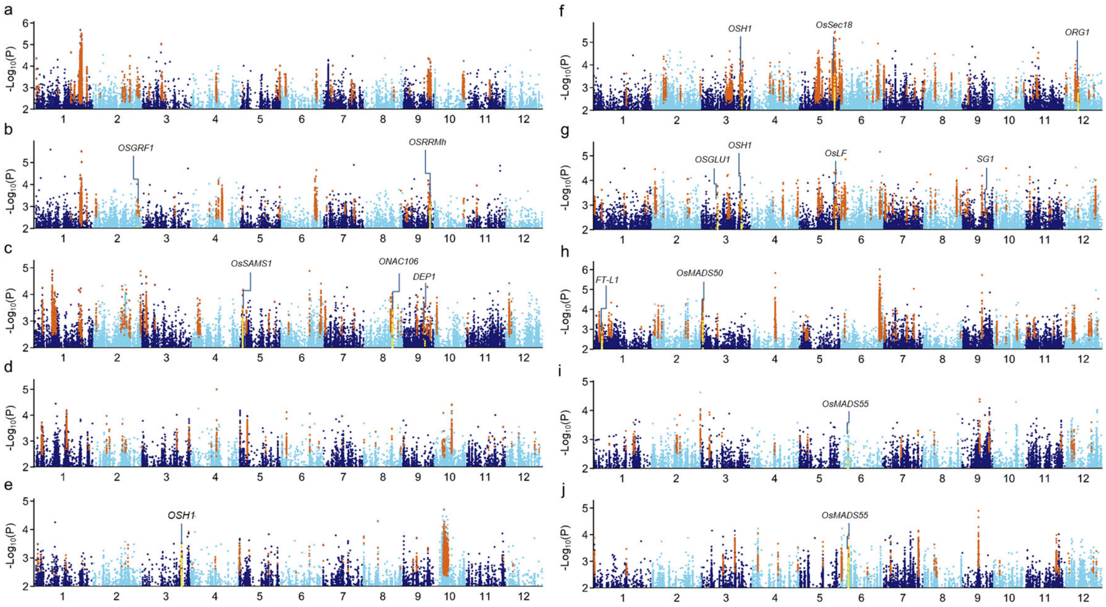


**Figure S12. Genome-wide association studies of spikelet number per panicle (SPP) in *indica* parents and their combinations using compressed MLM.** (a) Manhattan plots for I×Nip $F_1$ phenotype in Changsha. (b) Manhattan plots for I×Nip mid-parent heterosis value in Changsha. (c) Manhattan plots for phenotype of parents in Changsha. (d) Manhattan plots for I×9311 $F_1$ phenotype in Changsha. (e) Manhattan plots for I×9311 mid-parent heterosis value in Changsha. (f) Manhattan plots for I×Nip $F_1$ phenotype in Sanya. (g) Manhattan plots for I×Nip mid-parent heterosis value in Sanya. (h) Manhattan plots for phenotype of parents in Sanya. (i) Manhattan plots for I×9311 $F_1$ in Sanya. (j) Manhattan plots for I×9311 mid-parent heterosis in Sanya. Vermilion dots represent the significant SNPs in identified QTLs, yellow dots represent the SNPs in cloned genes with 2 kb promoter.

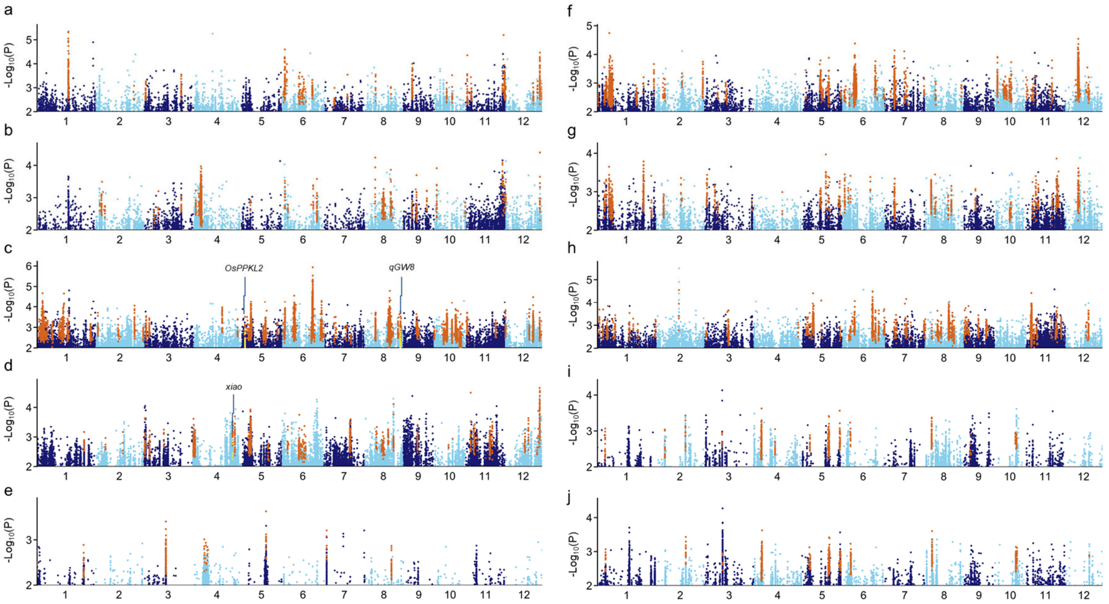


**Figure S13. Genome-wide association study of 1000-grain weight (KGW) in *japonica* parents and their combinations using compressed MLM.** (a) Manhattan plots for J×Nip $F_1$ phenotype in Changsha. (b) Manhattan plots for J×Nip mid-parent heterosis value in Changsha. (c) Manhattan plots for phenotype of parents in Changsha. (d) Manhattan plots for J×9311 $F_1$ phenotype in Changsha. (e) Manhattan plots for J×9311 mid-parent heterosis value in Changsha. (f) Manhattan plots for J×Nip $F_1$ phenotype in Sanya. (g) Manhattan plots for J×Nip mid-parent heterosis value in Sanya. (h) Manhattan plots for phenotype of parents in Sanya. (i) Manhattan plots for J×9311 $F_1$ in Sanya. (j) Manhattan plots for J×9311 mid-parent heterosis in Sanya. Vermilion dots represent the significant SNPs in identified QTLs, yellow dots represent the SNPs in cloned genes with 2 kb promoter.

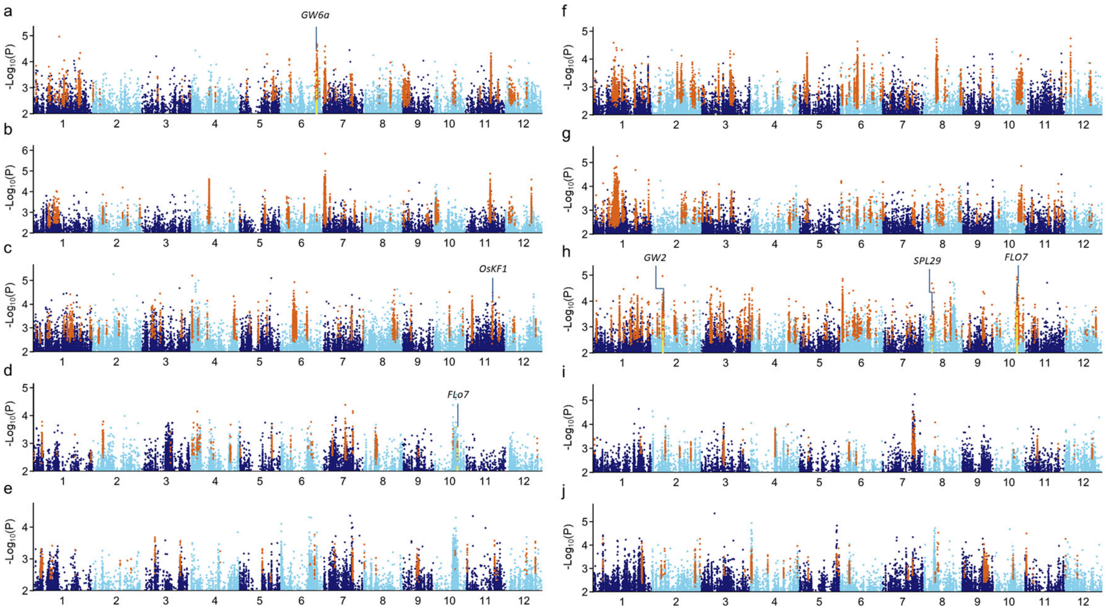


**Figure S14. Genome-wide association study of 1000-grain weight (KGW) in *indica* parents and their combinations using compressed MLM.** (a) Manhattan plots for I×Nip $F_1$ phenotype in Changsha. (b) Manhattan plots for I×Nip mid-parent heterosis value in Changsha. (c) Manhattan plots for phenotype of parents in Changsha. (d) Manhattan plots for I×9311 $F_1$ phenotype in Changsha. (e) Manhattan plots for I×9311 mid-parent heterosis value in Changsha. (f) Manhattan plots for I×Nip $F_1$ phenotype in Sanya. (g) Manhattan plots for I×Nip mid-parent heterosis value in Sanya. (h) Manhattan plots for phenotype of parents in Sanya. (i) Manhattan plots for I×9311 $F_1$ in Sanya. (j) Manhattan plots for I×9311 mid-parent heterosis in Sanya. Vermilion dots represent the significant SNPs in identified QTLs, yellow dots represent the SNPs in cloned genes with 2 kb promoter.

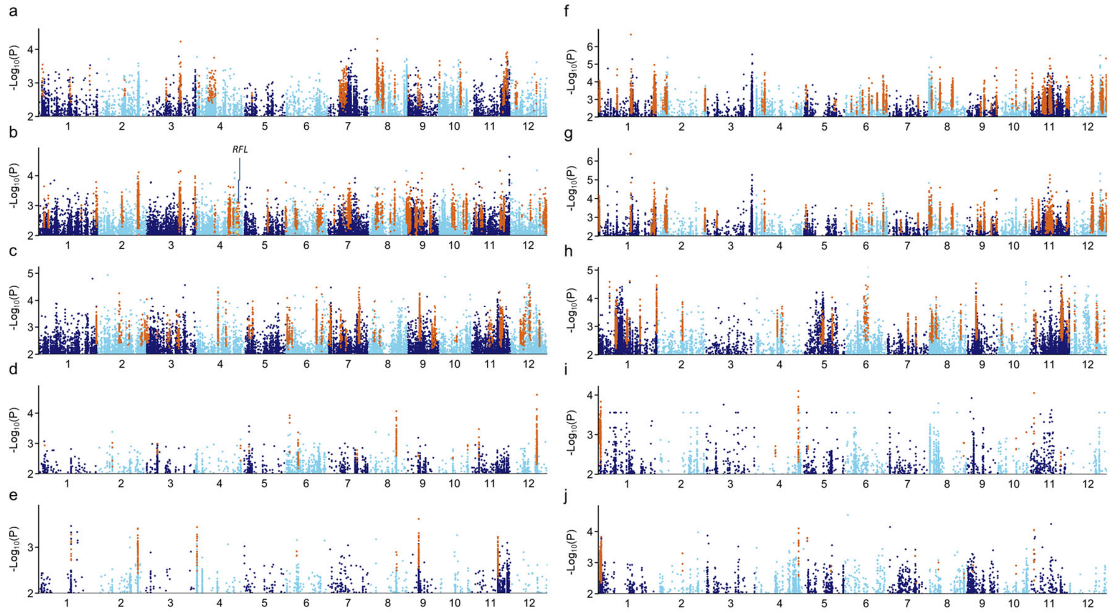


**Figure S15. Genome-wide association study of panicle number per plant (PNP) in *japonica* parents and their combinations using compressed MLM.** (a) Manhattan plots for J×Nip $F_1$ phenotype in Changsha. (b) Manhattan plots for J×Nip mid-parent heterosis value in Changsha. (c) Manhattan plots for phenotype of parents in Changsha. (d) Manhattan plots for J×9311 $F_1$ phenotype in Changsha. (e) Manhattan plots for J×9311 mid-parent heterosis value in Changsha. (f) Manhattan plots for J×Nip $F_1$ phenotype in Sanya. (g) Manhattan plots for J×Nip mid-parent heterosis value in Sanya. (h) Manhattan plots for phenotype of parents in Sanya. (i) Manhattan plots for J×9311 $F_1$ in Sanya. (j) Manhattan plots for J×9311 mid-parent heterosis in Sanya. Vermilion dots represent the significant SNPs in identified QTLs, yellow dots represent the SNPs in cloned genes with 2 kb promoter.

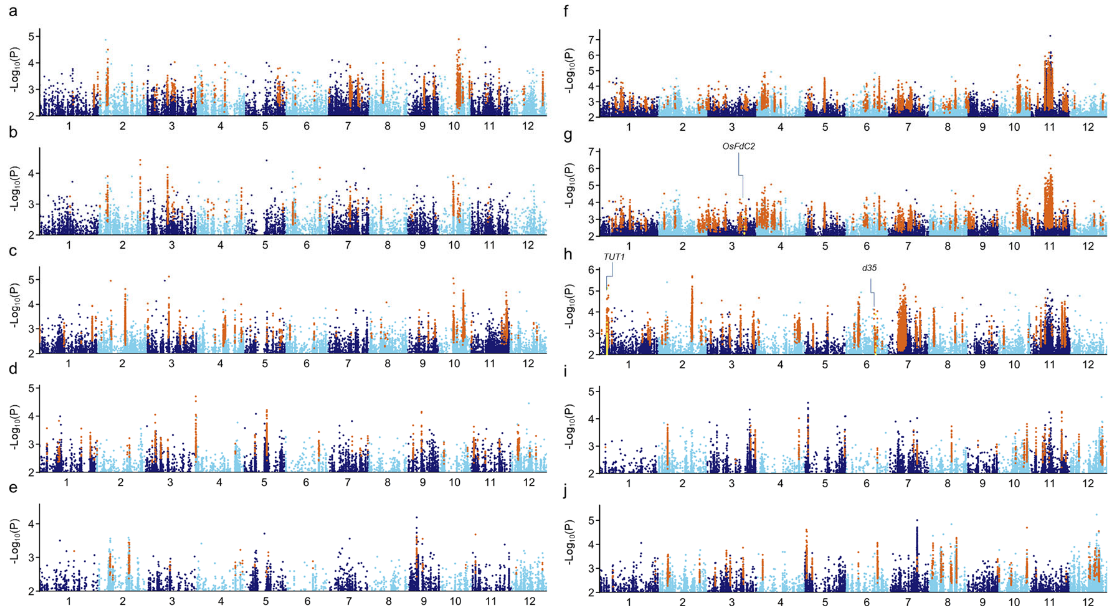


**Figure S16. Genome-wide association study of panicle number per plant (PNP) in *indica* parents and their combinations using compressed MLM.** (a) Manhattan plots for I×Nip $F_1$ phenotype in Changsha. (b) Manhattan plots for I×Nip mid-parent heterosis value in Changsha. (c) Manhattan plots for phenotype of parents in Changsha. (d) Manhattan plots for I×9311 $F_1$ phenotype in Changsha. (e) Manhattan plots for I×9311 mid-parent heterosis value in Changsha. (f) Manhattan plots for I×Nip $F_1$ phenotype in Sanya. (g) Manhattan plots for I×Nip mid-parent heterosis value in Sanya. (h) Manhattan plots for phenotype of parents in Sanya. (i) Manhattan plots for I×9311 $F_1$ in Sanya. (j) Manhattan plots for I×9311 mid-parent heterosis in Sanya. Vermilion dots represent the significant SNPs in identified QTLs, yellow dots represent the SNPs in cloned genes with 2 kb promoter.

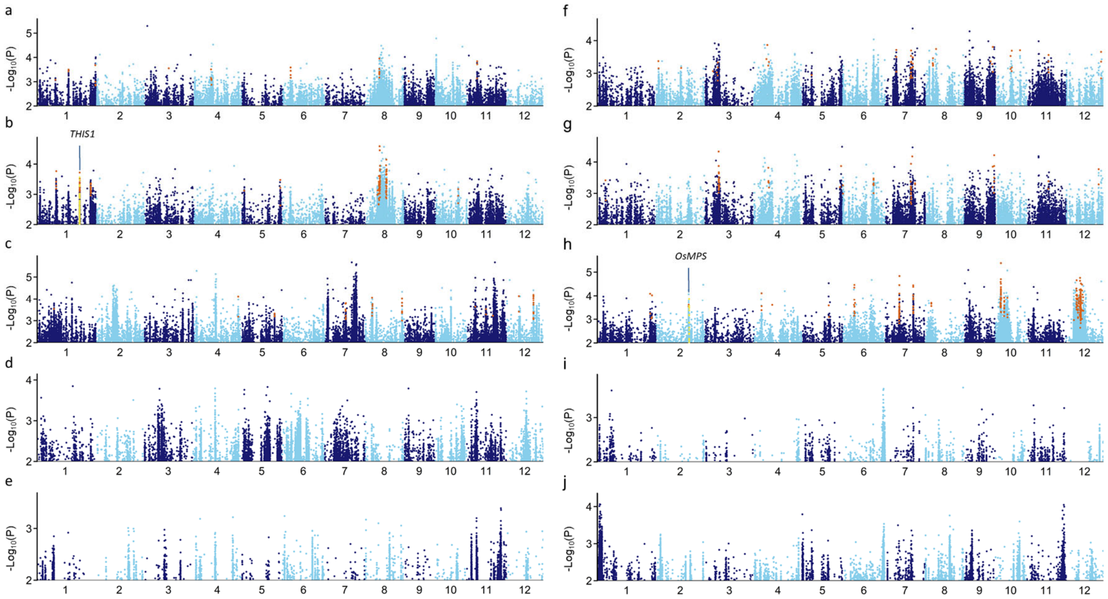


**Figure S17. Genome-wide association study of grain weight per plant (GWP) in *japonica* parents and their combinations using compressed MLM.** (a) Manhattan plots for J×Nip $F_1$ phenotype in Changsha. (b) Manhattan plots for J×Nip mid-parent heterosis value in Changsha. (c) Manhattan plots for phenotype of parents in Changsha. (d) Manhattan plots for J×9311 $F_1$ phenotype in Changsha. (e) Manhattan plots for J×9311 mid-parent heterosis value in Changsha. (f) Manhattan plots for J×Nip $F_1$ phenotype in Sanya. (g) Manhattan plots for J×Nip mid-parent heterosis value in Sanya. (h) Manhattan plots for phenotype of parents in Sanya. (i) Manhattan plots for J×9311 $F_1$ in Sanya. (j) Manhattan plots for J×9311 mid-parent heterosis in Sanya. Vermilion dots represent the significant SNPs in identified QTLs, yellow dots represent the SNPs in cloned genes with 2 kb promoter.

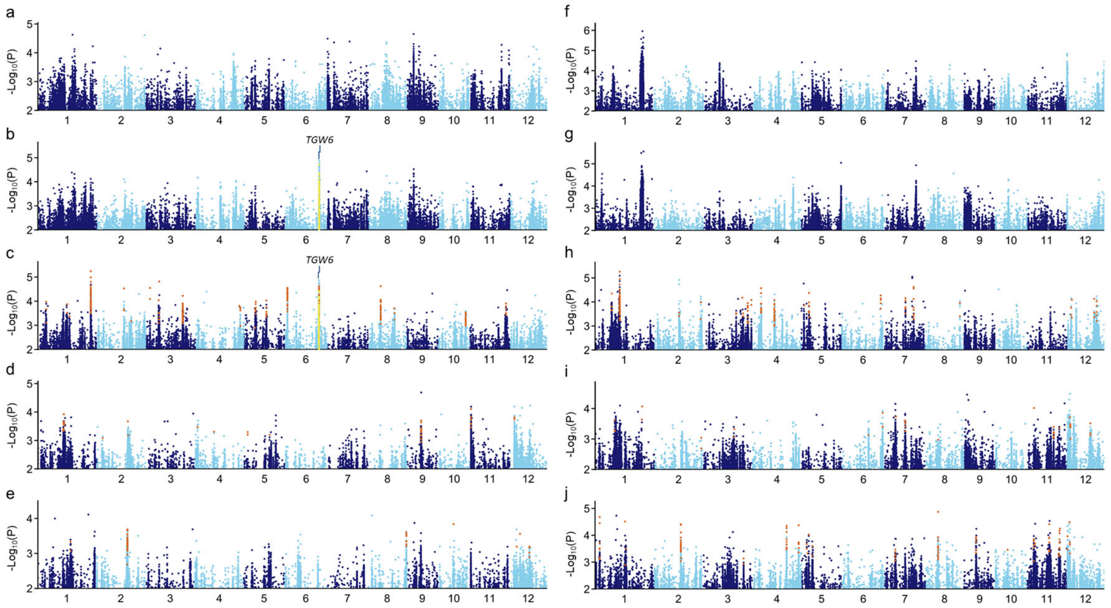


**Figure S18. Genome-wide association study of grain weight per plant (GWP) in *indica* parents and their combinations using compressed MLM.** (a) Manhattan plots for I×Nip $F_1$ phenotype in Changsha. (b) Manhattan plots for I×Nip mid-parent heterosis value in Changsha. (c) Manhattan plots for phenotype of parents in Changsha. (d) Manhattan plots for I×9311 $F_1$ phenotype in Changsha. (e) Manhattan plots for I×9311 mid-parent heterosis value in Changsha. (f) Manhattan plots for I×Nip $F_1$ phenotype in Sanya. (g) Manhattan plots for I×Nip mid-parent heterosis value in Sanya. (h) Manhattan plots for phenotype of parents in Sanya. (i) Manhattan plots for I×9311 $F_1$ in Sanya. (j) Manhattan plots for I×9311 mid-parent heterosis in Sanya. Vermilion dots represent the significant SNPs in identified QTLs, yellow dots represent the SNPs in cloned genes with 2 kb promoter.

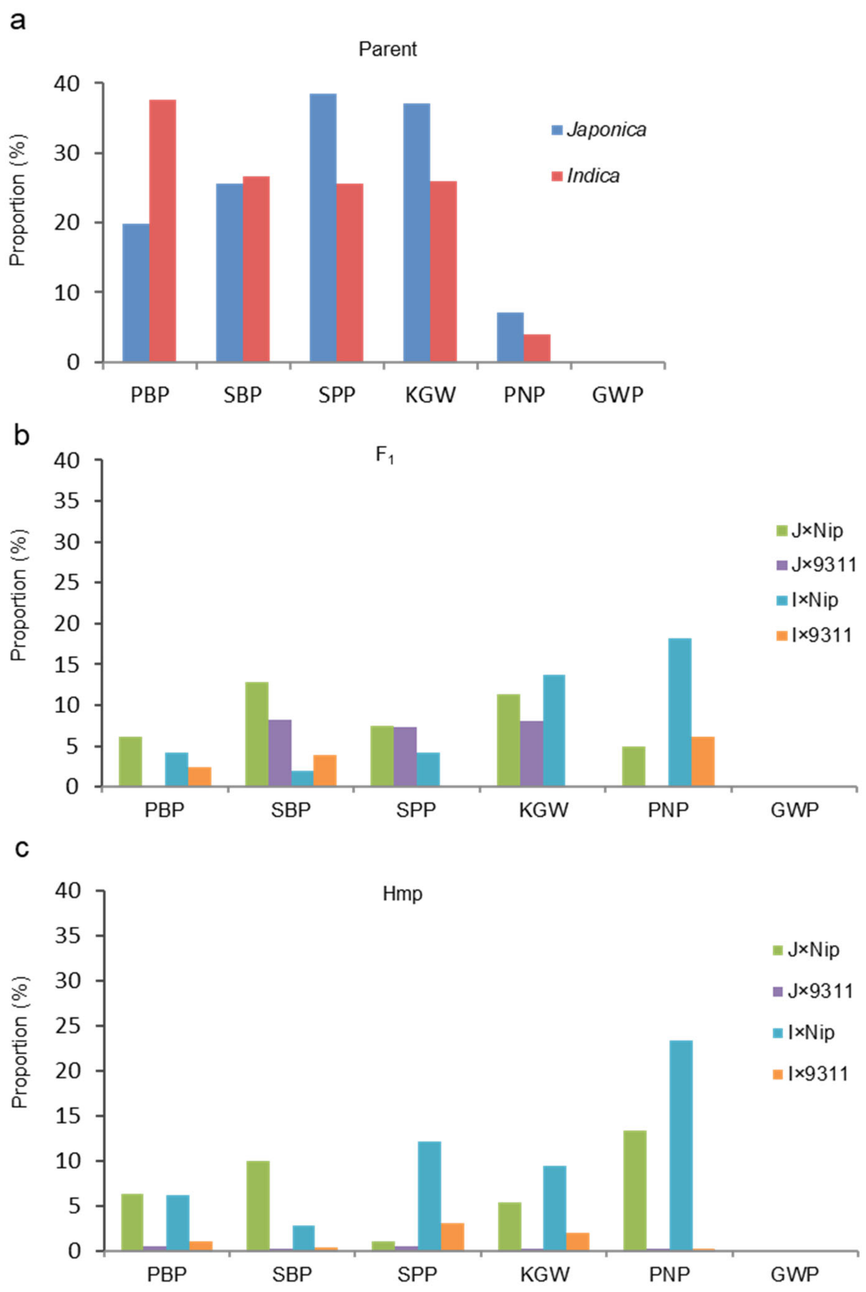


**Figure S19. The proportion of colocalized QTL between two environments for yield and its sub-component traits.** (a) The proportion of colocalized QTL between Changsha and Sanya for P_QTL. (b) The proportion of colocalized QTL between Changsha and Sanya for $F_1$ _QTL. (c) The proportion of colocalized QTL between Changsha and Sanya for Hmp_QTL.

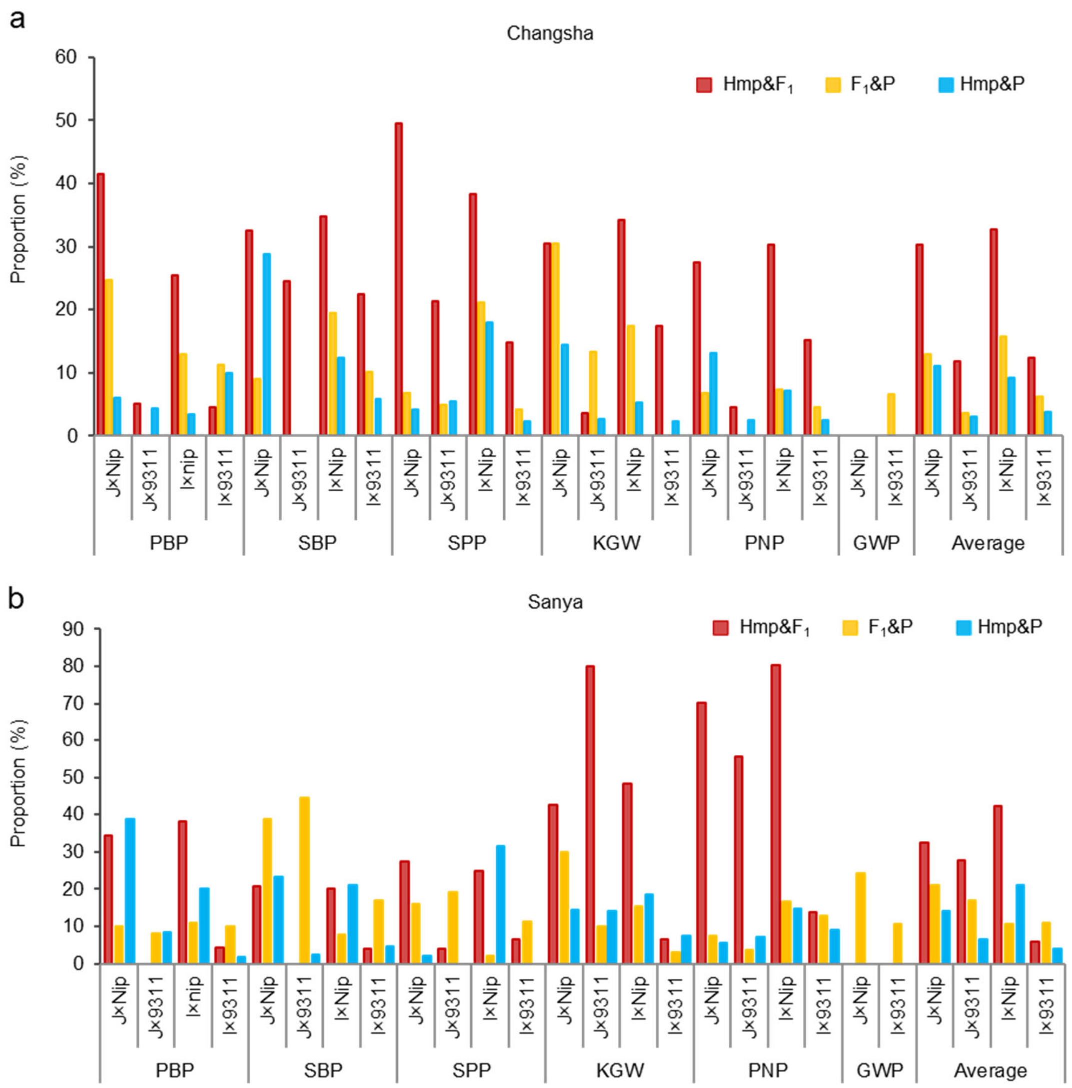


**Figure S20. The proportion of colocalized QTL between F$_1$_QTL, Hmp_QTL and P _QTL for grain yield and its sub-component traits.** (a) The proportion of colocalized QTL between F$_1$_QTL, Hmp_QTL and P_QTL for the trait of PBP, SBP, SPP, KGW, PNP and GWP in Changsha. (b) The proportion of colocalized QTLs between F$_1$_QTL, Hmp_QTL and P_QTL for the trait of PBP, SBP, SPP, KGW, PNP and GWP in Sanya.

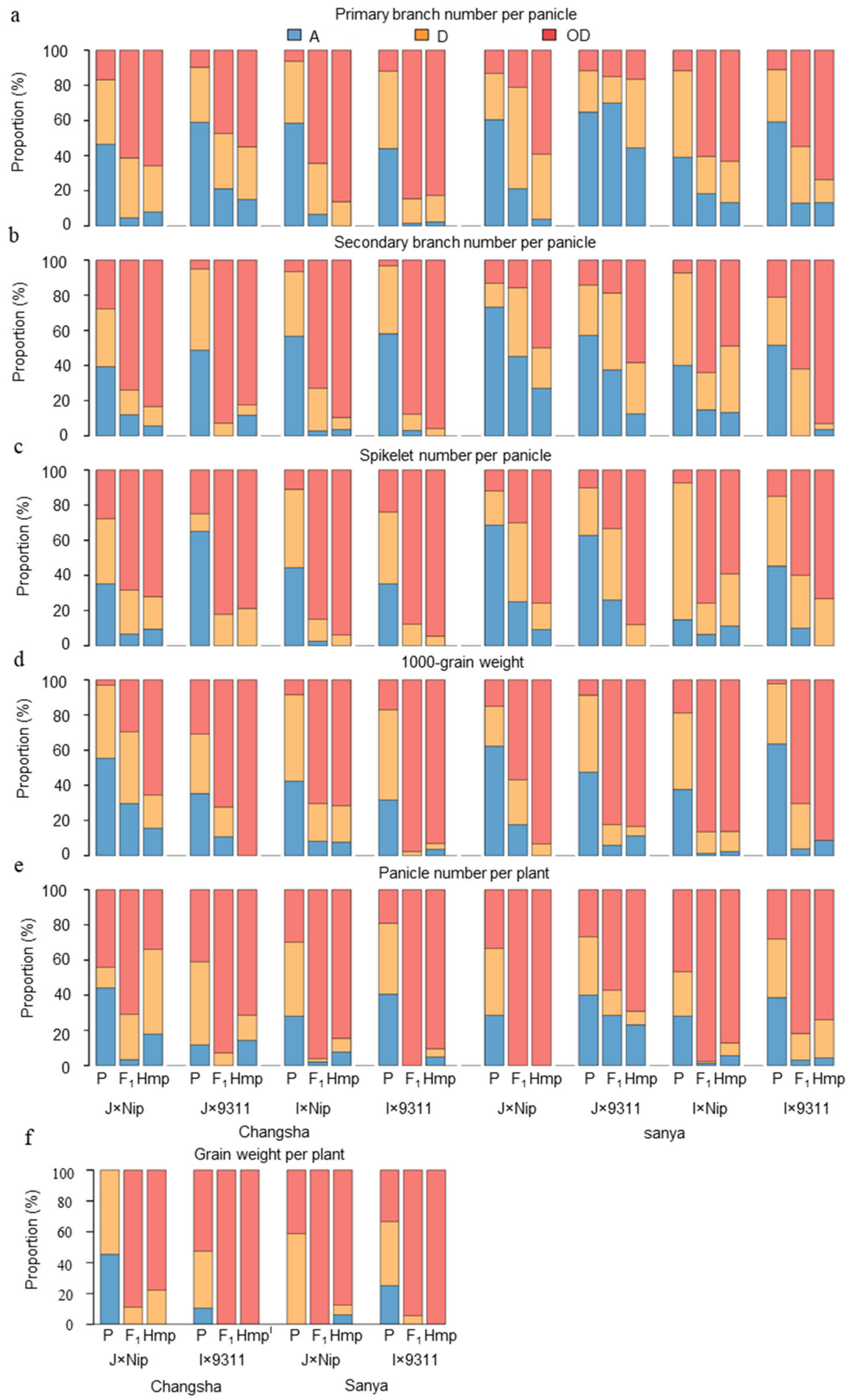


**Figure S21. The proportion of different types of QTLs identified in yield related trait using the phenotype of parents (P), the phenotype of $F_1$ ($F_1$) and the middle parent heterosis value (Hmp).** A, additive (in blue); D, dominant (in orange); OD, overdominant (in red).

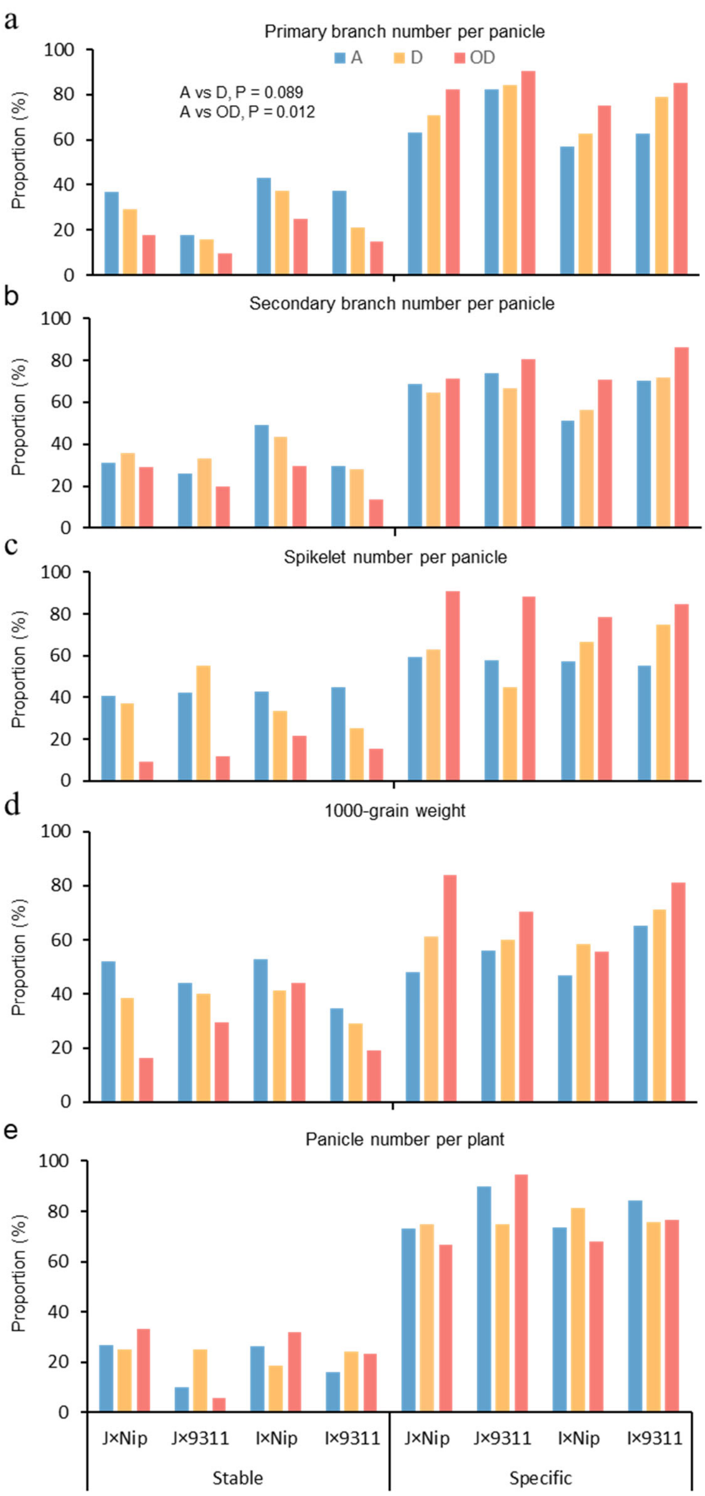


**Fig. S22. The proportion of additive, dominant and overdominant preferred QTLs colocalized between two environments.** Stable, means the colocalized QTLs between two environments; Specific, means the QTLs can only be detected in one environment.

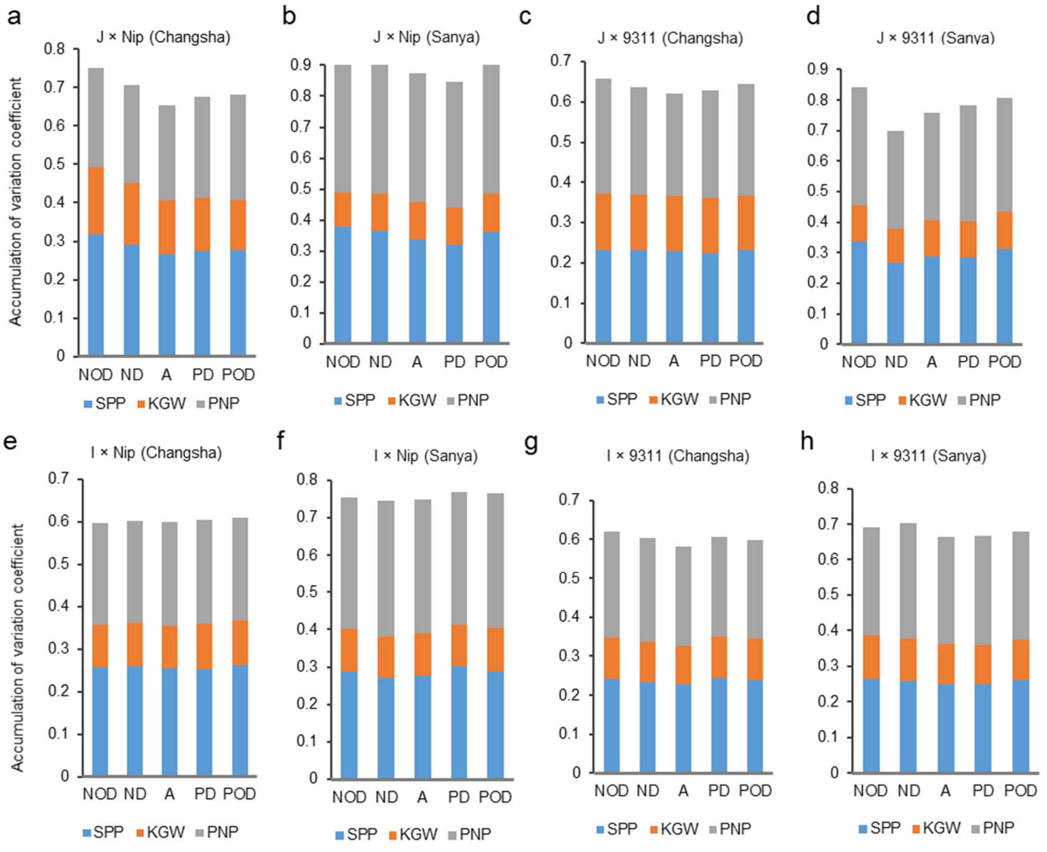


**Figure S23. The accumulation of average coefficient of variation estimated in each identified QTL across four kinds of combination for different type of genetic component.** Here NOD, ND, A, PD and POD represent the type of negative overdominant, negative dominant, additive, positive dominant and positive overdominant QTLs.

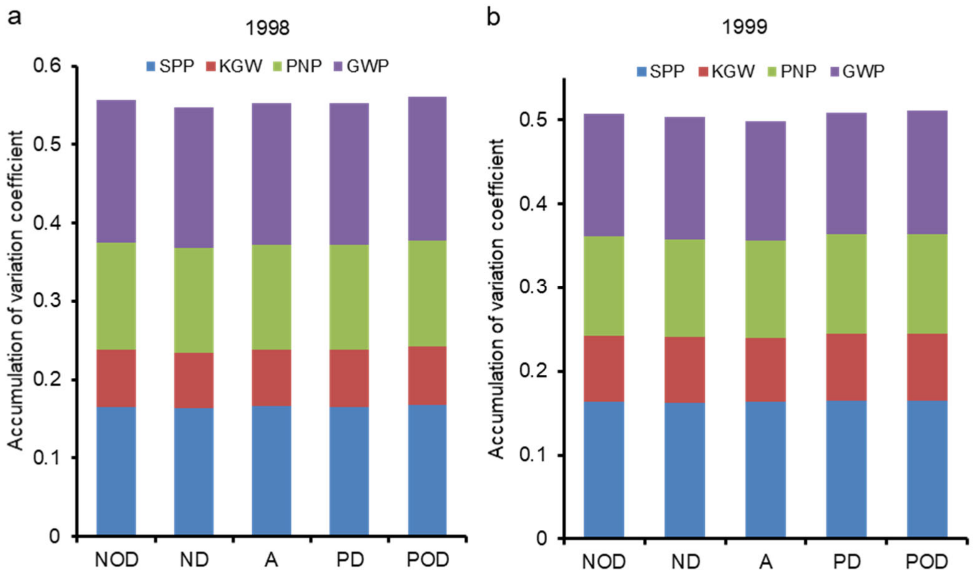


**Figure S24. The accumulation of average coefficient of variation estimated in each identified QTL for different type genetic component in IMF$_2$ population.** (a) The comparisons of additive, dominance and overdominance for the accumulation of average variation coefficient estimated in each identified QTL at 1998. (b) The comparisons of additive, dominance and overdominance for the accumulation of average variation coefficient estimated in each identified QTL at 1999.

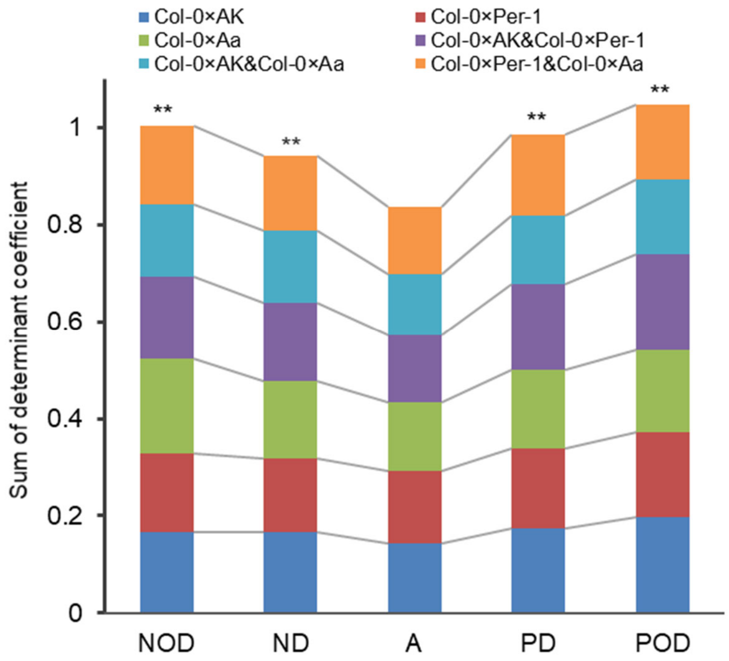


**Figure S25. The Sum of average determinant coefficient between genes with different expression patterns and their transcription factors in three *A. thaliana* combinations.** The determination coefficient was estimated by six pairs of transcription levels in the first leaf between the gene and its transcription factor across three *A. thaliana* combinations (including Col-0×Per-1, Col-0×Aa, Col-0×Ak) and their parents. Here, Col-0×AK& Col-0×Per-1 refers to those genes that show the same expression pattern in the combination of Col-0×AK and Col-0×Per-1. And the others are similar. We estimated the significant difference of NOD, ND, PD and POD with A by two-tailed scaled paired *T*-test ; and "**" marked the significant level at 0.01. The raw sequence data were collected from the published paper (Yang, M. et al. Genomic architecture of biomass heterosis. Proc Natl Acad Sci USA. 2017, 114:8101-8106).

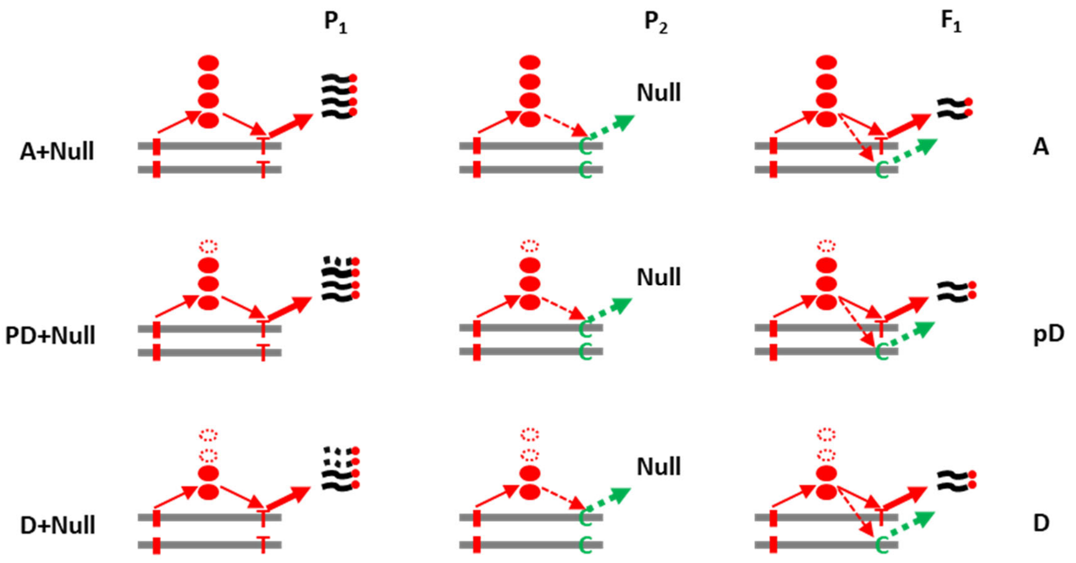


**Figure S26. The schematic diagram of regulation model for the molecular mechanism of additive and dominant effect produced by single site with null allele (C) and one functional allele (T) under one positive regulator background**. The grey thick lines show two chromosomes, the red bars on which are two homologous alleles of the regulator that are uniform among $P_1$, $P_2$ and $F_1$. The break and solid pies together represent the required regulator function that can maximize the function of T/T homozygote of the target site, and the solid pies represent different regulator functions and thus provide different backgrounds to the target site. The arrow represents the function process, and the break arrow represents the break function process. The break and solid curves together represent the maximum function of the T/T homozygote in parents or one T allele in $F_1$, and the solid curves represent the real function. A+Null shows the action mode for each allele and between two alleles (T vs C) that they are independent with T being additive and C being null; and PD+Null and D+Null are similar. A, pD and D mean additive, partial dominant, and dominant effect, respectively.

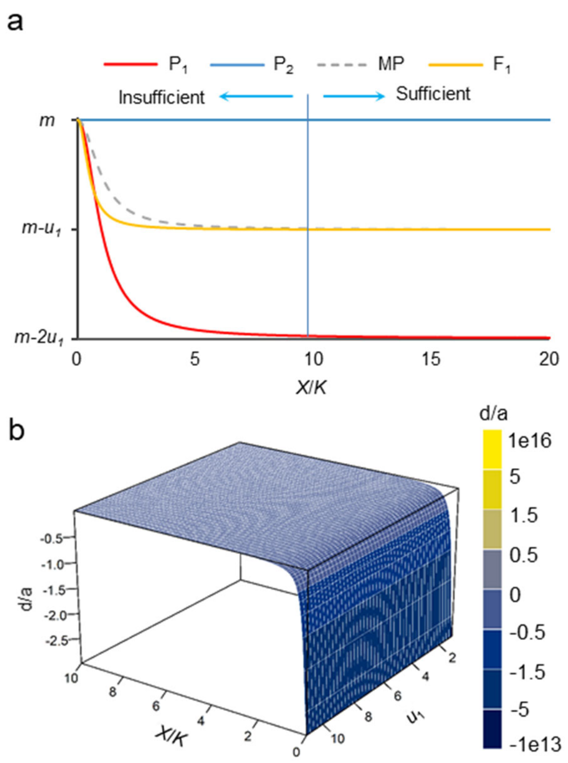


**Figure S27. The simulated diagram of regulation model for molecular mechanism of additive and dominant effect produced by single site with null allele and one functional allele under one regulator background**. (a) The performance of the target site in two parents, $F_1$ and the middle parent (MP) under the repressor background with different sufficiencies (*X/K*). It was simulated according to Hill function with $\mu_1 = 3$ and $n = 2$. $\mu_1$ means the maximum function at steady state for one functional allele. $n$ is the Hill coefficient. Left arrow represents a relatively insufficient activator background, and the right arrow represents the relatively sufficient activator background. (b) The dominant degree of the target site under the repressor background with different sufficiencies (*X/K*) and different $\mu_1$. d/a means the degree of dominance to additive effect ratio.

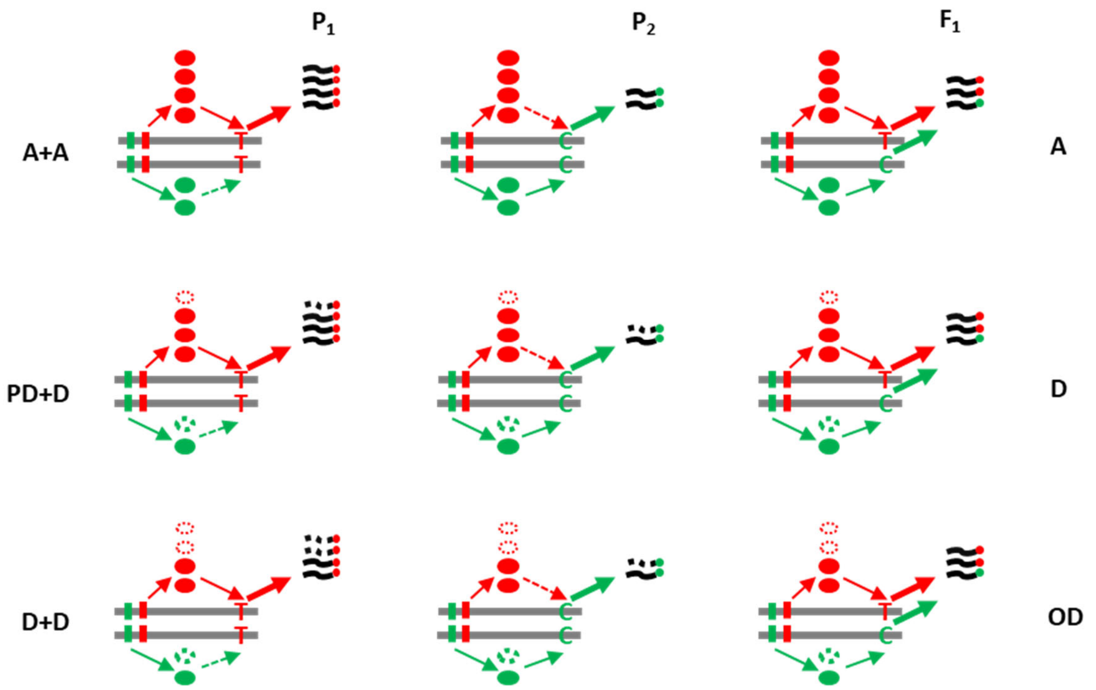


**Figure S28. The schematic diagram of regulation model for molecular mechanism of additive, dominant and overdominant effect produced by the cumulated functions of two alleles of one polymorphic site under two independent positive regulators as the upstream backgrounds**. The grey thick lines show two chromosomes, the red and green bars on which represent the homologous alleles of the regulators of T and C alleles at target site, respectively; and the function of these regulators keep consistent and among $P_1$, $P_2$ and $F_1$. The break and solid pies together represent the required regulator function that can maximize the function of the homozygote of the corresponding target allele, and the solid pies represent different regulator functions and thus provide different backgrounds to the target allele. The break and solid arrows represents sthat the target allele (T or C) can be regulated and cannot be regulated, respectively. The break and solid curves together represent the maximum function of the homozygote in parents or one allele in $F_1$, and the solid curves represent the real function; those curves with red or green dots represent the function of allele T or C respectively. A+A shows the action mode for each allele and between two alleles (T vs C) that they are independent and cumulative with T and C both being additive; and PD+D and D+D are similar. A, D and OD mean additive, dominant, and overdominant effect, respectively.

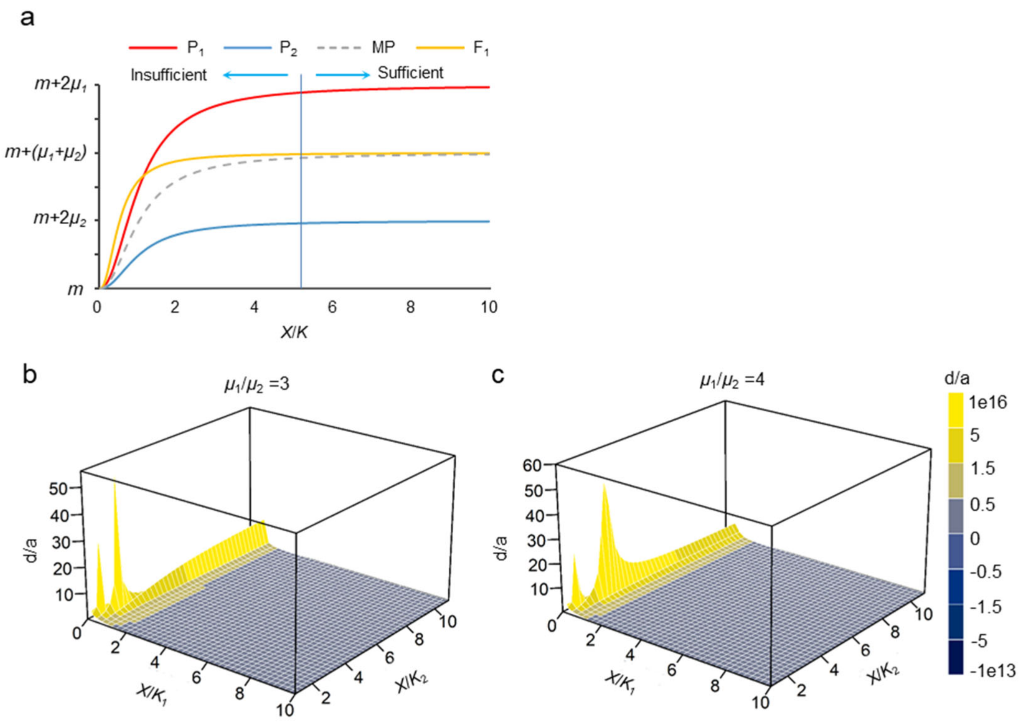


**Figure S29. The simulated diagram of regulation model for molecular mechanism of additive, dominant and overdominant effect produced by the cumulated functions of two alleles of one polymorphic site under two independent positive regulators or responsors as the backgrounds.** (a) The simulated performance of two parent, $F_1$ and middle parent (MP) under the condition of $\mu_1$ =3, $\mu_2$ =1 and $K_1$ = $K_2$ for positive regulation. left arrow means background is relative insufficient and right arrow means background is relative sufficient. (b-c) The simulated distribution of d/a with same homologous backgrounds, but the two alleles in $F_1$ are regulated by different factors in the background. d/a means the degree of dominance to additive effect ratio , it can be in both positive and negative direction. $\mu_1/\mu_2$ means the ratio of maximum function of $P_1$ genotype to maximum function of $P_2$ genotype when their respective background can afford to the complete expression of the corresponding homologous genotype.

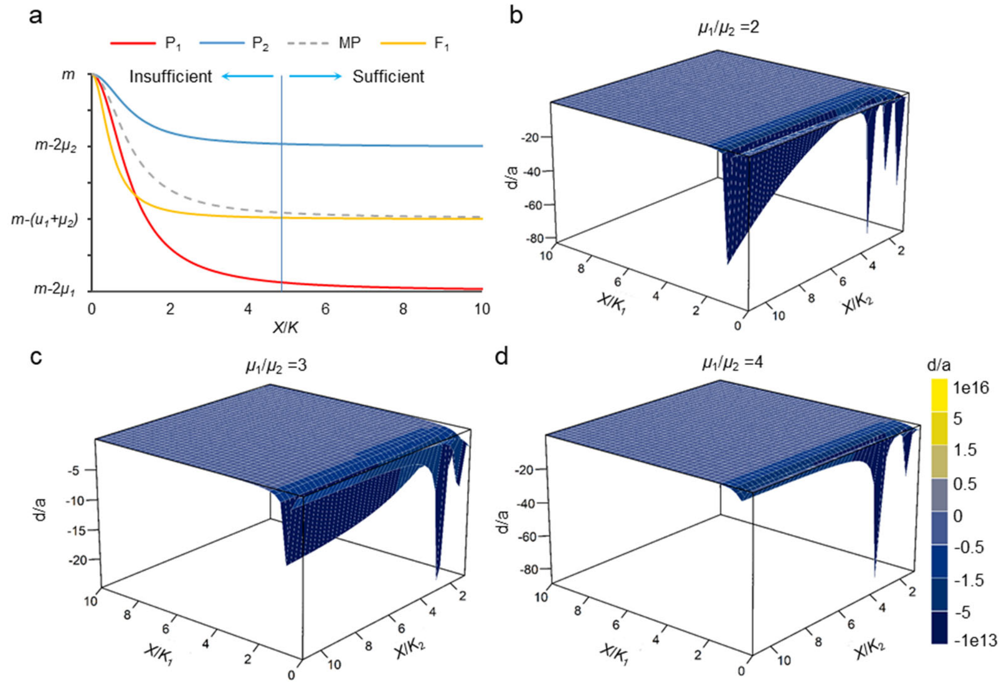


**Figure S30. The simulated diagram of regulation model for molecular mechanism of additive, dominant and overdominant effect produced by the cumulated functions of two alleles of one polymorphic site under two independent negative regulators or responsors as the backgrounds**. (a) The performance of the target site in two parents, $F_1$ and the middle parent (MP) under the repressor background with different sufficiencies (*X/K*). It was simulated according to Hill function with $\mu_1 = 3$, $\mu_2 = 1$, $K_1 = K_2$ and $n = 2$. $\mu_1$ and $\mu_2$ means the maximum function at steady state for allele with higher function and lower function, respectively. $n$ is the Hill coefficient. Left arrow represents a relatively insufficient repressor background, and the right arrow represents the relatively sufficient repressor background. (b) The dominant degree of the target site under the repressor background with different sufficiencies (X/K) for two alleles of the target site with $\mu_1/\mu_2 = 2$. (c) The dominant degree of the target site under the repressor background with different sufficiencies (X/K) for two alleles of the target site with $\mu_1/\mu_2 = 3$. (d) The dominant degree of the target site under the repressor background with different sufficiencies (*X/K*) for two alleles of the target site with $\mu_1/\mu_2 = 4$.

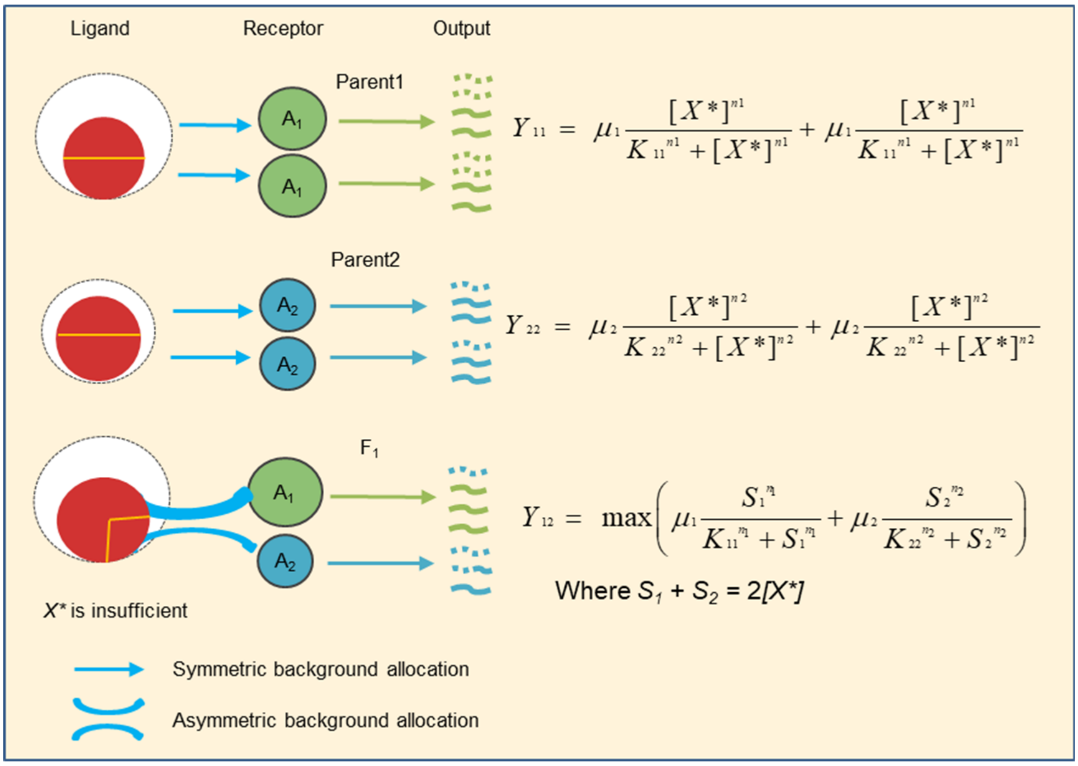


**Figure S31. The regulation model for molecular mechanism of dominant and overdominant effect in heterozygote where two different alleles are functional and regulated by the same background factor.** Here, the hollow circle means the background level that satisfy the full potential of the target. The filled red circle means the background level that homozygous parent actually supplied. The orange line indicates the background allocation. When the background is insufficient and the affinity of the two genes is different, the two allele of A and a will be in a competitive use of limited background, the more competitive gene is likely to get relative more backgrounds in hybrid than that in the original homozygous parents. The parameter of μ, K and n are described in the **Additional file 3**.

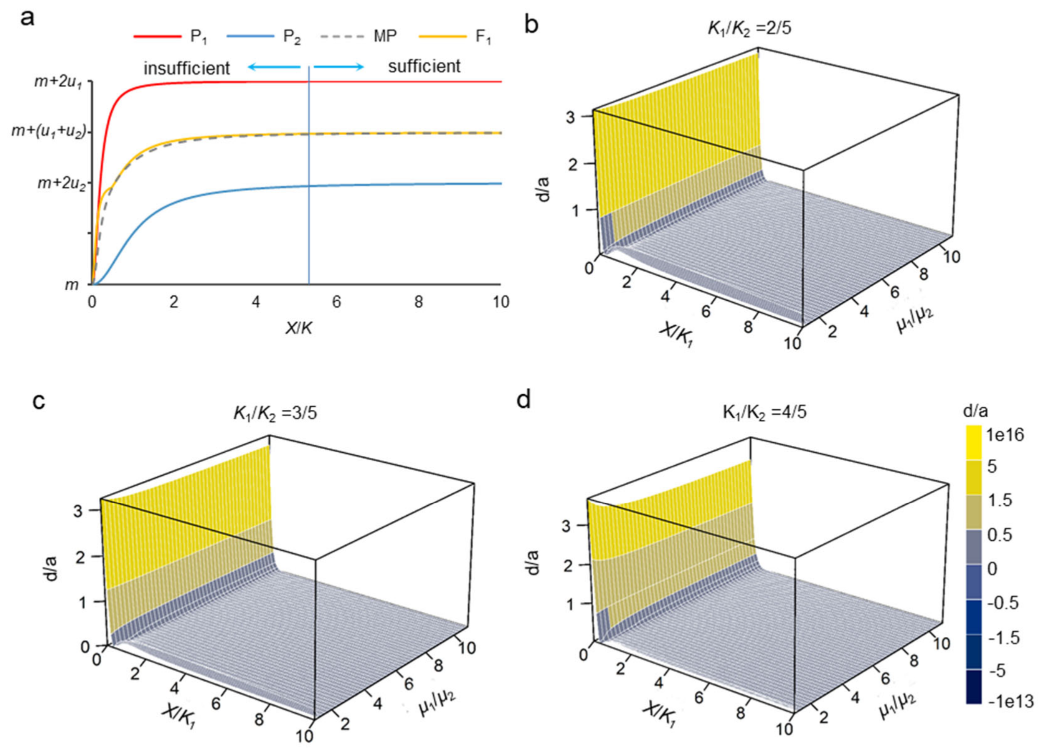


**Figure S32. The simulated diagram of regulation model for molecular mechanism of additive, dominant and overdominant effect produced by the cumulated functions of two alleles of one polymorphic site under the same positive regulators or responsors as the background when allele 1 showing larger maximum function and higher affinity ($\mu_1 > \mu_2$ and $K_1 < K_2$).** (a) The performance of the target site in two parents, $F_1$ and the middle parent (MP) under the activator background with different sufficiencies (*X/K*). It was simulated according to Hill function with $\mu_1 = 3$, $\mu_2 = 1$, $K_1 = 1$, $K_2 = 5$ and $n = 2$. $\mu_1$ and $\mu_2$ means the maximum function at steady state for allele with higher function and lower function, respectively. $n$ is the Hill coefficient. Left arrow represents a relatively insufficient activator background, and the right arrow represents the relatively sufficient activator background. (b) The dominant degree of the target site under the activator background with different sufficiencies for allele with higher function ($X/K_1$) of the target site and with different $\mu_1/\mu_2$ when $K_1/K_2 = 2/5$. (c) The dominant degree of the target site under the activator background with different sufficiencies for allele with higher function ($X/K_1$) of the target site and with different $\mu_1/\mu_2$ when $K_1/K_2 = 3/5$. (d) The dominant degree of the target site under the activator background with different sufficiencies for allele with higher function ($X/K_1$) of the target site and with different $\mu_1/\mu_2$ when $K_1/K_2 = 4/5$.

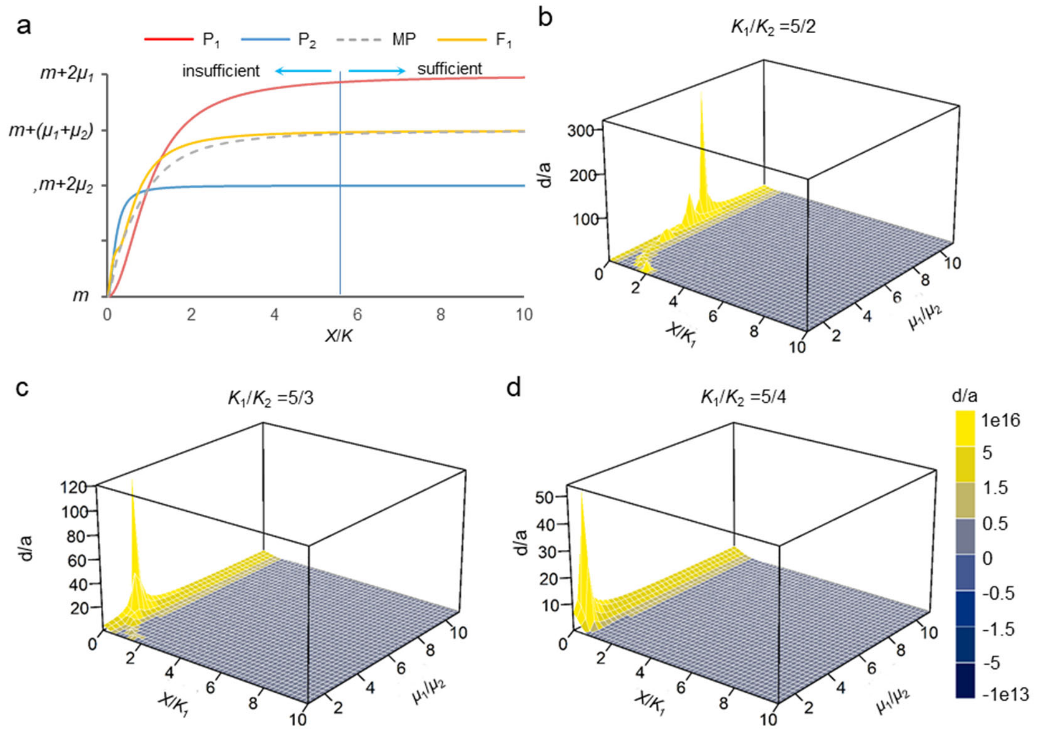


**Figure S33. The simulated diagram of regulation model for molecular mechanism of additive, dominant and overdominant effect produced by the cumulated functions of two alleles of one polymorphic site under the same positive regulators or responsors as the background when allele 1 showing larger maximum function but lower affinity ($\mu_1 > \mu_2$ and $K_1 > K_2$).** (a) The performance of the target site in two parents, $F_1$ and the middle parent (MP) under the activator background with different sufficiencies ($X/K$). It was simulated according to Hill function with $\mu_1 = 3$, $\mu_2 = 1$, $K_1 = 5$, $K_2 = 1$ and $n = 2$. $\mu_1$ and $\mu_2$ means the maximum function at steady state for allele with higher function and lower function, respectively. $n$ is the Hill coefficient. Left arrow represents a relatively insufficient activator background, and the right arrow represents the relatively sufficient activator background. (b) The dominant degree of the target site under the activator background with different sufficiencies for allele with higher function ($X/K_1$) of the target site and with different $\mu_1/\mu_2$ when $K_1/K_2 = 5/2$. (c) The dominant degree of the target site under the activator background with different sufficiencies for allele with higher function ($X/K_1$) of the target site and with different $\mu_1/\mu_2$ when $K_1/K_2 = 5/3$. (d) The dominant degree of the target site under the activator background with different sufficiencies for allele with higher function ($X/K_1$) of the target site and with different $\mu_1/\mu_2$ when $K_1/K_2 = 5/4$.

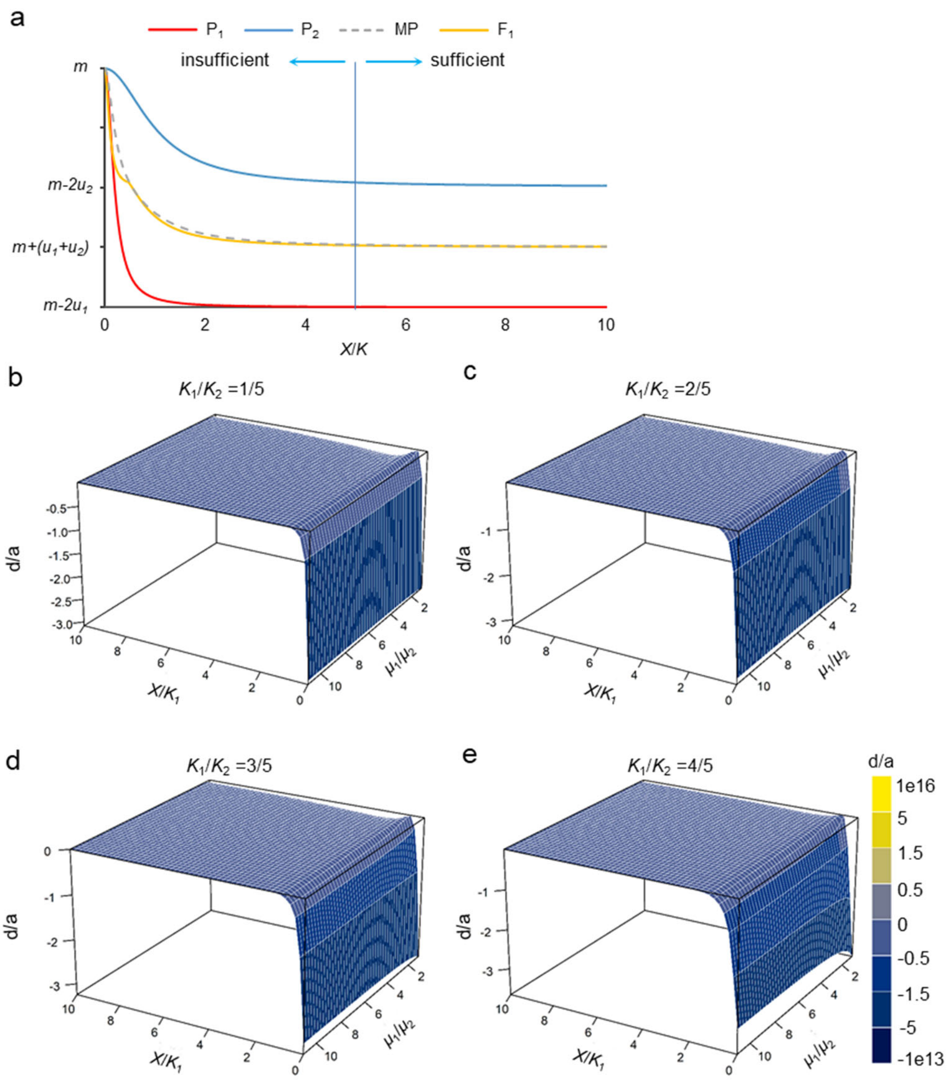


**Figure S34. The simulated diagram of regulation model for molecular mechanism of additive, dominant and overdominant effect produced by the cumulated functions of two alleles of one polymorphic site under the same negative regulators or responsors as the background when allele 1 showing larger maximum function and higher affinity ($\mu_1 > \mu_2$ and $K_1 < K_2$).** (a) The performance of the target site in two parents, $F_1$ and the middle parent (MP) under the repressor background with different sufficiencies ($X/K$). It was simulated according to Hill function with $\mu_1 = 2$, $\mu_2 = 1$, $K_1 = 1$, $K_2 = 5$ and $n = 2$. $\mu_1$ and $\mu_2$ means the maximum function at steady state for allele with higher function and lower function, respectively. $n$ is the Hill coefficient. Left arrow represents a relatively insufficient repressor background, and the right arrow represents the relatively sufficient repressor background. (b) The dominant degree of the target site under the repressor background with different sufficiencies for allele with higher function ($X/K_1$) of the target site and with different $\mu_1/\mu_2$ when $K_1/K_2 = 1/5$. (c) The dominant degree of the target site under the repressor background with different sufficiencies for allele with higher function ($X/K_1$) of the target site

and with different $\mu_1/\mu_2$ when $K_1/K_2$ = 2/5. (d) The dominant degree of the target site under the repressor background with different sufficiencies for allele with higher function ($X/K_1$) of the target site and with different $\mu_1/\mu_2$ when $K_1/K_2$ = 3/5. (e) The dominant degree of the target site under the repressor background with different sufficiencies for allele with higher function ($X/K_1$) of the target site and with different $\mu_1/\mu_2$ when $K_1/K_2$ = 4/5.

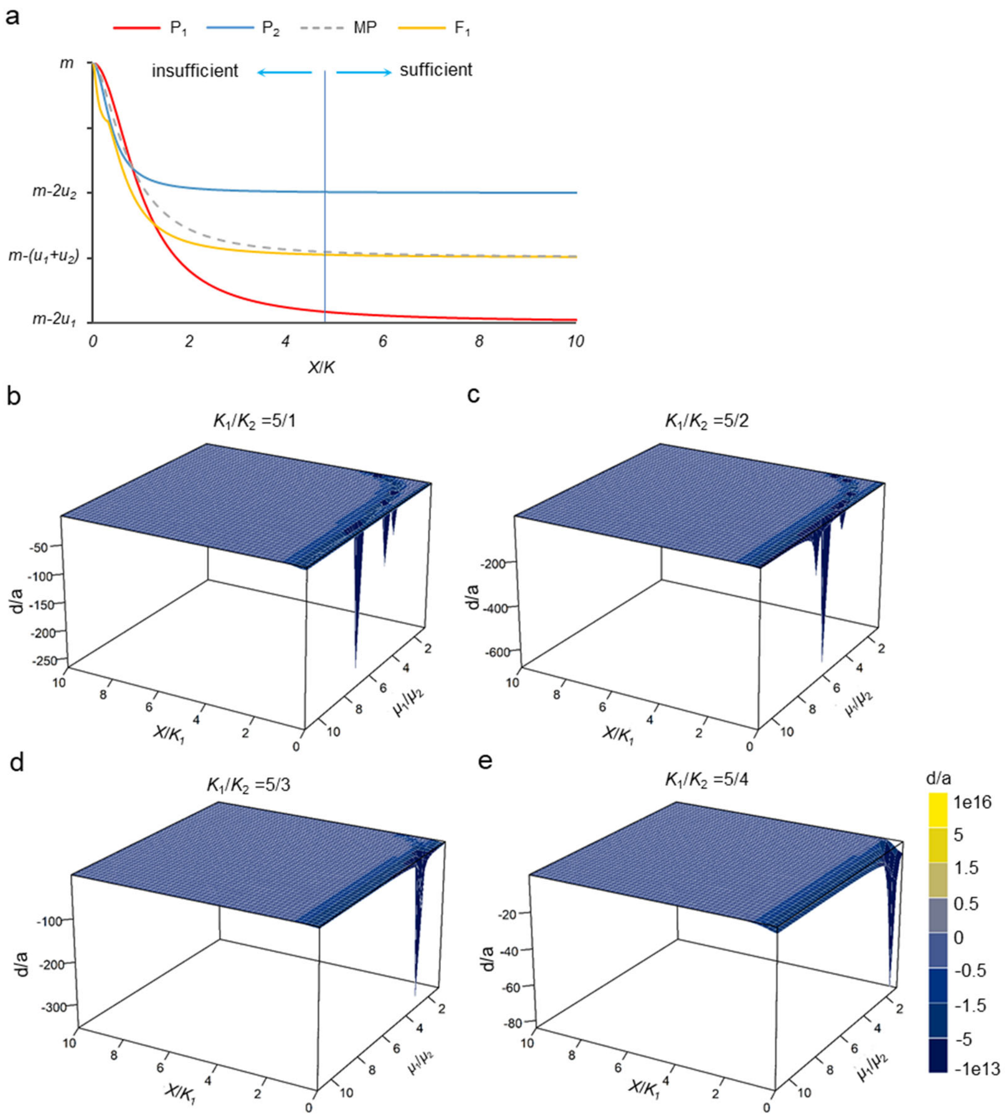


**Figure S35. The simulated diagram of regulation model for molecular mechanism of additive, dominant and overdominant effect produced by the cumulated functions of two alleles of one polymorphic site under the same negative regulators or responsors as the background when allele 1 showing larger maximum function but lower affinity ($\mu_1 > \mu_2$ and $K_1 > K_2$).** (a) The performance of the target site in two parents, $F_1$ and the middle parent (MP) under the repressor background with different

sufficiencies ($X/K$). It was simulated according to Hill function with $\mu_1 = 2, \mu_2 = 1, K_1 = 5, K_2 = 1$ and $n = 2$. $\mu_1$ and $\mu_2$ means the maximum function at steady state for allele with higher function and lower function, respectively. $n$ is the Hill coefficient. Left arrow represents a relatively insufficient repressor background, and the right arrow represents the relatively sufficient repressor background. (b) The dominant degree of the target site under the repressor background with different sufficiencies for allele with higher function ($X/K_1$) of the target site and with different $\mu_1/\mu_2$ when $K_1/K_2 = 5/1$. (c) The dominant degree of the target site under the repressor background with different sufficiencies for allele with higher function ($X/K_1$) of the target site and with different $\mu_1/\mu_2$ when $K_1/K_2 = 5/2$. (d) The dominant degree of the target site under the repressor background with different sufficiencies for allele with higher function ($X/K_1$) of the target site and with different $\mu_1/\mu_2$ when $K_1/K_2 = 5/3$. (e) The dominant degree of the target site under the repressor background with different sufficiencies for allele with higher function ($X/K_1$) of the target site and with different $\mu_1/\mu_2$ when $K_1/K_2 = 5/4$.

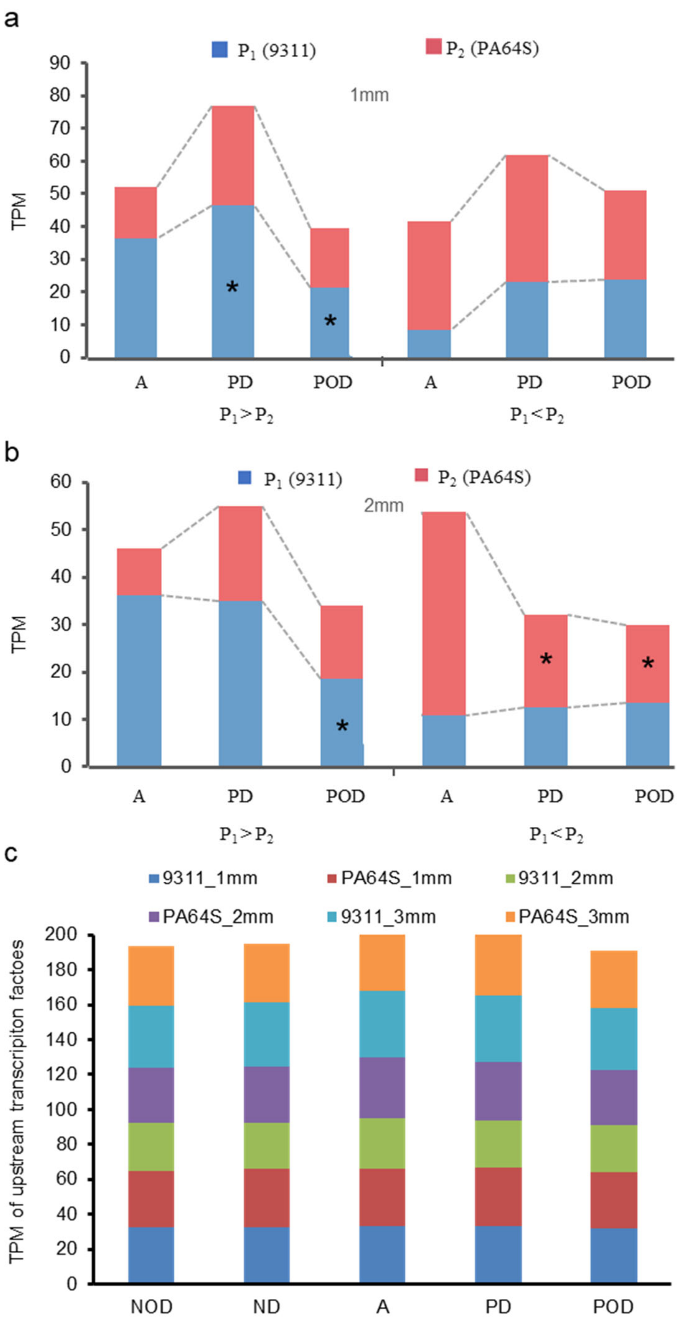


**Figure S36. The expression level of genes with different expression patterns in 1 mm and 2 mm young panicles of two parents.** (a-b) The expression level of genes with different expression patterns in 1mm and 2mm young panicle of two parents. Here, A, PD and POD mean the expression patterns appearing additive, positive dominant and overdominant, respectively; the asterisk means significant difference from the genes with additive expression pattern. (c) The cumulative expression of upstream transcription factor of genes of different expression patterns in 1mm, 2mm and 3mm young panicle of two parent.

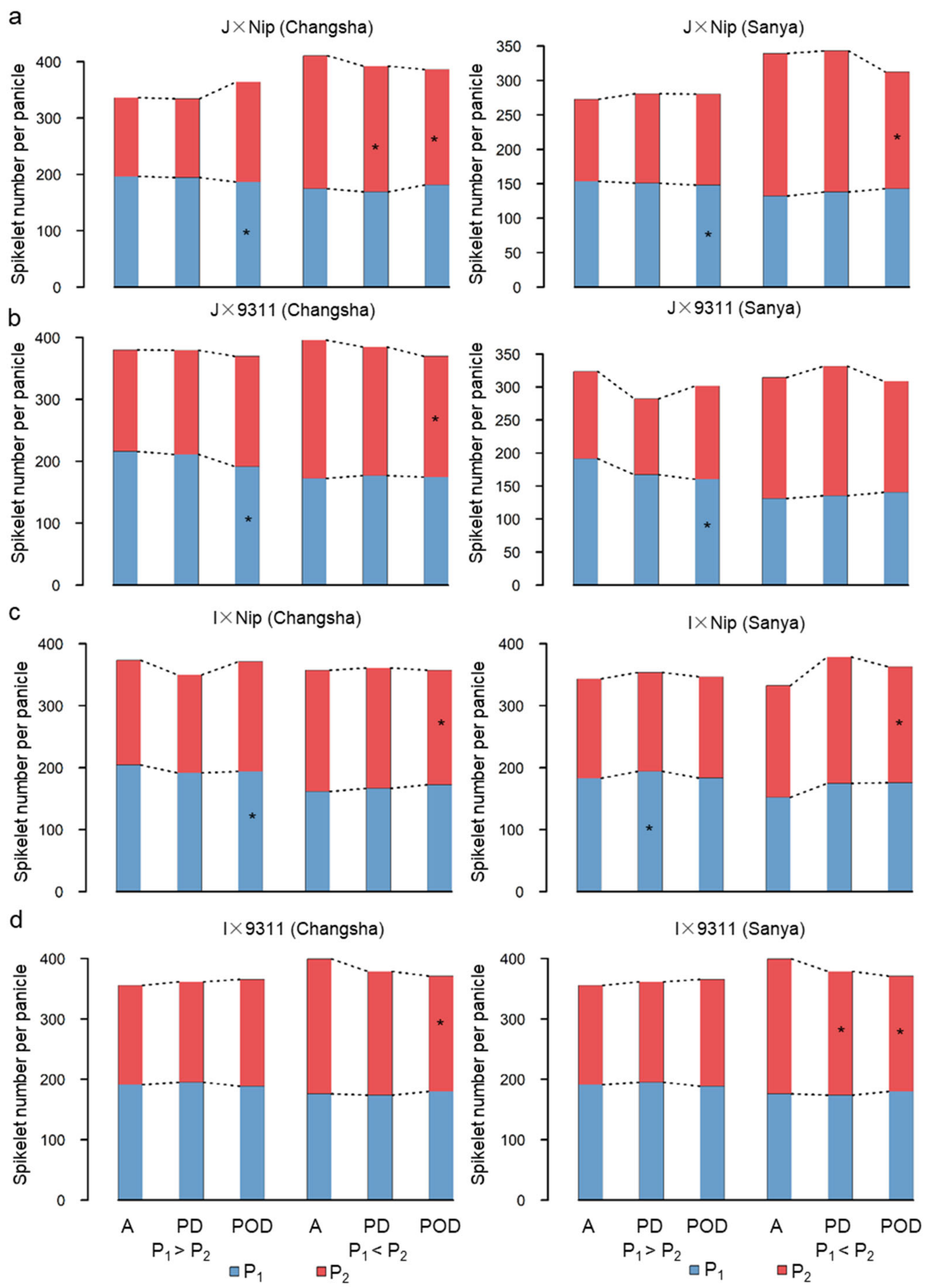


**Figure S37. The spikelet number per plant (SPP) of parents with non-tester genotype ($P_1$) and parents with tester genotype ($P_2$) of the SPP QTLs showing different genetic effect types**. Here, A, PD and POD mean the additive, positive dominant and positive overdominant QTLs, respectively. $P_1 > P_2$ means that $P_1$ contains the genotype with higher effect of the QTL, and on the contrary $P_1 < P_2$ means that $P_2$ contains the genotype with higher effect of the QTL. The asterisk means significant difference from the addtive QTLs.

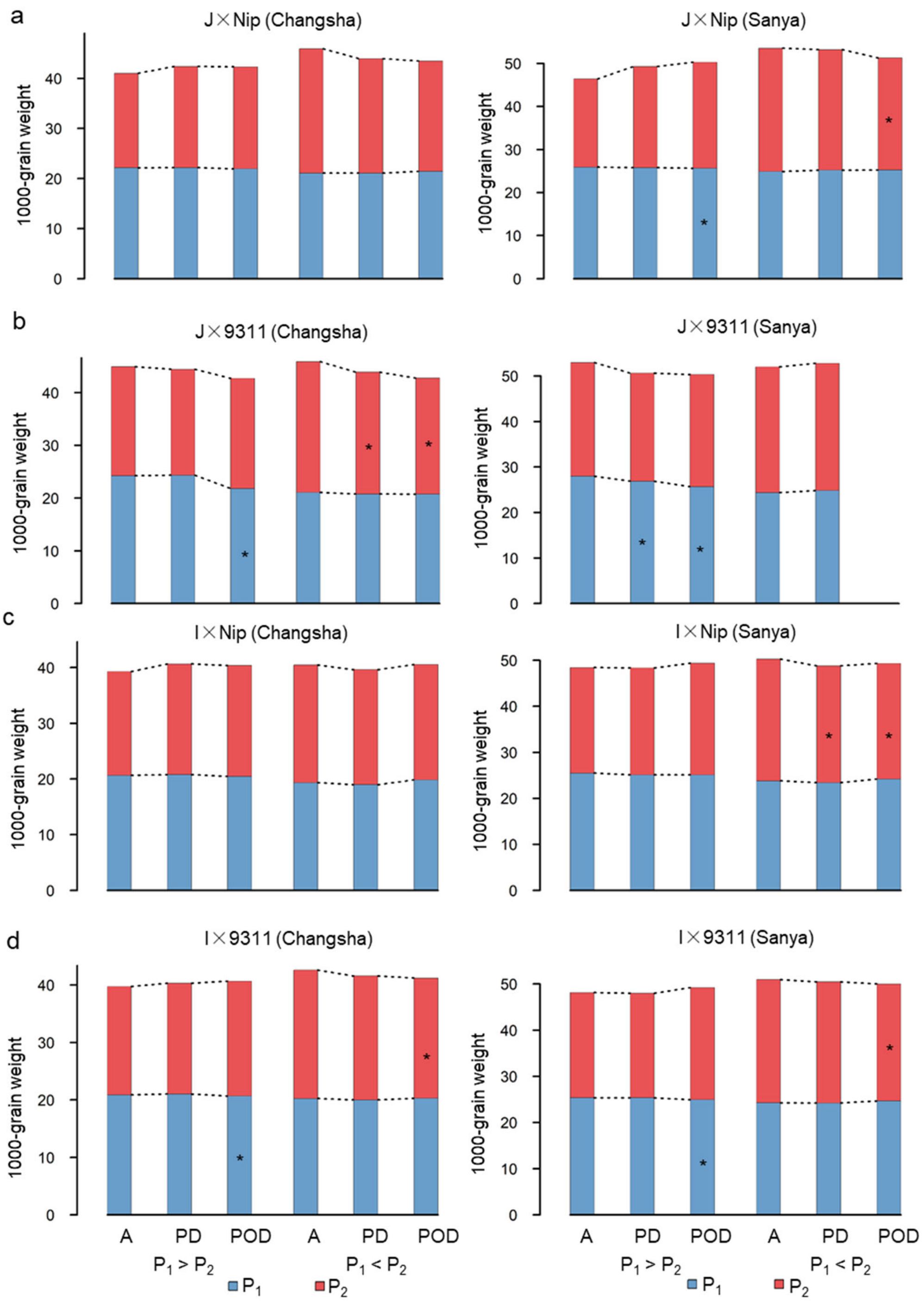


**Figure S38. The 1000-grain weight (KGW) of parents with non-tester genotype ($P_1$) and parents with tester genotype ($P_2$) of the KGW QTLs showing different genetic effect types**. Here, A, PD and POD mean the additive, positive dominant and positive overdominant QTLs, respectively. $P_1 > P_2$ means that $P_1$ contains the genotype with higher effect of the QTL, and on the contrary $P_1 < P_2$ means that $P_2$ contains the genotype with higher effect of the QTL. The asterisk means significant difference from the addtive QTLs.

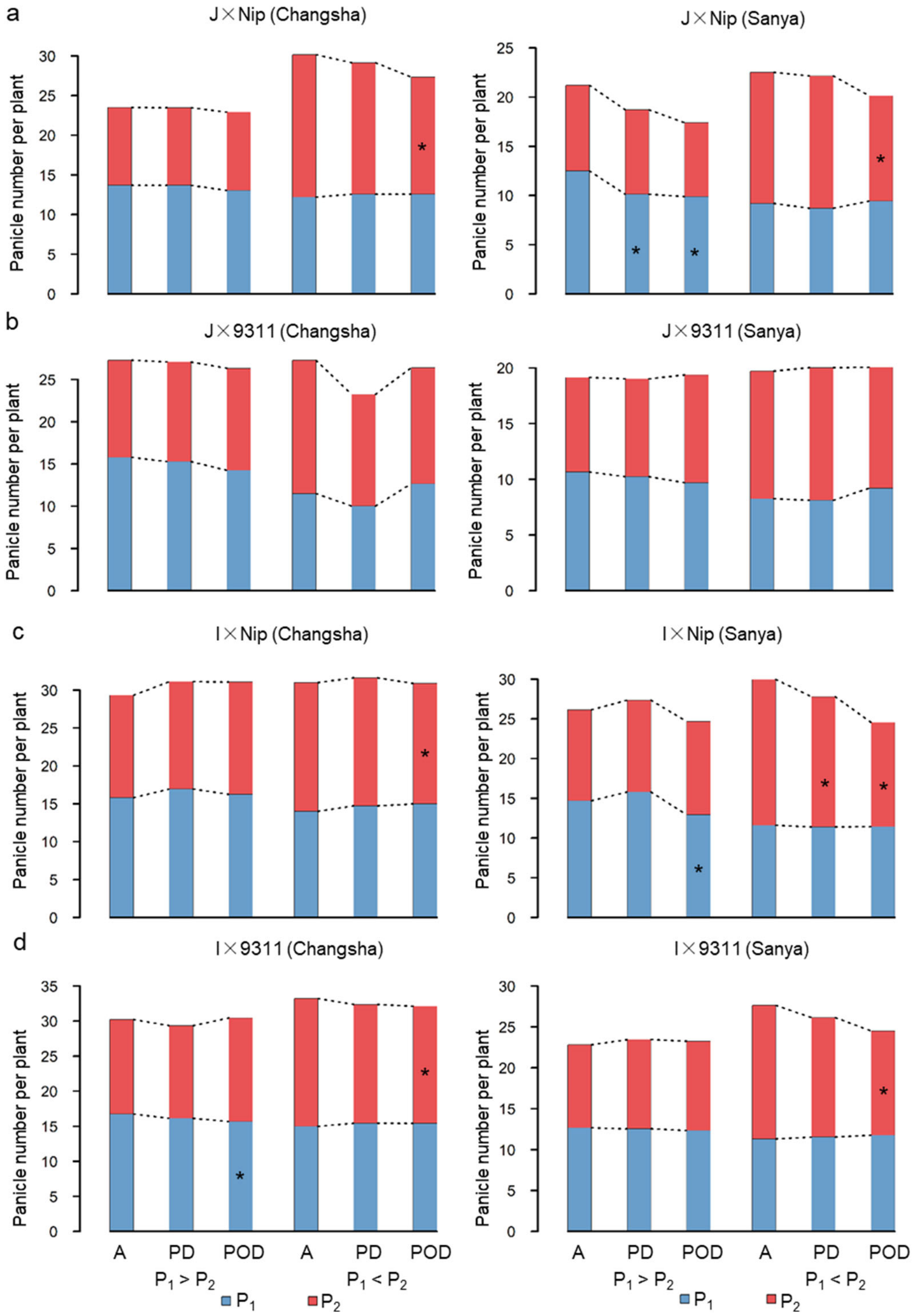


**Figure S39. The panicle number per plant (PNP) of parents with non-tester genotype ($P_1$) and parents with tester genotype ($P_2$) of the PNP QTLs showing different genetic effect types**. Here, A, PD and POD mean the additive, positive dominant and positive overdominant QTLs, respectively. $P_1 > P_2$ means that $P_1$ contains the genotype with higher effect of the QTL, and on the contrary $P_1 < P_2$ means that $P_2$ contains the genotype with higher effect of the QTL. The asterisk means significant difference from the addtive QTLs.

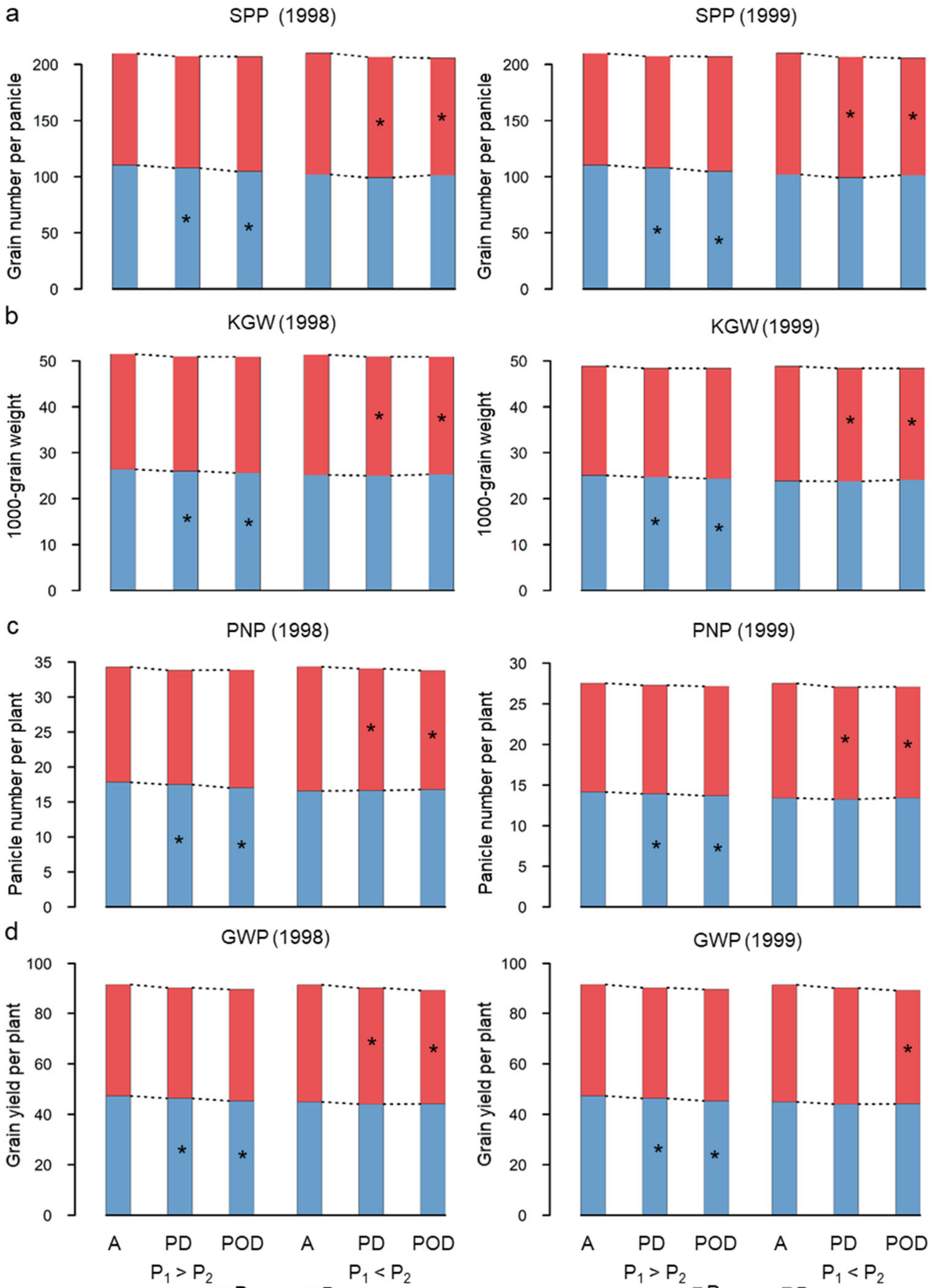


**Figure S40. The yield traits of lines with Zhenshan97 ($P_1$) genotype and Minghui63 ($P_2$) genotype of those QTLs with different genetic effect types in IMF$_2$ population in 1998 and 1999.** Here, A, PD and POD mean the additive, positive dominant and positive overdominant QTLs, respectively. $P_1 > P_2$ means that $P_1$ contains the genotype with higher effect of the QTL, and on the contrary $P_1 < P_2$ means that $P_2$ contains the genotype with higher effect of the QTL. The asterisk means significant difference from the addtive QTLs. The QTLs were identified according to the published data (Zhou et al. Genetic composition of yield heterosis in an elite rice hybrid. Proc Natl Acad Sci U S A 2012, 109:15847-15852).

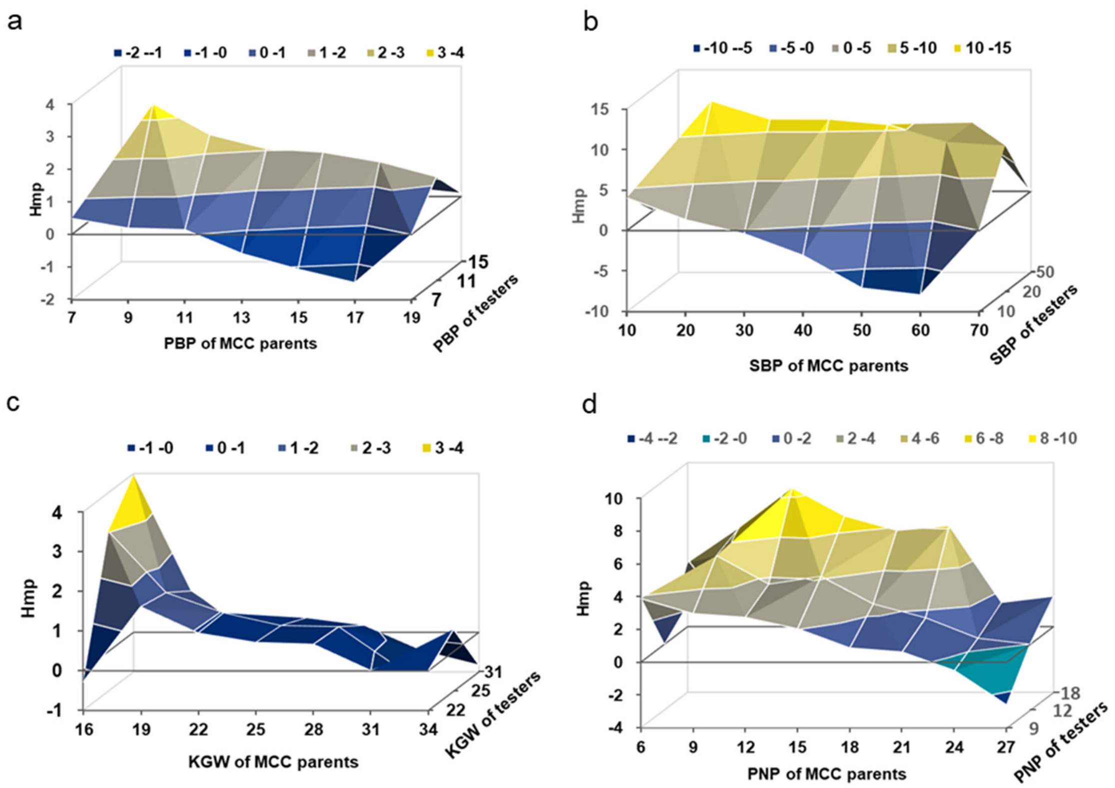


**Figure S41. The relationship between the middle-parent heterosis (Hmp) and the phenotypes of their parents for primary branch number per panicle (a), secondary branch number per panicle (b), 1000-grain weight (c) and panicle number per plant (d).** The results were calculated according to the phenotype of 418 combinations of MCC in Changsha and Sanya.

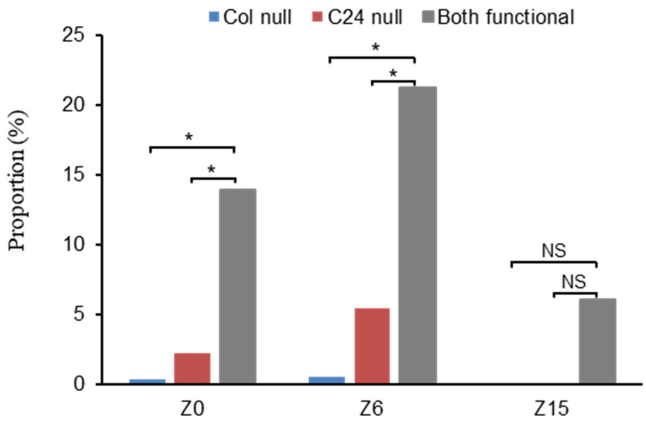


**Figure S42. The proportion of overdominant expressed genes under the condition of one parental allele is functional and both parental alleles are functional.** The proportion of overdominant expressed genes in arabidopsis hybrid Col×C24 under the condition of one parental allele is functional and both parental alleles are functional. Genes completely not expressed in the ZT0 , ZT6 and ZT15 young leaves of Col, but expressed in all of ZT0 , ZT6 and ZT15 young leaves of C24 were defined as Col null; Conversely, genes completely not expressed in the ZT0 , ZT6 and ZT15 young leaves of C24, but expressed in all of ZT0 , ZT6 and ZT15 young leaves of Col were defined as C24 null; The genes expressed in all of the ZT0 , ZT6 and ZT15 young leaves of both Col and C24 were defined as two parental alleles are functional (Both functional). Z0, Z6 and Z15 means zeitgeber time ZT0 (dawn), ZT6 (midday) and ZT15 (dusk). The level of statistical significance was derived from the Chi-square test.

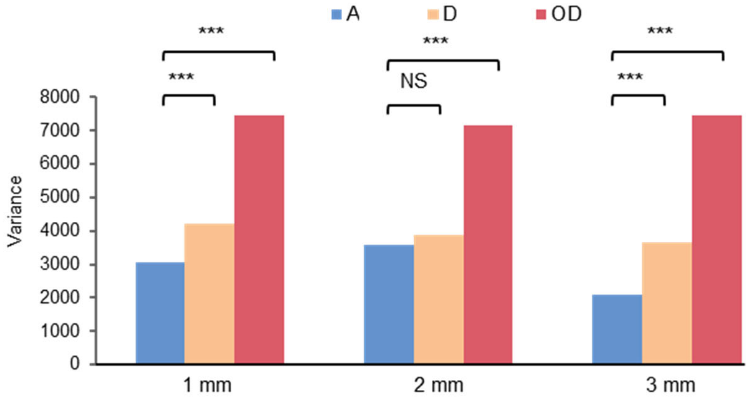


**Figure S43. The variance of expression levels among different tissues for genes with additive, dominant and overdominant expression patterns in 1mm, 2mm or 3mm young panicles of hybrids**. Triple-asterisk means significant difference with $p < 0.001$; NS means no significance. The variance was estimated from 48 dataset collected from ricexpro (http://ricexpro.dna.affrc.go.jp/).

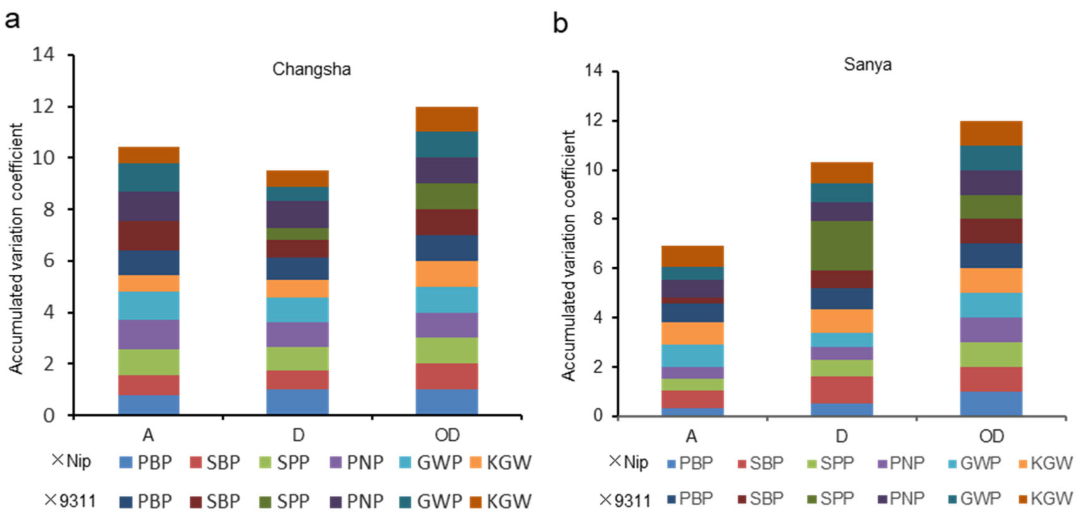


**Figure S44. The variance of different traits among environments for different degrees of dominant effects.** (a) The accumulated variation coefficient of 6 yield related trait in Changsha. (b) The accumulated variation coefficient of 6 yield related trait in Sanya.

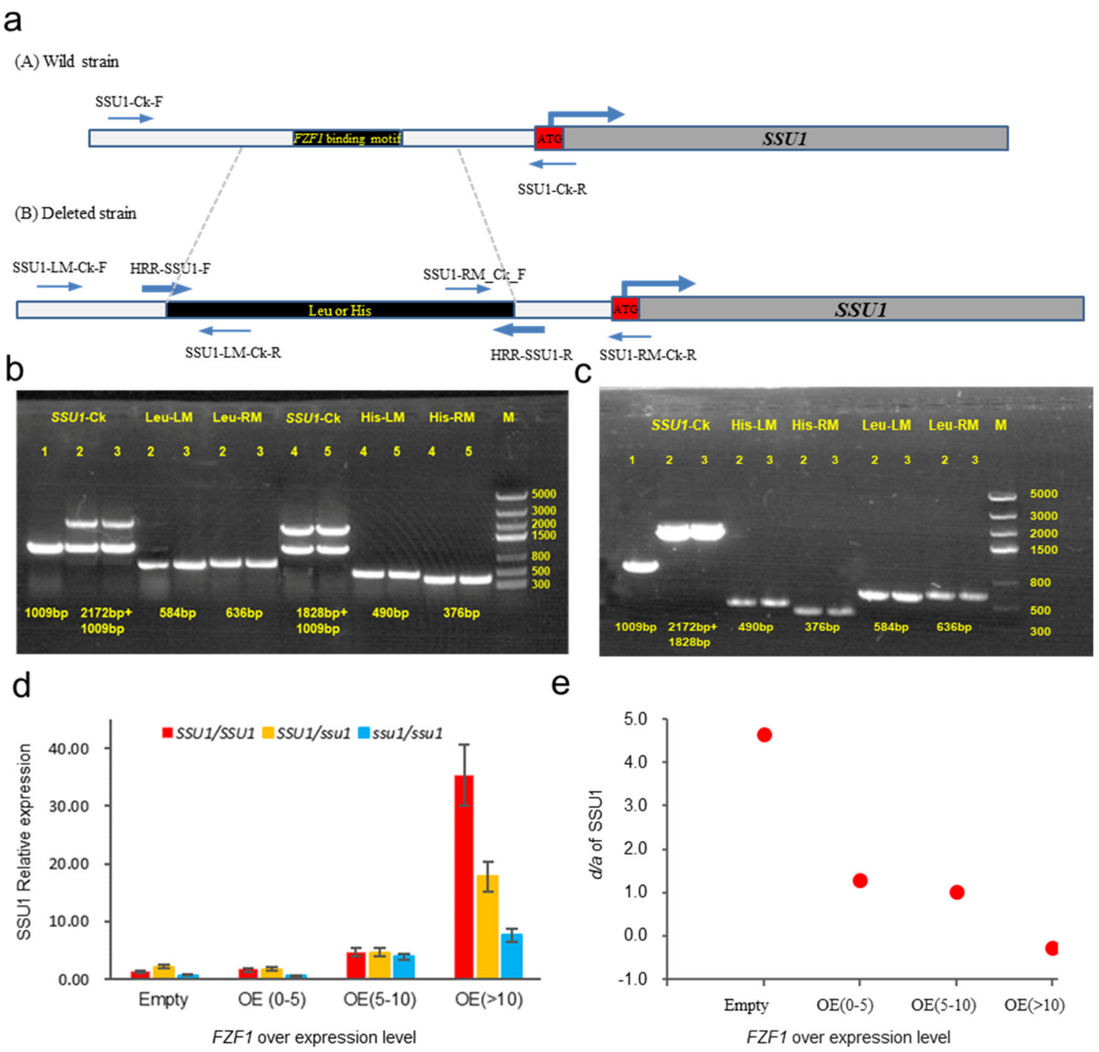


**Figure S45. Experimental validation of HoIIB model in diploid *Saccharomyces cerevisiae.*** (a) Primer design principles and requirements. SSU1_Ck-F and SSU1_Ck-R are located on both side of the transcription factor *FZF1* recognition motif in the *SSU1* gene promoter. SSU1-LM-Ck-R and SSU1-RM-Ck-F are located in inserted marker (marker can be Leu or His); HRR-SSU1-F and HRR-SSU1-R had 38bp homologous sequences on both sides of the *FZF1* recognition motif in *SSU1* promoter, and the outer 21bp sequence was the upstream and downstream primers for screening markers genes on the amplified plasmid (pfa6a-leu1mx or pFA6a-His3MX6). (b) 1 was genomic fragment containing the *FZF1* recognition motif in the wild type (BY4743), 2 and 3 were the genomic fragment (2.1kb and 1kb respectively) of heterozygous mutant that one copy of *FZF1* recognition motif was substituted by Leu and the other was remain unchanged; 4 and 5 were the genomic fragment (1.8kb and 1kb respectively) of heterozygous mutant that one copy of *FZF*1 binding motif was substituted by His and the other was remain unchanged; Leu-LM was the primer used to amplify the left DNA fragment of Leu substitution genotype, Leu-RM was the primer used to amplify the right DNA fragment of Leu substitution genotype, His-LM was the primer used to amplify the left DNA fragment of His

substitution genotype, Leu-RM was the primer used to amplify the right DNA fragment of Leu substitution genotype. (c) 1 was genomic fragment containing the *FZF1* recognition motif in the wild type (BY4743), 2 and 3 were the genomic fragment (2.1kb and 1kb respectively) of diploid mutant that one copy of *FZF1* recognition motif was substituted by Leu and the other was substituted by His; (d) The relative expression of gene *SSU1* in different *SSU1* genotypes under different expression levels of its transcription factor (*FZF1*) in *Saccharomyces cerevisiae* BY4743; here, *SSU1*/*SSU1*, *ssu1*/*ssu1* and *SSU1*/*ssu1* represent the homologous genotype of wild type, the homologous genotype of mutant, and their heterozygous genotype, respectively; OE(0-5) means the strain with upregulated *FZF1* by 0-5 folds, and similar for OE(5-10) and OE(>10), and Empty means the strain with empty vector free of *FZF1*. (e) The dramatically decreased dominance degree of *SSU1* along with the increase of upregulation levels of its upstream transcription factor *FZF1* in *Saccharomyces cerevisiae* BY4743.

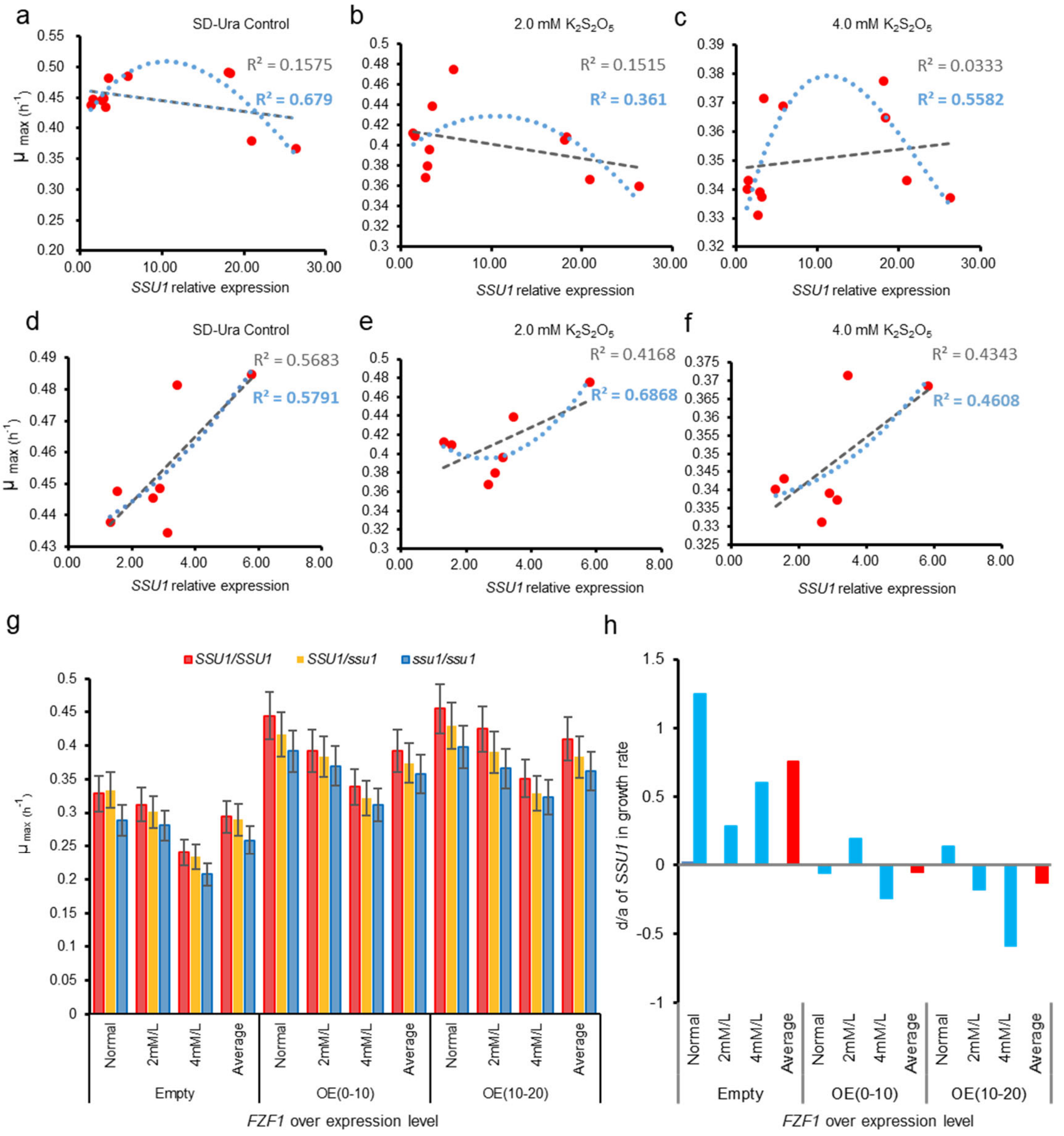


**Figure S46. The results of preliminary validation of HoIIB model in diploid Saccharomyces cerevisiae.** (a-c) The relationship between the expression level of *SSU1* and the maximum growth rate of wild-type BY4743 in normal SD-Ura medium, SD-Ura with 2mM and SD-Ura with 4mM $K_2S_2O_5$ under different *FZF1* overexpression levels (1-85 times). **(d-f)** The relationship between the expression level of *SSU1* and the maximum growth rate of wild-type BY4743 in normal SD-Ura, SD-Ura with 2mM and SD-Ura with 4mM $K_2S_2O_5$ under *FZF1* overexpression level less than 20 times. The upper, lower of R-squares are the determination coefficients of linear and second order polynomial curve models respectively. (g) The growth rate of gene *SSU1* in different *SSU1* genotypes under different expression levels of its upstream transcription factor (FZF1) in Saccharomyces cerevisiae BY4743; here, *SSU1*/*SSU1*, *ssu1*/*ssu1* and *SSU1*/*ssu1* represent the homologous genotype of wild type, the homologous genotype of mutant, and their heterozygous genotype, respectively; OE (0-10) means the strain with upregulated *FZF1* by 0-10 folds, and similar for OE (10-20) ,

Empty means the strain with empty vector free of *FZF1*. (h) The obviously decreased dominance degree of *SSU1* along with the increase of upregulation levels of its transcription factor *FZF1* in Saccharomyces cerevisiae BY4743.

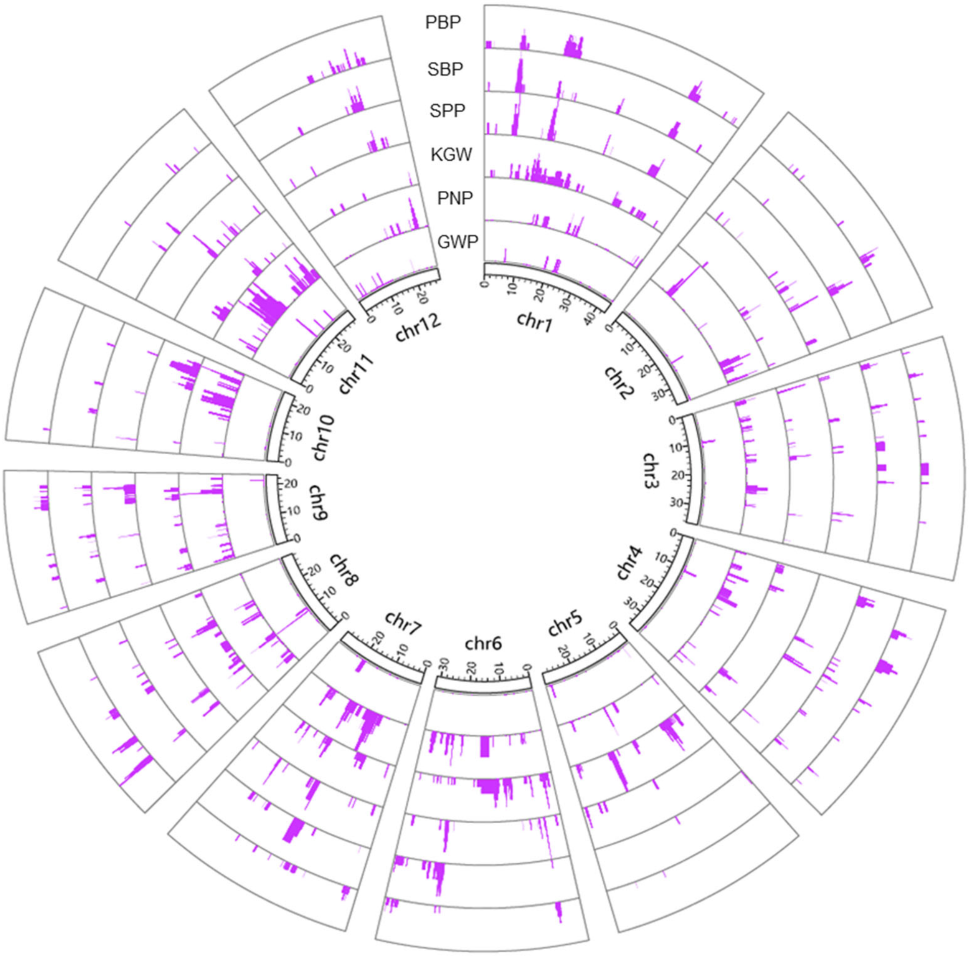


**Figure S47. The times of associated genes that can be repeatedly identified in the dominance and overdominance QTLs across four kinds of combinations and two environments**. Each line represents one associated gene. From the inner to outer layer, the height of lines represents 2-4 time for GWP, 3-6 times for PNP, 3-8 times for KGW, 3-6 times for SPP, 3-7 times for SBP and 3-8 times for PBP, respectively.

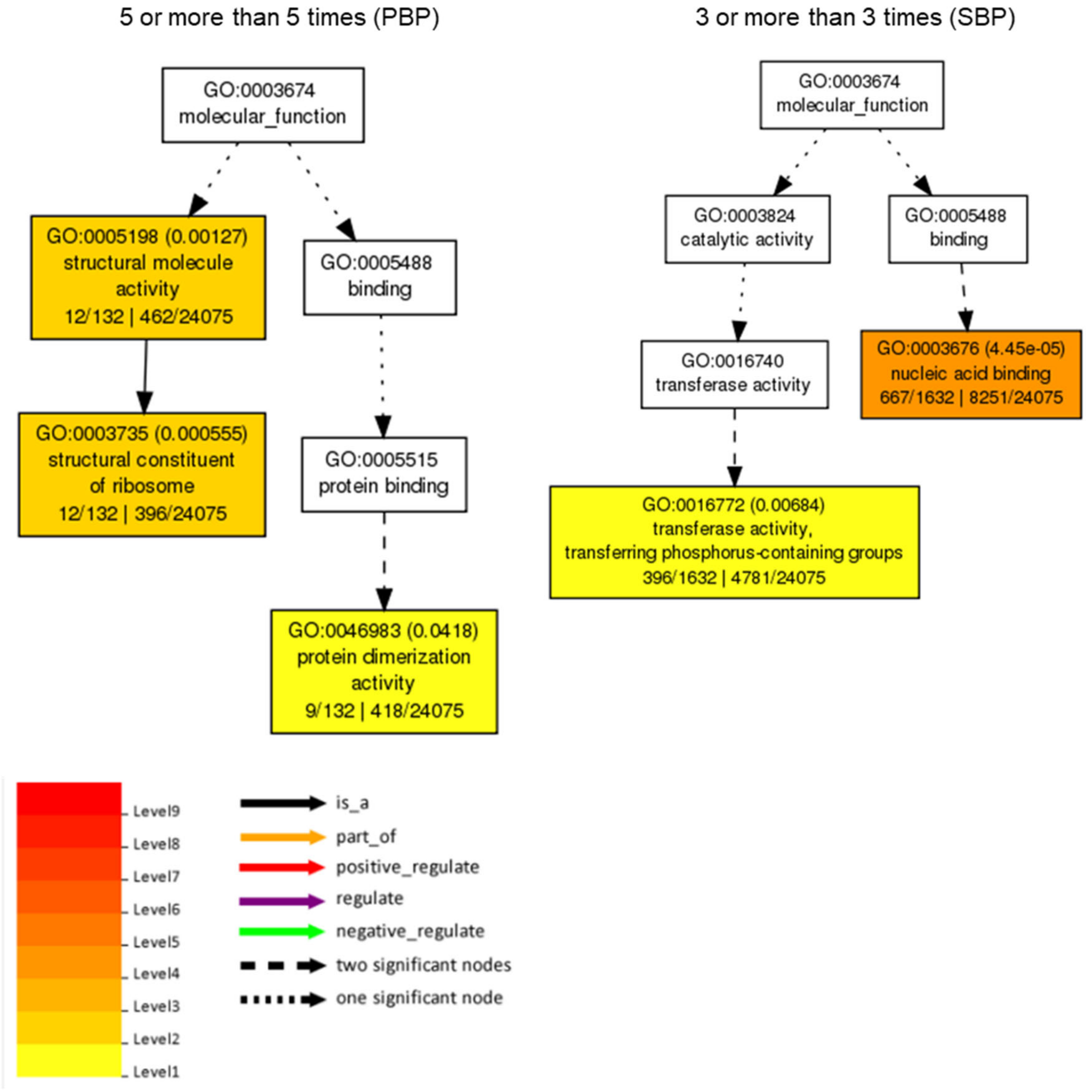


**Figure S48. The GO enrichment of repeated identified nonadditive genes in PBP, SBP, SPP, KGW, PNP and GWP across four kinds of combination and two environments for rice.** Only the term of molecular function was showed in figure, the other results were prepared in **Dataset S13**.

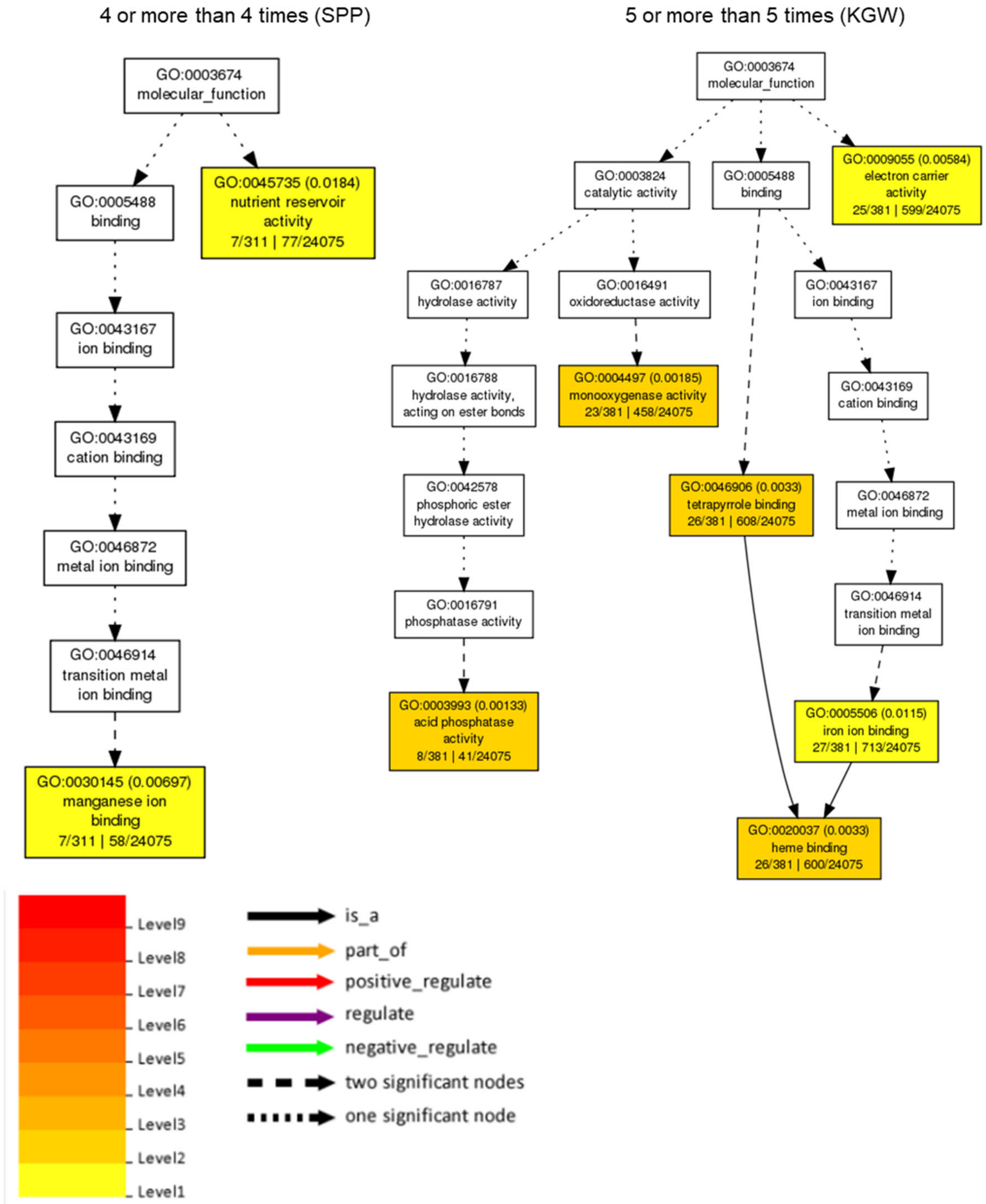


**Figure S48. continued**

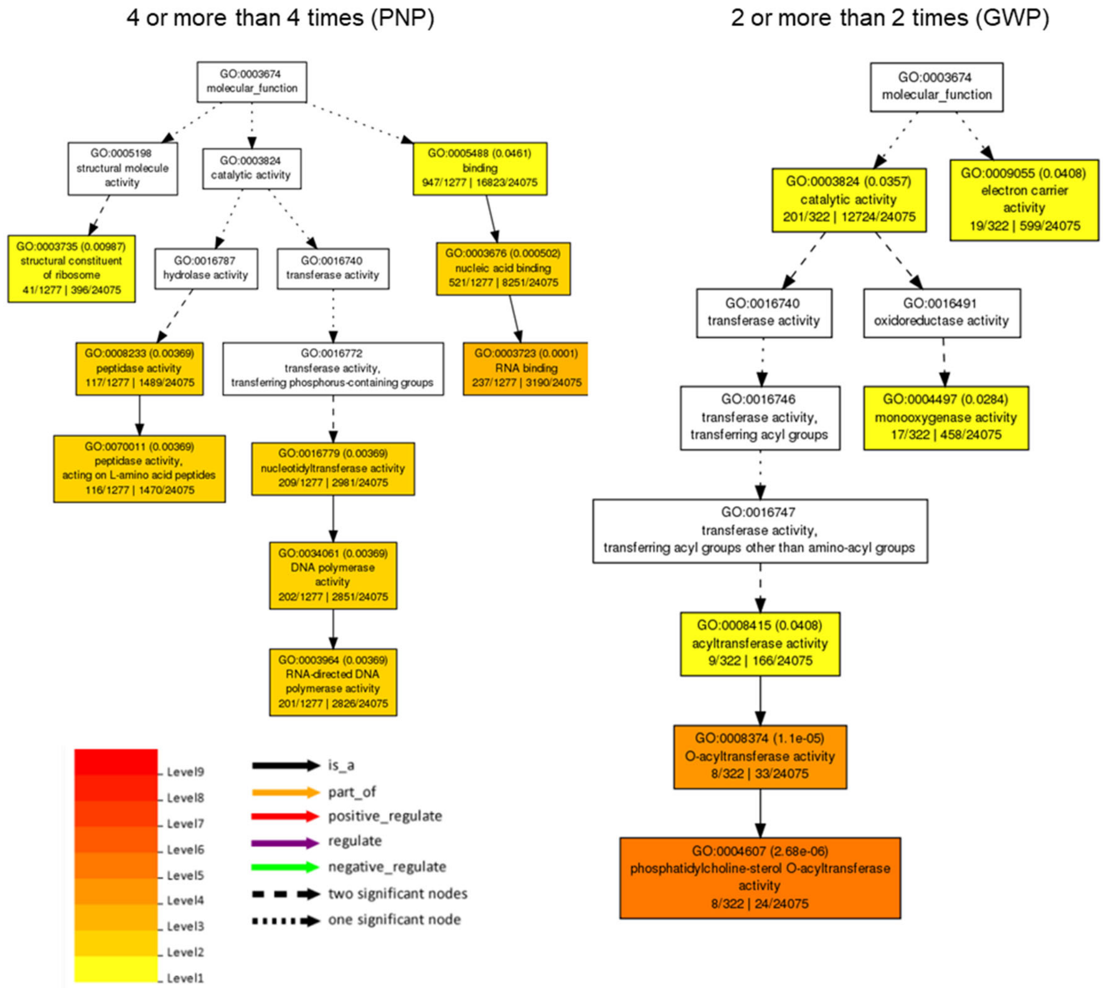


**Figure S48. continued**

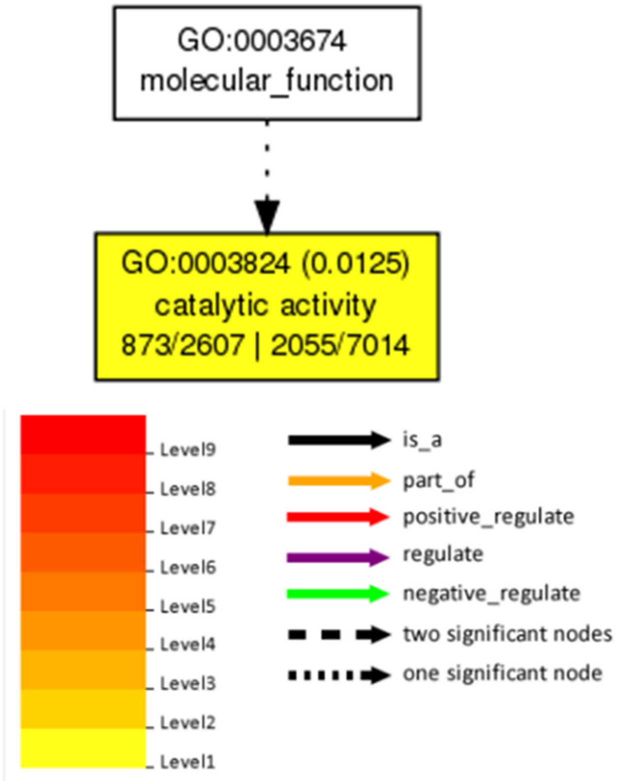


**Figure S49. The GO enrichment of repeated identified genes with nonadditive performance in non-lethal deletion yeast strains grown in five media.** Only the term of molecular function were showed in the figure.

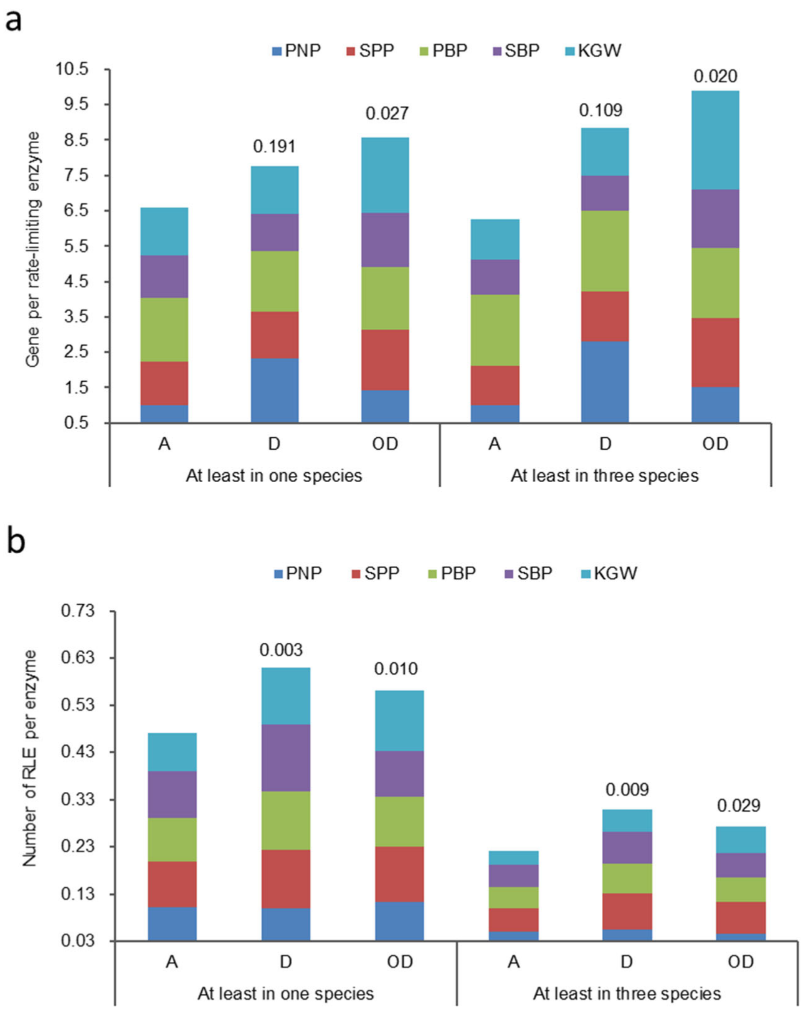


**Figure S50. The distribution of rate-limiting enzymes coded by candidate genes within additive, dominant and overdominant QTLs related to five yield component trait including PBP, SBP, SPP, KGW and PNP.** The rate-limiting enzymes were identified in species yeast, mouse, and Human. (a) the number of genes per rate limiting enzyme for additive, dominance and overdominance candidate genes. (b) the number rate-limiting enzymes per enzyme for additive, dominance and overdominance candidate genes. The P value at the top of bar means significant level by *T* test for comparison with additive ones.

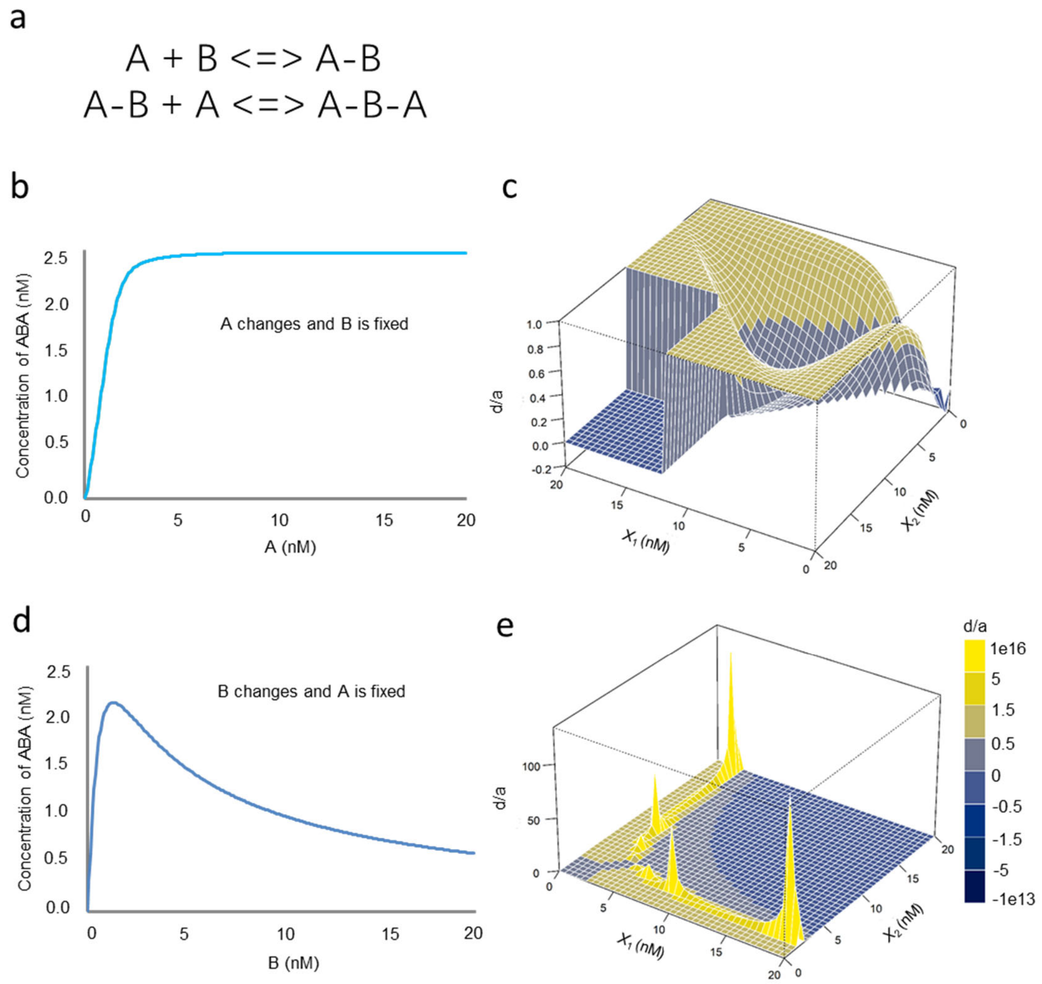


**Figure S51. The simulated diagram of regulation model for molecular mechanism of additive and dominant effect produced by single site in relation to the assembly of functional trimer ABA**. (a) The schematic diagram depicting the assembly of trimer ABA. In this model, the nonfunctional dimer AB can compete with the functional trimer ABA for the subcomponent B. (b) The simulated curve of ABA concentration in the equilibrium state with different concentrations of subcomponent A and with the subcomponent B constant. The sigmoidal curve indicates that the sensitivity of ABA concentration to the background of subcomponent A will decrease along with the increase of A concentration, and a nearly unchanged when the concentration of A reaches more than 2 times the concentration of B. (c) The dominant degree of the timer ABA produced by different concentrations of subcomponent A in two parents ($X_1$ and $X_2$). (d) The simulated curve of ABA concentration in the equilibrium state with different concentrations of subcomponent B and with the subcomponent A constant. The curve showed that, with the increase of B, ABA concentration initially increased with the increase of B concentration, but gradually decreased after reaching stoichiometric equilibrium. (e) The dominant degree of the timer ABA produced by different concentrations of subcomponent B in two parents ($X_1$ and $X_2$). All the simulated results indicated that the

nonadditive effect usually generates under insufficient background. Detail of the model and the values of parameters can be found in **Additional file 3.**

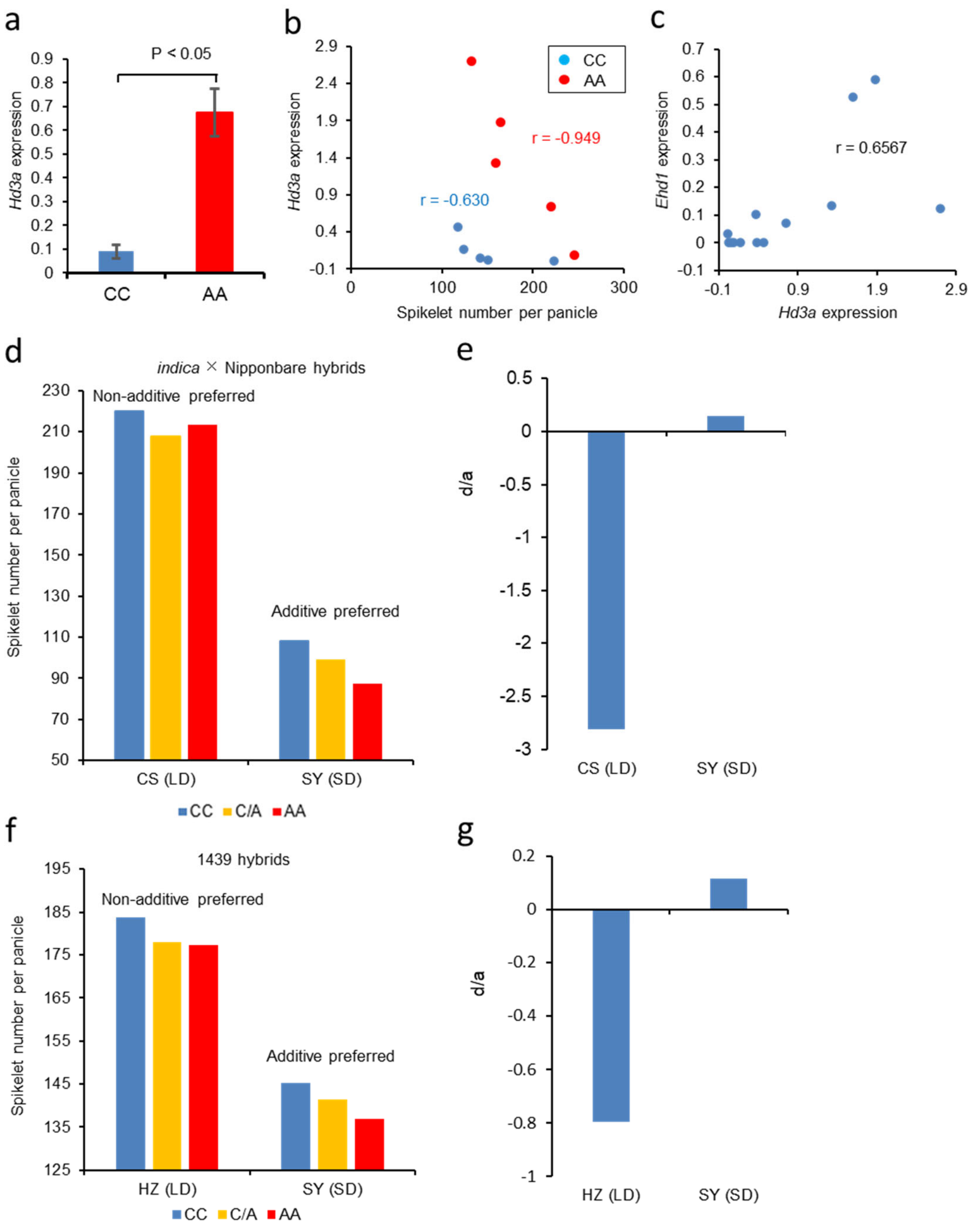


**Figure S52. The relationship between the expression of *Hd3a* and the spikelet number per panicles and heterotic performance of *Hd3a* under long day (LD) and short day (SD) environments**. (a) Expression levels of two *Hd3a* genotypes in Changsha environments. (b) The relationship between the expression level of *Hd3a* and spikelet number per panicle. (c) The relationship between the expression level of *Hd3a* and *Ehd1*. (d) The phenotypic performance of different *Hd3a* genotypes in *indica* × Nipponbare hybrids under

long day of Changsha (CS) and short day of Sanya (SY) environments. (e) The dominance degree of *Hd3a* in *indica* × Nipponbare hybrids under Changsha and Sanya environments. (f) The phenotypic performance of different *Hd3a* genotypes in 1439 hybrids under long day of Hangzhou (HZ) and short day of Sanya (SY) environments. (g) The dominance degree of *Hd3a* in 1439 hybrids under Hangzhou and Sanya environments.

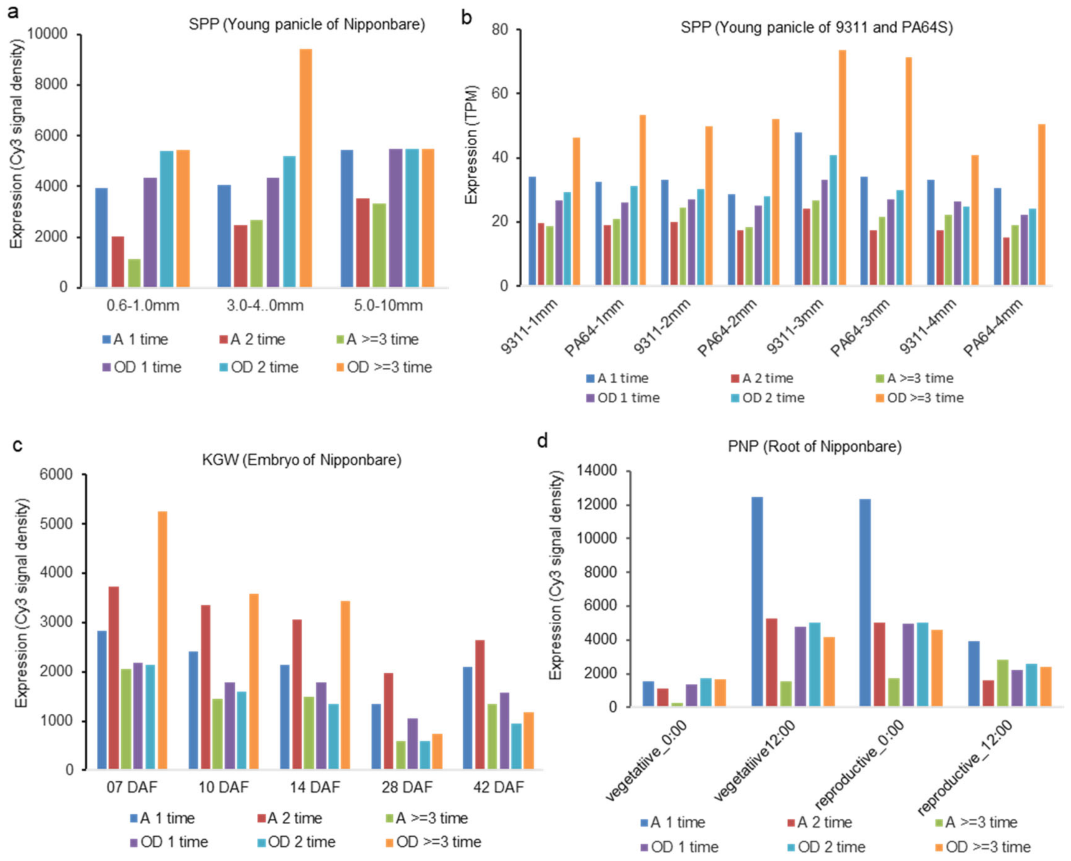


**Figure S53. The expression level of associated candidate genes within additive (A) and over dominant (OD) QTLs.** (a) The expression level of associated candidate genes of SPP QTLs in young panicles of Nipponbare. (b) The expression level of candidate genes of SPP QTLs in the young panicles of 9311 and PA64S. (c) The expression level of associated candidate genes of KWG QTLs in the embyro of Nipponbare. (d) The expression level of associated candidate genes of PNP QTLs in the root of Nipponbare. The raw data of gene expression in (a), (c) and (d) were obtained from the database of RiceXpro. 1 time, 2 time and >=3 times means those candidate genes within the QTLs that can be detected in 1, 2, and 3 or more than 3 situations among four combinations under two environments.

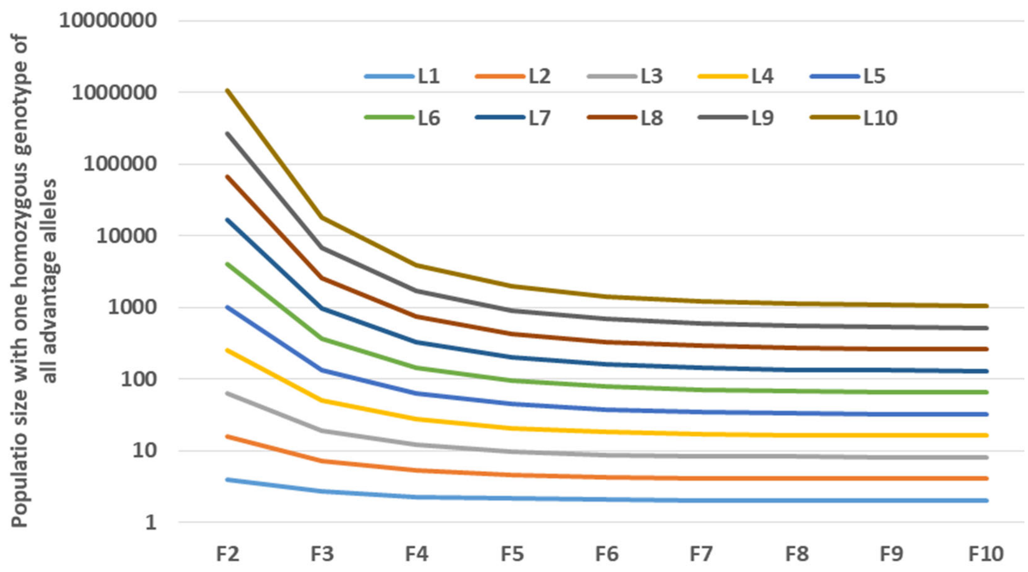


**Figure S54. The theoretical population size with at least one homozygous genotype of all advantage alleles in different generations of two parents with different numbers of polymorphic loci (from 1 to 10).** Here, we calculate the population under the hypothesis that there is no linkage between loci and all loci are randomly combined; L1 - L10 mean the locus number from 1 to 10; F2 - F10 mean the self-crossing generations from 2 to 10.
