# Supplementary material for: Large-scale genomic and transcriptomic profiles of rice hybrids reveal a core mechanism underlying heterosis
Source: Genome Biol. 2022 Dec 22;23:264. doi: 10.1186/s13059-022-02822-8 (PMC9773586; doi:10.1186/s13059-022-02822-8)
Supplement: Supplementary file 3 — Additional file 3. Supplementary methods [84–87]. [file 13059_2022_2822_MOESM3_ESM.pdf]

**Simulation1: the model reflecting the performance of homozygotes and heterozygote of one locus according to Hill equation**

The typical physiological theory had pointed that most of the mutants of genes encoding enzymes are dominant [50], which has been preliminarily verified in yeast and human [56]. Recent studies also noticed that enzyme unsaturation caused by insufficient substrate may be the direct cause of dominance [51]. However, it is still not clear whether the insufficient substrate background is the general mechanism of dominance or even overdominance, and the relationship between the sufficiency of the background and the occurrence of dominant or overdominance is still not clearly elucidated in literature. In this study, we systematically simulated occurrence of additive, dominant to overdominant inheritance of target receptor genes under different level of ligand background supply.

It is generally recognized that a ligand $X$ binds to a receptor $Y$ and reacts to produce a product is a common mechanism in biology [84]. Dynamically, the number of molecule product of $Y$ produced per unit time is a function of the concentration of ligand $X$ on its active form $X^*$:

$$\text{Production rate of } Y = f(X^*) \qquad (1)$$

Typically, the input function of $f(X)$ is a monotonic, S-shaped function. It is an increase function when $X$ is an activator and a decrease one when $X$ is a repressor [85]. The Hill input function for an activator is a curve that rises from zero and approaches a maximal saturated level:

$$f(X^*) = \frac{\beta X^{*n}}{K^n + X^{*n}} \qquad \text{Hill function for activator (2)}$$

The Hill function has three parameters, $K$, $\beta$ and $n$.

Parameter $K$ is termed as the activation coefficient, and has units of concentration. It defines the concentration of active $X$ needed to significantly activate production. From the equation, we can see that half-maximal production is reached when $X = K$. The value of $K$ related to the chemical affinity between ligand $X$ and its receptor, as well as additional factors.

Parameter $\beta$ is the maximal production level of $Y$. Maximal production is reached at high activator concentration, $X >> K$. Because at high concentration, $X$ binds the receptor with high probability to generate more products per unit time.

Parameter $n$ is known as Hill coefficient. It governs the steepness of the curve between two inflection points of the input function. Usually, it is moderately steep, with $n = 1 – 4$. The larger is $n$, the more step-like being the input function. Particularly, When $n$ =1, hill function is equal to Michaelis Menten equation. As many functions in biology, the Hill function approaches a limiting value at a high level of $X$, rather than increase indefinitely.

For a repressor, the Hill function is a decreasing S-shaped curve, whose shape depends on three similar parameters:

$$f(x) = \frac{\beta}{1+\left(\frac{X^*}{K}\right)^n}$$ Hill input function for repressor (3)

The production of $Y$ is balanced by two processes, degradation (destruction by specific proteins in the cell) and dilution (the reduction in concentration due to the increase of cell volume during growth). The degradation rate is $\alpha_{\text{deg}}$, and the dilution rate is $\alpha_{\text{dil}}$, giving a total degradation plus dilution rate (in units of 1/time) of

$$\alpha = \alpha_{\text{deg}} + \alpha_{\text{dil}} \qquad (4)$$

The change in the concentration of $Y$ due to the difference between its production and degradation plus dilution, as described by a dynamic equation:

$$dY/dt = f(X^*) – \alpha Y \qquad (5)$$

At stead state, $Y$ reaches a constant concentration $Y_{st}$, the steady-state concentration can be found by solving for $dY/dt = 0$. The steady-state concentration is:

$$Y_{st} = f(X^*)/\alpha \qquad (6)$$

If reached its maximal level, we can also write as:

$$Y_{st} = \beta/\alpha \qquad (7)$$

This makes sense: The higher is the production rate $\beta$, the higher will reach the steady-state concentration $Y_{st}$. The higher is the degradation/dilution rate α, the lower is $Y_{st}$.

Now let us consider one single locus with allele A and a, which are or code some kind of receptor and can be regulated by ligand $X$. The product of allele A is $Y_1$ at steady-state under concentration $[X_{11}^*]$ of active $X$ ($X_{11}^*$), and that of a is $Y_2$ under concentration $[X_{22}^*]$. The production function of two alleles is expressed respectively as:

$$\text{A: } dY_1/dt = f([X_{11}^*]) - \alpha_1 Y_1 \qquad (8a)$$

$$\text{a: } dY_2/dt = f([X_{22}^*]) - \alpha_2 Y_2 \quad (8b)$$

Where $\alpha_1 > 0$ and $\alpha_2 > 0$ are the relative degradation rate.

Then, the product of two alleles at steady-state is respectively:

$$\text{A: } Y_1 = f(X_1^*)/\alpha_1 = \frac{\beta_1}{\alpha_1}\frac{[X_{11}^*]^{n1}}{K_{11}^{n1} + [X_{11}^*]^{n1}} \quad (9a)$$

$$\text{a: } Y_2 = f(X_2^*)/\alpha_2 = \frac{\beta_2}{\alpha_2}\frac{[X_{22}^*]^{n2}}{K_{22}^{n2} + [X_{22}^*]^{n2}} \quad (9b)$$

With $\mu_j = \frac{\beta_j}{\alpha_j}$, then Equations 9a and 9c are transformed into:

$$\text{A: } Y_1 = \mu_1 \frac{[X_{11}^*]^{n1}}{K_{11}^{n1} + [X_{11}^*]^{n1}} \quad (10a)$$

$$\text{a: } Y_2 = \mu_2 \frac{[X_{22}^*]^{n2}}{K_{22}^{n2} + [X_{22}^*]^{n2}} \quad (10b)$$

Regarding the relationship between products of homozygotes (AA and aa) and heterozygote (Aa) of the locus, we consider three scenarios. The general assumption for three scenarios is that: (1) the ligand background concentration is consistent among two homozygotes (representing the parents) and heterozygote (representing the $F_1$ hybrid); so if two alleles share the same kind of ligand background, the ligand background concentration in homozygotes and heterozygote will be $[X_{11}^*]$ or $[X_{22}^*]$, with $[X_{11}^*] = [X_{22}^*]$; if two alleles have their respective ligand backgrounds, two homozygotes and the heterozygote will maintain the same concentration of both ligands, $[X_{11}^*]$ and $[X_{22}^*]$; (2) the ligand background can be equally and randomly allocated to two alleles in the homozygote, and the reaction of two alleles in heterozygote is independent [86] and the ligand background will be allocated to two different alleles under the rule as defined in different scenarios; (3) there is a basal product *m* in two homozygotes and heterozygote when there is no ligand.

Firstly, we consider the three scenarios under the situation that the ligand works as an activator.

Scenario 1: null allele vs one functional allele of one polymorphic site under one ligand background (**Fig. 3a**). The ligand background concentration in two homozygotes and heterozygote will be $2[X_{11}^*] = 2[X_{22}^*] = 2[X^*]$. The product of AA, aa and Aa at steady state will be:

$$\text{AA: } Y_{11} = m + \mu_1 \frac{[X^*]^{n1}}{K^{n1} + [X^*]^{n1}} + \mu_1 \frac{[X^*]^{n1}}{K^{n1} + [X^*]^{n1}} \tag{11a}$$

$$\text{aa: } Y_{22} = m + 0 \tag{11b}$$

$$\text{Aa: } Y_{12} = m + \mu_1 \frac{(2[X^*])^{n1}}{K_{11}^{n1} + (2[X^*])^{n1}} \tag{11c}$$

Scenario 2: two alleles of one polymorphic site under two independent backgrounds, that is, two alleles of one polymorphic site of the receptor can be bound by two respective and independent ligands as the backgrounds of the receptor (**Fig. 3b, Additional file 1: Figure S28-S29**). The ligand background concentration in two homozygotes and heterozygote will be 2[$X_{11}$*] and 2[$X_{22}$*], but $X_{11}$* can only be allocated to allele A and $X_{22}$* to allele a. The product of AA, aa and Aa at steady state will be:

$$\text{AA: } Y_{11} = m + \mu_1 \frac{[X_{11}^*]^{n1}}{K_{11}^{n1} + [X_{11}^*]^{n1}} + \mu_1 \frac{[X_{11}^*]^{n1}}{K_{11}^{n1} + [X_{11}^*]^{n1}} \tag{12a}$$

$$\text{aa: } Y_{22} = m + \mu_2 \frac{[X_{22}^*]^{n2}}{K_{22}^{n2} + [X_{22}^*]^{n2}} + \mu_2 \frac{[X_{22}^*]^{n2}}{K_{22}^{n2} + [X_{22}^*]^{n2}} \tag{12b}$$

$$\text{Aa: } Y_{12} = m + \mu_1 \frac{(2[X_{11}^*])^{n1}}{K_{11}^{n1} + (2[X_{11}^*])^{n1}} + \mu_2 \frac{(2[X_{22}^*])^{n2}}{K_{22}^{n2} + (2[X_{22}^*])^{n2}} \tag{12c}$$

Scenario 3: two alleles of one polymorphic site with shared background, that is, two alleles of one polymorphic site of the receptor can be bound by the same ligand as the background of the receptor (**Additional file 1: Figure S31**). The ligand background concentration in two homozygotes and heterozygote will be 2[$X_{11}$*] = 2[$X_{22}$*] = 2[$X$*]. If the ligand background $X$* was equally allocated to each of the two alleles in heterozygote as the simulation previously reported [86, 87], the product of AA, aa and Aa at steady state will be:

$$\text{AA: } Y_{11} = m + \mu_1 \frac{[X^*]^{n1}}{K_{11}^{n1} + [X^*]^{n1}} + \mu_1 \frac{[X^*]^{n1}}{K_{11}^{n1} + [X^*]^{n1}} \tag{13a}$$

$$\text{aa: } Y_{22} = m + \mu_2 \frac{[X^*]^{n2}}{K_{22}^{n2} + [X^*]^{n2}} + \mu_2 \frac{[X^*]^{n2}}{K_{22}^{n2} + [X^*]^{n2}} \tag{13b}$$

$$\text{Aa: } Y_{12} = m + \mu_1 \frac{[X^*]^{n1}}{K_{11}^{n1} + [X^*]^{n1}} + \mu_2 \frac{[X^*]^{n2}}{K_{22}^{n2} + [X^*]^{n2}} \quad \text{(Equal allocation)} \tag{13c}$$

As our simulation indicated, the locus will always appear to be additive under the situation of equal allocation ($X^* = (X_{11}^* + X_{22}^*)/2$). We proposed an optimal strategy to maximize the output of the heterozygote. Let $S_1+S_2 = 2[X^*]$, $S_1$ and $S_2$ represent the ligand concentration allocated to allele A and a in heterozygote, respectively, when the product of heterozygote $Y_{12}$ is maximized at the ligand concentration $2[X^*]$ **(Fig. 3c-d and Additional file 1: Figure S32-S33**). The product of Aa at steady state will be:

$$\text{Aa: } Y_{12} = m + \max\left(\mu_1 \frac{S_1^{n_1}}{K_{11}^{n_1} + S_1^{n_1}} + \mu_2 \frac{S_2^{n_2}}{K_{22}^{n_2} + S_2^{n_2}}\right) \text{(Maximized allocation)} \quad (13d)$$

Secondly, we consider the three scenarios under the situation that the ligand works as a repressor.

Regarding Scenario 1, null allele vs one functional allele of one polymorphic site under one ligand background (**Additional file 1: Figure S27**). The product of AA, aa and Aa at steady state for negative regulation will be:

$$\text{AA: } Y_{11} = m - \mu_1\left(1 - \frac{K^{n_1}}{K^{n_1} + [X^*]^{n_1}}\right) - \mu_1\left(1 - \frac{K^{n_1}}{K^{n_1} + [X^*]^{n_1}}\right) \quad (14a)$$

$$\text{aa: } Y_{22} = m - 0 \quad (14b)$$

$$\text{Aa: } Y_{12} = m - \mu_1\left(1 - \frac{K^{n_1}}{K^{n_1} + (2[X^*])^{n_1}}\right) \quad (14c)$$

Regarding Scenario 2, two alleles of one polymorphic site under two independent backgrounds (**Additional file 1: Figure S30**). The product of AA, aa and Aa at steady state for negative regulation will be:

$$\text{AA: } Y_{11} = m - \mu_1\left(1 - \frac{K_{11}^{n_1}}{K_{11}^{n_1} + [X_{11}^*]^{n_1}}\right) - \mu_1\left(1 - \frac{K_{11}^{n_1}}{K_{11}^{n_1} + [X_{11}^*]^{n_1}}\right) \quad (15a)$$

$$\text{aa: } Y_{22} = m - \mu_2\left(1 - \frac{K_{22}^{n_2}}{K_{22}^{n_2} + [X_{22}^*]^{n_2}}\right) - \mu_2\left(1 - \frac{K_{22}^{n_2}}{K_{22}^{n_2} + [X_{22}^*]^{n_2}}\right) \quad (15b)$$

$$\text{Aa: } Y_{12} = m - \mu_1\left(1 - \frac{K_{11}^{n_1}}{K_{11}^{n_1} + (2[X_{11}^*])^{n_1}}\right) - \mu_2\left(1 - \frac{(K_{22})^{n_2}}{K_{22}^{n_2} + (2[X_{22}^*])^{n_2}}\right) \quad (15c)$$

Regarding Scenario 3, two alleles of one polymorphic site with shared background (**Additional file 1: Figure S34-35**). The product of AA, aa and Aa at steady state for negative regulation will be:

$$\text{AA: } Y_{11} = m - \mu_1\left(1-\frac{K_{11}^{n1}}{K_{11}^{n1}+[X^*]^{n1}}\right) - \mu_1\left(1-\frac{K_{11}^{n1}}{K_{11}^{n1}+[X^*]^{n1}}\right) \quad (16a)$$

$$\text{aa: } Y_{22} = m - \mu_2\left(1-\frac{K_{22}^{n2}}{K_{22}^{n2}+[X^*]^{n2}}\right) - \mu_2\left(1-\frac{K_{22}^{n2}}{K_{22}^{n2}+[X^*]^{n2}}\right) \quad (16b)$$

$$\text{Aa: } Y_{12} = m - \mu_1\left(1-\frac{K_{11}^{n1}}{K_{11}^{n1}+[X^*]^{n1}}\right) - \mu_2\left(1-\frac{K_{22}^{n2}}{K_{22}^{n2}+[X^*]^{n2}}\right) \text{(Equal allocation)} \quad (16c)$$

Or,

$$\text{Aa: } Y_{12} = m - \max\left(\mu_1\left(1-\frac{K_{11}^{n1}}{K_{11}^{n1}+S_1^{n1}}\right) + \mu_2\left(1-\frac{K_{22}^{n2}}{K_{22}^{n2}+S_2^{n2}}\right)\right) \text{(Maximized allocation)} \quad (16d)$$

Where $S_1+S_2 = 2[X^*]$.

According the simulated values of $Y_{11}$,$Y_{12}$ and $Y_{22}$, we calculated the degree of dominance (d/a) for the locus as:

$$d/a = (Y_{12} - (Y_{11}+Y_{22})/2)/(|Y_{22}-Y_{11}|/2) \quad (17)$$

**Simulation2: the model reflecting the performance of homozygotes and heterozygote of one locus simulated according to trimer ABA assembly**

The balance between genes involved in a biological complex is one important hypothesis about heterosis. The typical example for gene balance was reported by Balazs and coleagues [43]. Their studies indicated that mutation of the subunit in a trimer ABA complex can result in imbalance and thus is harmful, which might impact gene imbalance on dominance. However, these studies did not consider the effects from the counterpart background. Thus, we simulated the effects of complex background on dominance of one single polymorphic locus that codes A or B.

In the system of trimer ABA complex, A and B are monomers, AB is the bridge dimer without active function, the trimer ABA is the functional entity. The reaction among monomers, dimer and trimer could be illustrated by the following chemical formula:

A + B <=> AB (18)

AB + A <=> ABA (19)

For simplicity, we consider a pseudo equilibrium state, that is: A and B were input once in an enclosed environment and no degradation was considered; after a period of time, a chemical equilibrium state will be achieved. Set $S_A$ and $S_B$ as the initial input concentration of A and B, $k_{AB}$ as the association rate from left to right in formula (18), $m_{AB}$ as the dissociation rate from right to left of formula (18), $k_{ABA}$ and $m_{ABA}$ as the association rate from left to right and the dissociation rate from right to left in formula (19). And let $[A]$, $[B]$, $[AB]$ and $[ABA]$ represent the concentration of A, B, AB and ABA at equilibrium state. So we have:

$$S_A = [A] + [AB] + 2[ABA]$$

$$S_B = [B] + [AB] + [ABA]$$

$$k_{AB}\times[A][B] = m_{AB}\times[AB]$$

$$[AB] = k_{AB}/m_{AB}\times[A][B]$$

$$k_{ABA}\times[AB][A] = m_{ABA}\times[ABA]$$

$$[ABA] = k_{ABA}/\ m_{ABA}\times[AB][A]$$

$$[ABA] = k_{ABA}/\ m_{ABA}\times k_{AB}/m_{AB}[A][B][A]$$

We define association coefficient by the ratio of association to dissociation for two steps as:

$$K_1 = k_{AB}/m_{AB}$$

$$K_2 = k_{ABA}/m_{ABA}$$

then, we derived that

$$[AB] = K_1\times[A][B]$$

$$[ABA] = K_1\times K_2\times[A][B][A]$$

According to the formula of stoichiometry balance, we can have:

$$S_A = [A] + K_1\times[A][B] + 2K_1\times K_2\times[A][B][A]$$

$$S_B = [B] + K_1\times[A][B] + K_1\times K_2\times[A][B][A]$$

Through the above equation, we get

$$[B] = S_B/(1+ K_1\times[A]+ K_1\times K_2\times[A]^2)$$

Then we introduced $[B]$ into $S_A$, we get

$$S_A = [A] + S_B\times K_1\times[A]/(1+ K_1\times[A]+ K_1\times K_2\times[A]^2)+$$
$$2S_B\times K_1\times K_2\times[A]^2/(1+ K_1\times[A]+ K_1\times K_2\times[A]^2)$$

We set a target function $f([A])$,

$$f([A]) = (S_A - ([A] + S_B \times K_1 \times [A]/(1+ K_1 \times [A]+ K_1 \times K_2 \times [A]^2)+$$
$$2S_B \times K_1 \times K_2 \times [A]^2/(1+ K_1 \times [A]+ K_1 \times K_2 \times [A]^2))^2$$

Among of the function of $f([A])$, only $[A]$ is the unknown parameters, $S_A$, $S_B$, $K_1$ and $K_2$ were all the predefined data, thus the value of $[A]$ that minimizes $f([A])$ is the solution of the concentration of $A$ at equilibrium state. Once the concentration of $A$ at equilibrium state is obtained, the concentration of B, AB, and ABA at equilibrium state could be easily calculated according to the above equations.

For given $S_A$, $S_B$, $K_1$ and $K_2$, we solve the equation by using the optimize function in R and get the concentration of A, B, AB and ABA at the equilibrium state, the solutions of parent and $F_1$ was follow the same equations described above (**Additional file 1: Figure S51**).

We simulated two scenarios as following:

Scenario1, keep the input concentration of B fixed and constant among two homozygotes and the heterozygote of A, and A was coded by one polymorphic locus (**Additional file 1: Figure S51b-c**): $S_A$ ranges from 0 to 20 nmol/L, with $S_B$ = 2.5 nmol/L, $K_1$ = 1, $K_2$ = 100. The simulated data for the genotype of AA, aa and Aa as follow:

AA: $S_{A(AA)}$ = 0 - 20 nmol/L, $S_{B(AA)}$ = 2.5 nmol/L

aa: $S_{A(aa)}$ = 0 - 20 nmol/L, $S_{B(aa)}$ = 2.5 nmol/L

Aa: $S_{A(Aa)} = (S_{A(AA)} + S_{A(aa)})/2$, $S_{B(Aa)}$ = 2.5 nmol/L

Scenario2, keep the input concentration of A fixed and constant among two homozygotes and the heterozygote of B, and B was coded by one polymorphic locus (**Additional file 1: Figure S51d-e**): $S_B$ ranges from 0 to 20 nmol/L, with $S_A$ = 5 nmol/L, $K_1$ = 1, $K_2$ = 100. The simulated data for the genotype of AA, aa and Aa as follow:

BB: $S_{A(BB)}$ = 5 nmol/L, $S_{B(BB)}$ =0 - 20 nmol/L

bb: $S_{A(bb)}$ = 5 nmol/L, $S_{B(bb)}$ = 0 - 20 nmol/L

Bb: $S_{A(Bb)}$ =5 nmol/L, $S_{B(Bb)} = (S_{A(BB)} + S_{A(bb)})/2$

Same as the above simulation according to Hill function, according the simulated values of $Y_{11}$ (AA or BB), $Y_{12}$ (Aa or Bb) and $Y_{22}$ (aa or bb), we calculated the degree of dominance (d/a) for the locus as:

$$d/a = (Y_{12} - (Y_{11} + Y_{22})/2)/(|Y_{22} - Y_{11}|/2)$$

## References


84. Alon U: *An Introduction to Systems Biology: Design Principles of Biological Circuits.* 2006.

85. Omholt SW, Plahte E, Oyehaug L, Xiang K: Gene regulatory networks generating the phenomena of additivity, dominance and epistasis. *Genetics.* 2000;155:969-980.

86. Veitia RA: A generalized model of gene dosage and dominant negative effects in macromolecular complexes. *FASEB J.* 2010;24:994-1002.

87. Papp B, Pal C, Hurst LD: Dosage sensitivity and the evolution of gene families in yeast. *Nature.* 2003;424:194-197.
