# Supplementary material for: Large-scale genomic and transcriptomic profiles of rice hybrids reveal a core mechanism underlying heterosis
Source: Genome Biol. 2022 Dec 22;23:264. doi: 10.1186/s13059-022-02822-8 (PMC9773586; doi:10.1186/s13059-022-02822-8)
Supplement: Supplementary file 4 — Additional file 4. Review history. [file 13059_2022_2822_MOESM4_ESM.docx]

Review History

**First round of review**

**Reviewer 1**

**Were you able to assess all statistics in the manuscript, including the appropriateness of statistical tests used?**

Yes, and I have assessed the statistics in my report.

**Comments to author:**

The authors studied the heterosis using GWAS, transcriptomic profiles, simulation analyses and experiment validation. They used 418 rice hybrids that crossed by two testers and a collection of inbred lines to explore the genetic architecture of yield and component traits in hybrids and heterosis. They found that overdominant/dominant QTLs are generally correlated to heterosis and contributed to the sensitivity of environmental changes. They also performed the transcriptomic profiles on Liangyoupei 9 hybrid-parent trios across three tissues and found a considerable number of genes exhibited dominance and overdominance expression pattern. Based on theoretical hypothesis, extensive simulations, they propose a molecular model for interpreting heterosis, HoIIB, which were discussed and validated by gene expression experiment in yeast. The study presents an extensive work with large-scale data collection and analyses. The proposed model is interesting and may be helpful for further our understanding of heterosis formation. I have several concerns that may improve the manuscript quality.

Line 1-2, I suggest it's better if the title may be discreet.

Line 20, the genetic should be genetics or genetic mechanism.

Line 99-100, I have no idea what the non-additive phenomena and additive phenomena are. If the author indicates heterosis as non-additive phenomena, what is additive phenomena?

Line 103, I can't see a smooth logic between background-dependent heterosis and the proposed model. The authors need more clarifications on homo-insufficiency and insufficient background.

Line 110-111, how did the authors infer the clusters to subspecies based different sets of marker sets? 4.6M SNPs on 267 parents but only 100K SNPs on 3K rice varieties.

Line 128-129, I don't understand how the authors calculate the significance on correlation difference between parent and hybrids (fig. s4a). There is only single value for each group.

Line 140-142, The conclusion the authors made is not rigorous. Just from the present study, two of 6 traits did not follow this trend. Although the data from 2808 maize hybrids on five environments did so, it only involved three traits, flowering time, plant height and ear weight. The authors should use more comprehensive data to analyze this. As I known, a previous publication on ~1000 F1 in 11 environment for 12 traits has analyzed the similar issues (Liu et al, 2020, Mol Biol Evol). They found, different to heterosis on genetic value, the maize F1s show more diverse on heterosis in plasticity. That is, some traits are more responsive in F1 that parents (including flowering, plant height and ear weight), but others are not.

Line 145-146, I don't think the fig. S6 show the evidence about heterozygosity, or not very directly.

Line 150-152, The fig. 1 did not show the proportion of middle parent heterosis, did the authors mean the the RBP that indicates the F1 phenotype ranged between parents?

Line 163-164, The comparison requires the statistical test for significance on the difference. May be a x2 statistic can be tested.

Line 192-195, The conclusion is not directly relevant to the results.

Line 196, If the non-additive directly indicates the dominance, please use dominance here. The notion of non-additive may also include the epistasis.

Line 239, Please specify the definition of genetic background. Did the authors mean the different cross combinations? Or the different genotypes at one QTL locus? Or different tester parents (Nip or 9311)? The more details should be helpful for understanding the results and the conclusion.

Line 240-242, I can't see the conclusion from fig. S23, maybe I'm wrong, please correct me if so. For example, in the case of three traits (SPP, KGW, PNP) in the combination of Jx9311 in Changsha, the QTL from A, OD, POD did not show much different proportion of accumulated variation coefficient or very similar. By the way, how did the authors calculate the accumulated variation coefficient? It wasn't mentioned.

Line 244, In this case of IF2 population, I still can't see the difference between the QTL types on phenotypic variations (cv). Maybe I don't understand, please clarify here in more details, by how to carry out the calculation and comparisons. I recommend using the statistical analysis instead of descriptive statements.

Line 249-250, What's the definition of non-additive phenomenon at phenotype level? If the authors indicate the over-parent phenomenon, at phenotype level, please be more specific.

Line 264, The result of fig. 2b has no relevant to background dependence.

Line 274-276, maybe the POD or NOD genes are more likely to be regulated by TF than additive genes, if the enrichment exists, it's reasonable for drawing the author's conclusion.

However, I wonder it is possible to integrate QTL results of hybrid population and gene expression of a hybrid for a more cohesive conclusion. For example, what's the expression pattern of genes located within different types of QTLs, if which QTLs are polymorphic in the parents between PA64S and 9311.

Line 370-372, Did the authors can link the three levels of transcription, QTL and traits for the HoIIB model to make a cohesive explanation of molecular mechanisms of heterosis? We often discuss heterosis at the phenotype level, which is more direct in breeding, most explanations are at the genetic levels including the three theories. We are indeed curious the molecular mechanisms of heterosis of observed phenotype such as yield.

Line 375-377, I think this phenomenon for gene expression and traits between F1 and parents is very reasonable, that is, the smaller difference existed between two parents at over dominant QTL or gene expression. It's not surprising, because the definition or the calculation formula of d/a should determine the observed results. If the d/a goes larger, the a (the average of parental difference) should be smaller.

Line 396-397, How did the authors define the gene function or not at the level of gene expression?

Line 438-440, The experiment, and result are interesting! it seems to me that SSU1 depends on the expression dosage of FZF1. The dosage effect is also a hypothesis for single-gene overdominance phenomenon, that high or low expression may not be the optimal for phenotype. In this case, I wonder what if the native expression of FZF1 is reduced by RNAi or crispr. Did the authors test the level of native expression of FZF1 in the lines of SSU1/SSU1, SSU1/ssu1 and ssu1/ssu1? If the case is distinct to the (optimal) dosage hypothesis, it should be more amazing.

Line 499-450, The authors provided multiple evidence and simulations that proved the HoIIB phenomenon may exist for heterosis. But why it will happen? That is, why insufficient background (gene expression or phenotype) can make hybrid superior to parents. For example, why low FZF1 expression result in highest SSU1/ssu1 expression, the increase of FZF1 expression can increase SSU1/SSU1 expression faster than SSU1/ssu1? May the authors provide more explanations on how the HoIIB phenomenon happen biologically. It will be useful to broad readers.

**Reviewer 2**

**Were you able to assess all statistics in the manuscript, including the appropriateness of statistical tests used?**

Yes, and I have assessed the statistics in my report.

**Comments to author:**

In the manuscript under review, the authors proposed a model (HoIIM) explaining the mechanism of additive and non-additive genetic effects. From the theorical point of view, the model can be viewed as a different version of the one in Omholt et al. 2000, which used the Hill equation to quantify the ligand-receptor binding. One of the three scenarios of the HoIIM model was experimentally validated in yeast by the authors. Furthermore, they claimed that the genetic basis of heterosis can also be explained by the HoIIM model. To provide evidence, field trials were conducted for 265 rice varieties and their single-cross hybrids with two testers. GWAS was performed based on re-sequenced genomic data.

I think the HoIIB model contains very nice idea and many results in this manuscript are interesting. However, I detected some major problems in the data analysis and the argumentation line of the HoIIB model, which were listed below:

1. The authors ignored the genetic expectations of the QTL effects being tested in their GWAS models, which could lead to incorrect interpretations of the results. As the authors explained, GWAS was performed based on the two subpopulations of parental lines (Indica and Japonica), and on the four subpopulations of hybrids (Indica/Japonica x 9311/Nipponbare) respectively. And in the latter case, the hybrid performance and mid-parent values were used as the response variable respectively (lines 172-176). Based on quantitative genetics theory, it is easy to see that in the GWAS with parental lines, the genetic expectation of the QTL effect (P_-_QTL) is the additive effect, in the GWAS with test-cross hybrids using the mid-parent heterosis as the response, the genetic expectation of the QTL effect (Hmp_-_QTL) is the dominance effect (assuming absence of epistasis), while in the GWAS with test-cross hybrids using the F1 performance as the response, the genetic expectation of the QTL effect (F1_-_QTL) is  *d−a* or *d+a* (depending on whether the tester genotype is superior or inferior). Thus, the three sets of QTL (P_-_QTL, F1_-_QTL and Hmp_-_QTL) each has specific genetic effects. However, this fact was completely ignored in the comparison of results (lines 186-188). And because of this, some incorrect interpretations were made by the authors. Please see the next point.

2. In the section “Non-additive, which is more variable than additive, is the main contributor to heterosis”, the authors focused on mid-parent heterosis (lines 197-199). But actually, this is a trivial statement as additive effects do not contribute to mid-parent heterosis at all, simply from the definition of additive and dominance effects. And this is true regardless whether epistasis is present or not. An easy two-locus example: 𝐺𝑝1 = 𝑎1 + 𝑎2 + 𝑎𝑎12 , 𝐺𝑝2 = −𝑎1 − 𝑎2 + 𝑎𝑎12, 𝐺𝐹1 = 𝑑1 + 𝑑2 + 𝑑𝑑12, then 𝑀𝑃𝐻 = 𝐺𝐹1 − 1 2 (𝐺𝑝1 + 𝐺𝑝2) = 𝑑1 + 𝑑2 + 𝑑𝑑12 − 𝑎𝑎12. We can easily see that the additive effects cancelled out. From the authors’ argumentation line, I realized that they didn’t noticed the type of genetic effects represented by the three sets of QTL (P_-_QTL, F1_-_QTL and Hmp_-_QTL) are different. For example, the authors re-estimated the additive and dominance effects for all three sets of QTL and observed that F1_-_QTL and Hmp_-_QTL showed more non-additive pattern than P_-_QTL (lines 203-206). Indeed, this is expected because a P_-_QTL must have significant additive effects and an Hmp_-_QTL must have significant dominance effects. For an F1_-_QTL, the *d−a* or  *d+a* effect is significant. Because both additive and dominance is involved, it is natural that it is more likely to show a non-additive pattern.

3. The HoIIB model provide an interesting explanation of the additive and non-additive pattern at the level of gene expression, but the bridge between non-additive gene expression and non-additive genetic effects on the phenotype (hence on heterosis) is missing. The theory of the HoIIB model presented in Additional File 3 follows the idea of the interpretation of additive, dominance and epistasis with gene regulatory networks (Omholt et al. 2000). The experimental validation in yeast verified Scenario 1, where one null-allele and one functional allele is presented at a polymorphic site (But the other two scenarios were not experimentally validated). In the validation, the ligand (input of the Hill equation, which is the core of the HoIIB model) is the expression level of transcription factor (FZF1), and the receptor (output of the Hill equation) is the expression level of the target gene (SSU1). Thus, the validation is convincing for the non-additive pattern at the level of gene expression. However, the authors proposed that the HoIIB model is able to explain the non-additive effects for the heterosis in phenotypes. This has not been experimentally validated yet. Of course, a direct validation would much more difficult than the level of gene expressions, as one first has to identify the potential ligand factors (the input of the Hill equation). Based on the results already obtained by the authors, a possible indirect way is to build up a link between the non-additive pattern in the gene expression and the non-additive effects on phenotypes. Unfortunately, this link is also missing in the current study. The authors did try to establish the link, but using a single-cross hybrid 9311 x PA64S (subsection “Non-additive expressed genes are highly dependent on the expression of their upstream transcription factors”) rather than using the same hybrid population for GWAS. This is in my opinion not convincing because both the genes which are differentially expressed and the QTL effects may vary in different bi-parental populations. Thus, the expression pattern of genes observed in this specific hybrid may not necessarily be the same as in the four test-cross hybrid populations. Ideally, the gene expressions should be taken from the same population whose phenotype was evaluated. For example, if one observes that the mid-parent heterosis at the level of gene expression is positively correlated with the mid-parent heterosis at the level of phenotype, then it is much more convincing.

4. There are ambiguous interpretations on the “observed evidence” for the HoIIB model. For example, in the section “The HoIIB model was supported by different levels of evidence”, it was stated that “homo-insufficient expression was substantially observed in the higher parent for genes with dominant and overdominant transcription, compared to those with additive transcription” (lines 375-377). Here, unlike the experimental validation in yeast, the authors did not distinguish the transcript factors and the target genes. If I understood correctly, the additive or non-additive pattern and the homo-insufficient phenomenon was based on the expression of the same gene. Therefore, implicitly, the authors switched to the scenario in which the ligand and receptor are in a “dynamic balance” (meaning that in the Hill equation the input and output are NOT independent). This is different from the basic assumption of the HoIIB model “the ligand background concentration keeps constant among two homozygotes and the heterozygote” (Additional File 3, lines 66-67). Actually, the “dynamic balance” case has already been treated in Omholt et al. (2000). If this is what the authors meant to argue, then it is correct, but not novel and not new. The same ambiguous arguments occurred in lines 381-383, where the authors meant that the observed low phenotype of a parent served as an evidence of homo-insufficiency of the background of the non-additive pattern of the phenotype in the parent-hybrid combination.

To summarize, I think part of results in the manuscript is very interesting for researchers in the relevant area. The theory and experimental validation of the HoIIB model at the level of gene expression is convincing. The heterosis experimental data and GWAS results may also be publishable. But the whole story that the authors intended to tell, i.e., the HoIIB model explains the genetic basis of heterosis on phenotypic traits, is not convincing in several aspects. I am afraid I cannot recommend its publication in Genome Biology.

**Authors Response**

**Point-by-point responses to the reviewers’ comments:**

Reviewer #1:

The authors studied the heterosis using GWAS, transcriptomic profiles, simulation analyses and experiment validation. They used 418 rice hybrids that crossed by two testers and a collection of inbred lines to explore the genetic architecture of yield and component traits in hybrids and heterosis. They found that overdominant/dominant QTLs are generally correlated to heterosis and contributed to the sensitivity of environmental changes. They also performed the transcriptomic profiles on Liangyoupei 9 hybrid-parent trios across three tissues and found a considerable number of genes exhibited dominance and overdominance expression pattern. Based on theoretical hypothesis, extensive simulations, they propose a molecular model for interpreting heterosis, HoIIB, which were discussed and validated by gene expression experiment in yeast. The study presents an extensive work with large-scale data collection and analyses. The proposed model is interesting and may be helpful for further our understanding of heterosis formation. I have several concerns that may improve the manuscript quality.

Line 1-2, I suggest it's better if the title may be discreet.

*Reply: As your suggestion, we changed the title as “Large scale genomic and transcriptomic profiles of rice hybrids revealed a core mechanism underlying yield heterosis”.*

Line 20, the genetic should be genetics or genetic mechanism.

*Reply: Thank you for your corrections. We changed it into “genetic mechanism”.*

Line 99-100, I have no idea what the non-additive phenomena and additive phenomena are. If the author indicates heterosis as non-additive phenomena, what is additive phenomena?

*Reply: Sorry for that we did not describe the term clearly. Here we use nonadditive phenomena to outline the phenomenon that F1 biases from the mean performance of its two parents at the phenotype, QTL and transcription levels. So mid-parent heterosis is one nonadditive phenomenon. We described the term “nonadditive phenomenon” in the revised manuscript (line 102-107 in revised version). Of course, as you mentioned later, nonadditive may include dominant, overdominant and epistasis in genetics. We will carefully use this term when it refers to the specific mean of “dominant and overdominant”.*

Line 103, I can't see a smooth logic between background-dependent heterosis and the proposed model. The authors need more clarifications on homo-insufficiency and insufficient background .

*Reply: Indeed, as you had pointed, the logic is not smooth in the concerned line. In the revised manuscript we added some simple explanation to the model (line 107-114 in revised version).*

Line 110-111, how did the authors infer the clusters to subspecies based different sets of marker sets? 4.6M SNPs on 267 parents but only 100K SNPs on 3K rice varieties.

*Reply: Sorry for that we did not describe it clearly. We inferred the clusters according to 4.6M SNPs on 267 parents, and we compared them with the clusters that published by Wang et al. (Wang et al, Genomic variation in 3,010 diverse accessions of Asian cultivated rice, 2018, nature), where 100K SNPs on 3K rice varieties were used. But we did not used its information finally, so we removed the content in the revised version (line 119-122 in revised version).*

Line 128-129, I don't understand how the authors calculate the significance on correlation difference between parent and hybrids (fig. s4a). There is only single value for each group.

*Reply: I'm sorry that the statistical test was not clearly described in the previous submitted manuscript. In the revised version, we do the statistical tests like this: in order to obtain the P values, we first transformed the correlation coefficient of parental phenotype between the two environments, and the correlation coefficient of hybrid phenotype between the two environments into z-scores, then calculate the statistical test from the obtained z-score. The specific comparisons are: indica parents vs indica × Nipponbare F1, indica parents vs indica × 9311 F1, japonica parents vs japonica × Nipponbare F1 and japonica parents vs japonica × 9311 F1 (Figure S4a). The statistical test of phenotypic correlation coefficient of maize is similar (Figure S5a-c).*

Line 140-142, The conclusion the authors made is not rigorous. Just from the present study, two of 6 traits did not follow this trend. Although the data from 2808 maize hybrids on five environments did so, it only involved three traits, flowering time, plant height and ear weight. The authors should use more comprehensive data to analyze this. As I known, a previous publication on ~1000 F1 in 11 environment for 12 traits has analyzed the similar issues (Liu et al, 2020, Mol Biol Evol). They found, different to heterosis on genetic value, the maize hybrids show more diverse on heterosis in plasticity. That is, some traits are more responsive in F1 that parents (including flowering, plant height and ear weight), but others are not.

*Reply: This is an important and fundamental concern. In fact, we had attempted to analyze the original phenotypic data from the paper of “Liu et al, Phenotypic Plasticity Contributes to Maize Adaptation and Heterosis. 2020, Mol Biol Evol”, however, the paper did not provide the original phenotypic data. But we checked the results of this paper, and found that 8 of 12 traits exhibit higher plasticity in hybrids compared with that in the maternal inbred lines. In addition, we obtained 11 phenotypes of parents and 265×1 hybrids in 4 environments from another maize population (Additional file 1: Figure S5e in the revised version) (Sherry et al, Heterosis Is Prevalent for Multiple Traits in Diverse Maize Germplasm. 2009, Plos One), and the yield performance of inbred lines and 120×15 wheat hybrids in 11 environments (Additional file 1: Figure S5f in revised version) (Zhao et al, Genome-based establishment of a high-yielding heterotic pattern for hybrid wheat breeding. 2015, PNAS). All the results show the similar phenomenon, that is, hybrids are more sensitive to the environment than that of inbred lines in most cases. Thus, the phenotypic data obtained from rice, maize and wheat indicated that it is a predominant phenomenon that hybrids are more sensitive to the environment than their inbred parents across species.*

Line 145-146, I don't think the fig. S6 show the evidence about heterozygosity, or not very directly.

*Reply: Sorry for the missing conclusion. Figure S6 does not directly reflect the effect of heterozygosity on heterosis. To avoid ambiguity, we removed the word “heterozygosity” in the revised version of line 157.*

Line 150-152, The fig. 1 did not show the proportion of middle parents heterosis, did the authors mean the REP that indicates the F1 phenotype ranged between parents?

*Reply: Fig.1c-d shows the degree of middle parent heterosis value (dHMP) which calculated as (F*_1_*-(P_1_ +P_2_)/2)/((P_1_ +P*_2_*)/2))*100. As you pointed, the RBP (range between the two parents) means that the F1 phenotype ranged between the two parents. It was described in Additional file: Figure S6 and in section “Phenotyping of the parental varieties and their F1 population”.*

Line 163-164, The comparison requires the statistical test for significance on the difference. May be a x2 statistic can be tested.

*Reply: Thank you for your suggestion. In the revised version, we conducted chi square test. The significance of the difference between the two environments was marked with black asterisk, and that between intersubspecific and intrasubspecific combination was marked with bright blue asterisk (Additional file 1: Figure S6). The specific comparisons between inter- and intrasubspecific combinations were J×Nip vs J×9311 and I×Nip vs I×9311.*

Line 192-195, The conclusion is not directly relevant to the results.

*Reply: Yes, as you pointed out, the conclusion is not direct. We changed the conclusion as: These results implied that the improvement of hybrids should concern both heterosis and the genetic background of the inbred lines, but their respective contribution varied depending on the combinations, environments and traits. (line 205-207 in the revised version).*

Line 196, If the non-additive directly indicates the dominance, please use dominance here. The notion of non-additive may also include the epistasis.

*Reply: We did not estimate the epistasis in current research. So the nonadditive indicates the dominance (including partial-dominance) and overdominance. We agree that it can not exclude the epistasis if we simply say nonadditive. So we defined the scope of the term “nonadditive” in line 102-106 of the revised manuscript.*

Line 239, Please specify the definition of genetic background. Did the authors mean the different cross combinations? Or the different genotypes at one QTL locus? Or different tester parents (Nip or 9311)? The more details should be helpful for understanding the results and the conclusion.

*Reply: Here, we refer the genetic background as different cross combinations. It has been noted in the revised manuscript (line 205-206 in the revised version). But in most cases of our manuscript, we refer the background as all factors that directly or indirectly affect the performance or function of the considered gene. They can be the transcription factors of one considered gene, or can be any regulation factor along the regulation pathway of the considered gene. So, when we say different (genetic) backgrounds, it can be different combinations with different genotypes that regulate the considered gene; it can also be different expression levels or function magnitude of the same genotype that regulate the considered gene but was induced by different environments (including the biological or physiological condition, such as different tissues).*

Line 240-242, I can't see the conclusion from fig. S23, maybe I'm wrong, please correct me if so. For example, in the case of three traits (SPP, KGW, PNP) in the combination of Jx9311 in Changsha, the QTL from A, OD, POD did not show much different proportion of accumulated variation coefficient or very similar. By the way, how did the authors calculate the accumulated variation coefficient? It wasn't mentioned.

*Reply: It should be admitted that our observed trend is a global phenomenon, since the real situation is complex. From the overall results we obtained, in most cases (28/32), the cumulative phenotypic coefficient of variation of dominant and overdominant QTLs, especially overdominant QTLs (15/16), was larger than that of additive QTLs, only few cases were not consistent with the trend. By the way, the MCC panel we used in this study is a natural population, the proportion of additive, dominant and overdominant of QTLs was varied largely and the allele frequency of each genotype in the QTL was not balanced, it is normal that part of combinations or traits did not show the expected trend (Additional file 1: Figure S23). In fact, when we examine the phenotypic coefficient of variation of QTLs identified in an immortalized F2 population, in which the frequency of each genotype was more balanced, the phenomenon is more consistent across different environments and traits (7/8 are in line with the general trend) (Additional file 1: Figure S24).*

*As you noted, the method that we calculate the cumulative coefficient of variation was lack in last manuscript. In the revised version, we added the description of the method for calculating the cumulative coefficient of variation to the Maretials and Methods section (line 808-820 in the revised version).*

Line 244, In this case of IF2 population, I still can't see the difference between the QTL types on phenotypic variations (cv). Maybe I don't understand, please clarify here in more details, by how to carry out the calculation and comparisons. I recommend using the statistical analysis instead of descriptive statements.

*Reply: As you had concerned in previous section, the difference of cumulative phenotypic cv between additive, dominant and super dominant is not strong. The reason is simple, because we used the same set of phenotypic data to estimate the phenotypic cv of different types of QTLs (additive, dominant and overdominant). However, from the results we obtained in immortalized F2 population (as an ideal population with balanced genotype frequency), seven eighths (7/8) are consistent with the general trend, thus, dominant and overdominant phenomenon are more sensitive to the genetic background than that of additive ones could be recognized as a predominant phenomenon. By the way, the statistical method for the calculation of phenotypic coefficient of variation was supplemented in the Maretials and Methods section in the revised version (line 808-820 in the revised version).*

Line 249-250, What's the definition of non-additive phenomenon at phenotype level? If the authors indicate the over-parent phenomenon, at phenotype level, please be more specific.

*Reply: As described above, nonadditive phenomenon at phenotype level represents heterosis. So we removed the content “no-additive phenomenon” in the revised manuscript (line 259-260).*

Line 264, The result of fig. 2b has no relevant to background dependence.

*Reply: This figure describes the consistency of expression patterns among different tissues, which was kind of background as mentioned above. In most cases of our manuscript refer the background as all factors that directly or indirectly affect the performance or function of the considered gene. They can be the transcription factors of one considered gene, or can be any regulation factor along the regulation pathway of the considered gene. So, when we say different (genetic) backgrounds, it can be different combinations with different genotypes that regulate the considered gene; it can also be different expression levels or function magnitude of the same genotype that regulate the considered gene but was induced by different environments (including the biological or physiological condition, such as different tissues).*

Line 274-276, maybe the POD or NOD genes are more likely to be regulated by TF than additive genes, if the enrichment exists, it's reasonable for drawing the author's conclusion. However, I wonder it is possible to integrate QTL results of hybrid population and gene expression of a hybrid for a more cohesive conclusion. For example, what's the expression pattern of genes located within different types of QTLs, if which QTLs are polymorphic in the parents between PA64S and 9311.

*Reply: As you guessed, TF can be enriched in some of the cases when we analyzed the transcription data of rice and Arobidopsis. And as you suggested, we tried to integrate QTL results of hybrid population and gene expression of hybrid LYP9 and its parents PA64S and 9311 (Figure S52). It indicated when comparing the frequently detected additive QTLs vs overdominant QTLs affecting yield traits, we observed that the candidate genes within the additive QTLs displayed distinctly lower expression, compared to those within the overdominant ones.*

Line 370-372, Did the authors can link the three levels of transcription, QTL and traits for the HoIIB model to make a cohesive explanation of molecular mechanisms of heterosis? We often discuss heterosis at the phenotype level, which is more direct in breeding, most explanations are at the genetic levels including the three theories. We are indeed curious the molecular mechanisms of heterosis of observed phenotype such as yield.

*Reply: Yes, we are all indeed curious the molecular mechanisms of heterosis of observed phenotype such as yield. And you put forward one wonderful suggestion, linking three levels (transcription, QTL and traits) for the HoIIB model to make a cohesive explanation of molecular mechanisms of heterosis. That is, how does the molecular performance of one gene show certain genetic performance, and then phenotypic performance? Our HoIIB model can do.*

*We suppose there is one TF who can activate its target gene that involves the development of rice yield trait. In a cross combination, the target gene shows polymorphic variation, two of the alleles being nominated by a1 and a2, and TF can bind and activate a1 but not a2 for simplicity. Let us consider two situations.*

*Situation 1: the expression level of TF is very high enough to activate two copies of allele a1 in parent with genotype a1a1, then one copy of allele a1 in F1 with genotype a1a2 can be completely activated; if the effect of this kind of molecular performance does not be disturbed by other factors, the target gene will show additive effect in genetics, and then this combination will not show heterosis at the phenotype level.*

*Situation 2: the expression level of TF is low and only enough to activate one of two allele a1 copies in parent with genotype a1a1, then one copy of allele a1 in F1 with genotype a1a2 can also be completely activated; if the effect of this kind of molecular performance does not be disturbed by other factors, the target gene will show dominant effect in genetics, and then this combination will show heterosis at the phenotype level.*

*These two situations show the molecular mechanism of heterosis being illustrated by the HoIIB model. The HoIIB model insists: (1) dominance and heterosis are not the properties of the target gene; (2) the intrinsic mechanism of heterosis and dominance is that the insufficient background results in insufficient function of homozygote but relatively or even completely sufficient function of heterozygote in molecular performance, then the relevant gene or QTL shows dominance or even over-dominance in genetics, and finally the relevant combination shows heterosis in phenotype. Besides TF vs its target, the molecular performance can be extent to any action between biological molecules, such as kinase vs its target, small RNA vs its target, signaling molecule vs its target, and so on. Such kind of description has been added in the discussion section (line 541-544 in the revised version).*

Line 375-377, I think this phenomenon for gene expression and traits between F1 and parents is very reasonable, that is, the smaller difference existed between two parents at over dominant QTL or gene expression. It's not surprising, because the definition or the calculation formula of d/a should determine the observed results. If the d/a goes larger, the a (the average of parental difference) should be smaller.

*Reply: Yes, you pointed out one predominant phenomenon, that is, the additive effect (a) is usually smaller in the situation with higher degree of dominance due to the definition or the calculation formula of d/a. It is a fact and it is also a rational fact from the aspect of HoIIB model. According to HoIIB model, insufficient background will decrease the performance of homozygote with higher function, and so lower down the parental difference, and thus we usually estimate relatively smaller additive effect (a) when there is apparent heterosis and dominant effect. This fact also supports one of our opinions, that is, the use of heterosis or dominance effect of one gene is the secondary solution under insufficient background, theoretically, the optimal solution is to improve the background to use the full function of the target gene.*

Line 396-397, How did the authors define the gene function or not at the level of gene expression?

*Reply: We defined it in the legend of Fig 4, but we are so sorry that the legends of Fig 4c and Fig 4d are reverse in last version of manuscript. We corrected them in the revised manuscript, and in Fig 4c: it was defined as 9311 null when the gene does not express in all young panicles of 1 mm, 2 mm and 3 mm of 9311, but expresses in all young panicles of 1 mm, 2 mm and 3 mm of PA64s; and it was defined as PA64s null in reverse; and it was defined as both functional when the gene expresses in all detected young panicles of 9311 and PA64s. For Col×C24 combinations in Arabidopsis, we used the same criterion (Additional file 1: Figure S42).*

Line 438-440, The experiment, and result are interesting! it seems to me that SSU1 depends on the expression dosage of FZF1. The dosage effect is also a hypothesis for single-gene overdominance phenomenon, that high or low expression may not be the optimal for phenotype. In this case, I wonder what if the native expression of FZF1 is reduced by RNAi or crispr. Did the authors test the level of native expression of FZF1 in the lines of SSU1/SSU1, SSU1/ssu1 and ssu1/ssu1? If the case is distinct to the (optimal) dosage hypothesis, it should be more amazing.

*Reply: As you said, the (optimal) dosage hypothesis is one important and may be real mechanism that explain the formation of heterosis. However, it usually explains the native heterosis. When the background is optimal to the homozygote (two alleles), it will be too high to the heterozygote (only one allele). Now, the heterozygote will present effect lower than half that present in homozygote, i.e. showing negative heterosis. Even so, our results also indicated that the native heterosis will decrease when the background increases (Figure S51 and line 572­582 of the revised manuscript).*

*In current research, the case of FZF1 and SSU1 does not show apparent phenomenon related to the (optimal) dosage hypothesis. Firstly, the SSU1 shows positive overdominnance when FZF1 is native. Secondly, our experiment indicated that the expression of SSU1 increased along with the upregulation of FZF1, as implied that the lower expression of SSU1 when FZF1 is native is not due to the overexpression of FZF1. In summary, the case that SSU1 shows heterosis when FZF1 is native is distinct to the (optimal) dosage hypothesis.*

*According to HoIIB model, the additive will transit into nonadditive when the background decreases. This can be implemented by the RNAi of background gene. But the reduction of FZF1 by RNAi is not suitable because the native expression of FZF1 has been insufficient.*

Line 449-450, The authors provided multiple evidence and simulations that proved the HoIIB phenomenon may exist for heterosis. But why it will happen? That is, why insufficient background (gene expression or phenotype) can make hybrid superior to parents. For example, why low FZF1 expression result in highest SSU1/ssu1 expression, the increase of FZF1 expression can increase SSU1/SSU1 expression faster than SSU1/ssu1? May the authors provide more explanations on how the HoIIB phenomenon happen biologically. It will be useful to broad readers.

*Reply: Sorry for that we did not clearly express the idea of the HoIIB model. The HoIIB model tells us that what we “observed” heterosis is not we have superior hybrids or heterozygote, but we have inferior parents or homozygote due to insufficient background of the relevant gene. It is different from the traditional idea on heterosis, in which one thinks that the hybrid (F1 or heterozygote for one gene) is superior to their parents (or homozygotes) or at least the mean of two parents (homozygotes) for mid-parent heterosis (here we consider only the positive heterosis), due to the “observed” phenomenon. If so, it implies that the use of hybrids or the heterozygote is the optimal option to maximize the function potential of the relevant gene. However, our HoIIB model will challenge the traditional idea on heterosis. HoIIB means Homo-Insufficiency under Insufficient Background. According to the model, the intrinsic mechanism of heterosis and dominance is that the insufficient background results in insufficient function of homozygote but relatively or even completely sufficient function of heterozygote in molecular performance, then the relevant gene or QTL shows dominance or even over-dominance in genetics, and finally the relevant combination shows heterosis in phenotype. That is, what we “observed” heterosis is not we have superior hybrids or heterozygote, but we have inferior parents or homozygote due to insufficient background of the relevant gene. If we make the background sufficient, the parent or homozygote will be improved and the heterosis will disappear. According to this model, the optimal option to maximize the function potential for a gene showing heterosis is to find its “insufficient” background that result in its heterosis and enhance the background so as to enhance the function of parents or homozygote of the gene.*

*Take FZF1 and SSU1 as an example to understand the above idea of HoIIB model. The natively low FZF1 expression provides an insufficient background to SSU1/SSU1, but the relatively or even completely sufficient background to SSU1/ssu1. Under such insufficient background (to SSU1/SSU1), homozygote SSU1/SSU1 can not express completely and even expresses lower than SSU1/ssu1 (the reason of even lower expression, we guess, is that two alleles in SSU1/SSU1 occupy more quantity of useless FZF1 that is needed to initiate the transcription of SSU1 than one allele in SSU1/ssu1), thus we see the phenomenon “highest SSU1/ssu1 expression under low FZF1 expression”. When the FZF1 expression was upregulated, SSU1/ssu1 appears low potential to be upregulated because its expression has (or almost) reached its maximum under low FZF1 expression, but SSU1/SSU1 has high potential to be upregulated because its expression is far to reach its maximum under low FZF1 expression.*

*For the explanation on how the HoIIB phenomenon happen biologically, please see the reply to comment related to Line 370-372.*

Review #2:

In the manuscript under review, the authors proposed a model (HoIIB) explaining the mechanism of additive and non-additive genetic effects. From the theorical point of view, the model can be viewed as a different version of the one in Omholt et al. 2000, which used the Hill equation to quantify the ligand-receptor binding. One of the three scenarios of the HoIIB model was experimentally validated in yeast by the authors. Furthermore, they claimed that the genetic basis of heterosis can also be explained by the HoIIB model. To provide evidence, field trials were conducted for 265 rice varieties and their single-cross hybrids with two testers. GWAS was performed based on re-sequenced genomic data.

I think the HoIIB model contains very nice idea and many results in this manuscript are interesting. However, I detected some major problems in the data analysis and the argumentation line of the HoIIB model, which were listed below:

1. The authors ignored the genetic expectations of the QTL effects being tested in their GWAS models, which could lead to incorrect interpretations of the results. As the authors explained, GWAS was performed based on the two subpopulations of parental lines (Indica and Japonica), and on the four subpopulations of hybrids (Indica/Japonica x 9311/Nipponbare) respectively. And in the latter case, the hybrid performance and mid-parent values were used as the response variable respectively (lines 172-176). Based on quantitative genetics theory, it is easy to see that in the GWAS with parental lines, the genetic expectation of the QTL effect (P_QTL) is the additive effect, in the GWAS with test-cross hybrids using the mid-parent heterosis as the response, the genetic expectation of the QTL effect (Hmp_QTL) is the dominance effect (assuming absence of epistasis), while in the GWAS with test-cross hybrids using the F1 performance as the response, the genetic expectation of the QTL effect (F1_QTL) is *d*−*a* or *d*+*a* (depending on whether the tester genotype is superior or inferior). Thus, the three sets of QTL (P_QTL, F1_QTL and Hmp_QTL) each has specific genetic effects. However, this fact was completely ignored in the comparison of results (lines 186-188). And because of this, some incorrect interpretations were made by the authors. Please see the next point.

*Reply: You put forward one very interesting topic in genetics and quantitative genetics, i.e. the genetic expectations of the QTL. As you said, it involves the same question to the next point. So we give the reply together below the next point.*

2. In the section “Non-additive, which is more variable than additive, is the main contributor to heterosis”, the authors focused on mid-parent heterosis (lines 197-199). But actually, this is a trivial statement as additive effects do not contribute to mid-parent heterosis at all, simply from the definition of additive and dominance effects. And this is true regardless whether epistasis is present or not. An easy two-locus example: *Gp*1=*a*1+*a*2+*aa*12, *Gp*2=−*a*1−*a*2+*aa*12, *GF*1=*d*1+*d*2+*dd*12, then *MPH*=*GF*1−12(*Gp*1+*Gp*2)=*d*1+*d*2+*dd*12−*aa*12. We can easily see that the additive effects cancelled out. From the authors’ argumentation line, I realized that they didn’t noticed the type of genetic effects represented by the three sets of QTL (P_QTL, F1_QTL and Hmp_QTL) are different. For example, the authors re-estimated the additive and dominance effects for all three sets of QTL and observed that F1_QTL and Hmp_QTL showed more non-additive pattern than P_QTL (lines 203-206). Indeed, this is expected because a P_QTL must have significant additive effects and an Hmp_QTL must have significant dominance effects. For an F1_QTL, the d−a or d+a effect is significant. Because both additive and dominance is involved, it is natural that it is more likely to show a non-additive pattern.

*Reply: Firstly, we completely agree with your opinion about the theory of quantitative genetics. In comparison of results including in lines 186-188 and other parts of the original manuscript, we did consider the traditional theory of quantitative genetics. However, we are glad to show an interesting explanation that combined our HoIIB model and the traditional theory of (quantitative) genetics and may be somewhat different from the traditional idea on the genetic expectations or genetic effect of the QTL.*

*Secondly, we are so sorry for the wrong statement of the subtitle (lines 197-199 of original manuscript). As you said, the QTL can cause heterosis of course if it is nonadditive as the definition of heterosis and nonadditive QTL in genetics, and we also pointed out this in lines 490-491 of the original manuscript and in lines 518-519 of the revised manuscript. In fact, the original expression was not what we would have aimed to express in the content under this subtitle. We meant that “Nonadditive QTLs, which are more variable than additive ones, are the main contributors to heterosis”.*

*Thirdly, we are glad to explain the above two replies as following:*

*In genetics and quantitative genetics, the so called additive, dominance, overdominance are the mathematical estimation according the phenotypic relationship among parents and their F1. So one QTL has both additive (a), dominant (d) or even overdominant (od) effects, which may or may not be detected depending on the investigated populations (parents, or cross combination including parents and F1) and the effect size of the QTL. It is curious for us whether there is difference in the additive effect and the (over-)dominant effect among QTLs detected in different populations, and if so, what the mechanisms are or what they imply. To investigate this, we classified them as additive, dominant, or overdominant preferred QTLs when |d/a|<0.5, 0.5≤|d/a|<1.5, or |d/a|≥1.5, respectively. Our results indicated that the majority of the F1_QTLs and Hmp_QTLs are overdominant-preferred (69.27% and 77.71%, respectively), with only a small portion of QTL represented additive effect (10.66% and 7.55%, respectively); and 44.16% P_QTLs are additive-preferred but more than 55% (37.08% dominant ones and 18.74% overdominant ones) are nonadditive-preferred. From the results, we can see that P_QTLs do not show the expected proportion of additive-preferred QTLs and Hmp_QTLs also contain a relative proportion of additive-preferred QTLs. No one explained why the proportion of additive-preferred P_QTLs is higher than F1_QTL and Hmp_QTL, and why the proportion of nonadditive-preferred F1_QTL and Hmp_QTL is higher than P_QTL.*

*But our HoIIB model can explain these results. According to HoIIB model, the larger dominant effect (d) was resulted from the insufficient function of homozygote under insufficient background, and thus this kind of QTL will show smaller additive effect (a); then the QTL will be easily detected among F1 or when using Hmp phenotype due to large d but difficult to be detected among parents due to small a. Similarly, the larger a represents one scenario where the background is sufficient enough to support the full function of homozygote, and thus this kind of QTL will show smaller d; then the QTL will be easily detected among parents due to large a but difficult to be detected among F1 or when using Hmp due to small d.*

*This also explained why dominant QTL is more variable than additive one. The background is insufficient when d is preferred, now the QTL effect will response easily to even the smaller change of background; and the background is sufficient when a is preferred especially when there is no dominant effect, now the QTL effect will be affected less by the smaller change of background.*

3. The HoIIB model provide an interesting explanation of the additive and non-additive pattern at the level of gene expression, but the bridge between non-additive gene expression and non-additive genetic effects on the phenotype (hence on heterosis) is missing. The theory of the HoIIB model presented in Additional File 3 follows the idea of the interpretation of additive, dominance and epistasis with gene regulatory networks (Omholt et al. 2000). The experimental validation in yeast verified Scenario 1, where one null-allele and one functional allele is presented at a polymorphic site (But the other two scenarios were not experimentally validated). In the validation, the ligand (input of the Hill equation, which is the core of the HoIIB model) is the expression level of transcription factor (FZF1), and the receptor (output of the Hill equation) is the expression level of the target gene (SSU1). Thus, the validation is convincing for the non-additive pattern at the level of gene expression. However, the authors proposed that the HoIIB model is able to explain the non-additive effects for the heterosis in phenotypes. This has not been experimentally validated yet. Of course, a direct validation would much more difficult than the level of gene expressions, as one first need to identify the potential ligand factors (the input of the Hill equation). Based on the results already obtained by the authors, a possible indirect way is to build up a link between the non-additive pattern in the gene expression and the non-additive effects on phenotypes. Unfortunately, this link is also missing in the current study. The authors did try to establish the link, but using a single-cross hybrid 9311 x PA64S (subsection “Non-additive expressed genes are highly dependent on the expression of their upstream transcription factors”) rather than using the same hybrid population for GWAS. This is in my opinion not convincing because both the genes which are differentially expressed and the QTL effects may vary in different bi-parental populations. Thus, the expression pattern of genes observed in this specific hybrid may not necessarily be the same as in the four test-cross hybrid populations. Ideally, the gene expressions should be taken from the same population whose phenotype was evaluated. For example, if one observes that the mid-parent heterosis at the level of gene expression is positively correlated with the mid-parent heterosis at the level of phenotype, then it is much more convincing.

*Reply: You and the other reviewer both put forward one critical question, that is, how to link gene expression with phenotype (heterosis). As our reply to the other reviewer’s comment (referred to Line 370-372 in the original manuscript and in lines of 384-386 in the revised version), we are indeed curious the molecular mechanisms of heterosis of observed phenotype such as yield. We also know that, for biologist or agronomist, the most interesting phenotype is such trait as plant height, biomass, and especially grain yield for rice. However, it really is difficult to show the example that can directly link gene expression with phenotype (heterosis); and you also noted “a direct validation would much more difficult than the level of gene expressions, as one first need to identify the potential ligand factors (the input of the Hill equation)”. As we said in the original manuscript (Line 370-372 in the original manuscript), the heterosis at the phenotype level are often resulted from the integrated effects of multi-factors at various intermediate and fundamental levels (such as different genes, QTLs, gene expression, and physiological traits), thus it is challenging to directly link the gene expression with heterosis of complex trait. In fact, our HoIIB model illustrates one molecular mechanism of the heterosis, which shows how the background of one gene shapes the relationship between potential function (the expected performance or phenotype when the background is sufficient) and observed function (observed performance or phenotype under different background) of the gene in parents and their F1. For simplicity, it’s better to choose the phenotype as simple as to investigate the relationship. The protein content is one kind of phenotype when we investigate the quality trait. Similarly, transcription level is also one kind of performance or phenotype when we investigate the transcription of one gene. As your comment noted, “the validation is convincing for the nonadditive pattern at the level of gene expression”, thus it also was experimentally validated that the HoIIB model is able to explain the nonadditive effects for the heterosis in phenotypes (here they are transcription levels). Of course, in the revised manuscript, we also experimentally validated the model using the maximum growth rate as phenotype. We observed the decrease of dominance or heterosis of the target gene (SSU1) along with the upregulation of its TF FZF1 (Figure S46 and line 455-468 of the revised manuscript).*

*We will compare Omholt’s results (Omholt et al. 2000) and our HoIIB model below next point. Here, we just say that the core ideas are distinctly different.*

*We agree that it will be ideal to select the samples for gene expression and the QTLs identification from the same population when trying to link nonadditive gene expression with the nonadditive genetic effect or heterosis. However, we do not try to link nonadditive gene expression pattern with nonadditive genetic effect or heterosis. Because the HoIIB model does not aim to bridge nonadditive gene expression and nonadditive genetic effects on the phenotype (hence on heterosis), and it aims to show how the background of one gene shapes the relationship between potential function and observed function of the gene in parents and their F1, and further results the heterosis phenomenon. The comparison between gene expression of cross 9311 x PA64S and GWAS QTLs aims to show the speculation that was inferred by HoIIB model. We speculated that the QTLs that were repeatedly identified as additive under many conditions (or backgrounds) represent the systematic limiting factors, which will be the insufficient background of most factors and thus usually show lower expression relative to most other factors. We really observed the decreased expression in repeatedly detected additive QTLs compared to the occasionally detected additive QTLs (Figure S52 and line 611-615 of the revised manuscript). Similarly, the QTLs that were repeatedly identified as dominance under many conditions (or backgrounds) represent the systematic limited factors, which will be the sufficient background of most factors and thus usually show higher expression relative to most other factors. We really observed the increased expression in repeatedly detected dominant QTLs compared to the occasionally detected ones (Figure S52 and line 611-615 of the revised manuscript).*

4. There are ambiguous interpretations on the “observed evidence” for the HoIIB model. For example, in the section “The HoIIB model was supported by different levels of evidence”, it was stated that “homo-insufficient expression was substantially observed in the higher parent for genes with dominant and overdominant transcription, compared to those with additive transcription” (lines 375-377). Here, unlike the experimental validation in yeast, the authors did not distinguish the transcript factors and the target genes. If I understood correctly, the additive or non-additive pattern and the homo-insufficient phenomenon was based on the expression of the same gene. Therefore, implicitly, the authors switched to the scenario in which the ligand and receptor are in a “dynamic balance” (meaning that in the Hill equation the input and output are NOT independent). This is different from the basic assumption of the HoIIB model “the ligand background concentration keeps constant among two homozygotes and the heterozygote” (Additional File 3, lines 66-67). Actually, the “dynamic balance” case has already been treated in Omholt et al. (2000). If this is what the authors meant to argue, then it is correct, but not novel and not new. The same ambiguous arguments occurred in lines 381-383, where the authors meant that the observed low phenotype of a parent served as an evidence of homo-insufficiency of the background of the non-additive pattern of the phenotype in the parent-hybrid combination.

*Reply: Yes, you are right, the additive or nonadditive pattern and the homo-insufficient phenomenon was based on the expression of the same gene. But, it does not describe the scenario of self-regulation (we guess you mean the self-regulation according to your comment), and our simulation and descriptions are based on sequential regulation model rather than the self-regulation one similar to the excellent work described in Omholt et al. (2000). The core idea of HoIIB model is that the gene with additive effect will show homo-insufficient phenomenon (lower down transcription at the transcription level) FOR THE SAME GENE and the gene with nonadditive effect will do not. And the background sufficiency of the considered gene will determine whether it shows nonadditive pattern and homo-insufficient phenomenon.*

*As for “dynamic balance”, we guess you mean the “equilibrium state”, i.e. the balance between association and disassociation of ligand and receptor in Hill equation. If so, we agree that the output was also measured at the “equilibrium state” in our simulation. But the ligand and the receptor are different molecules in our simulation, as being different from Omholt et al. (2000). We are sorry for that we gave a misleading description about the ligand background concentration in two homozygotes and the heterozygote (lines 66-67 of original Additional file 3). We would have meant that we assume that the input of ligand background concentration is consistent among two homozygotes and the heterozygote. Depending on the input of ligand background concentration, the receptor shows different genetic effect, including additive, partial dominance, dominance and overdominance. At last, the HoIIB model was deduced according to these simulated results. According to our HoIIB model, dominant and over-dominant mainly occurred when the background supply is relative insufficient, under this condition, the function of homozygous genotypes should not be completely realized; in comparison, additive mainly happened when the background supply is relatively sufficient, under this condition, the function of homozygous genotype should be nearly completely realized. Based on these speculations, HoIIB model predict that the homozygous performance of nonadditive factors may be smaller than that of additive factors in overall. In the real data, comparing the QTLs or expressed genes with different genetic effect types, we found that the above predictions could be observed at both expression and QTL level (Fig 4a and Fig 4b). For example, at the transcription level, homo-insufficient expression (as the performance) was substantially observed in the higher parent for genes with dominant and overdominant expressed genes, compared to those with additive expressed genes (Fig 4a). It needed to be pointed out that the above prediction are just according to the performance of the target factor, it did not need to investigate the performance of the upstream backgrounds. Therefore, we did not distinguish the transcript factors and the target genes; and TF vs its target gene is just one set of relationships between background and the target factors, which may include any directly or indirectly depended factors, such as TF vs its target gene, small RNA vs its target, signaling molecule vs its target, some gene vs the downstream gene of its pathway, and so on.*

To summarize, I think part of results in the manuscript is very interesting for researchers in the relevant area. The theory and experimental validation of the HoIIB model at the level of gene expression is convincing. The heterosis experimental data and GWAS results may also be publishable. But the whole story that the authors intended to tell, i.e., the HoIIB model explains the genetic basis of heterosis on phenotypic traits, is not convincing in several aspects. I am afraid I cannot recommend its publication in Genome Biology.

*Reply: Thank you for your review and comments. We hope our replies and revised version can meet your expectations and we also hope more communications if needed.*

**Second round of review**

**Reviewer 1**

In this study, the authors suggested a new model HoIIB explaining heterosis in rice. For easy understanding, the authors should briefly define the term Homo-insufficiency and background insufficiency. Any strategy to measure or evaluate them?

Two experiments were done in Changsha and Sanya, in which there are very different day length typical long day in Chang sha and short day in Shanya. Thus, the range of heading date should be very different between two enviroments. It is noted that heading date is the most important factor contributing to yield and yield heterosis. I suggest that the QTLs for heading date should be included or the flowering genes detected for other traits like SPP should be specifically mentioned, and they could be used to validate the model HoIIB by linking gene expression and yield/heterosis.

It is not clear the young panicle samples for the transcriptome profile was made in Changsha or Sanya environments or other independent conditions. The same condition as Changsha or Sanya is better because the hybrid PA64S/9311 was not included in the hybrid panel. Otherwise, the profiling results could not directly support the GWAS results done in both environments.

Differential expression in 1-mm, 2-mm and 3-mm panicles does not indicate the stability of genes. In fact, many genes are tissue specific, they stably expressed in the specific tissues in any genotypes. But they are not expressed in other tissues or the same tissues with differential developmental stages, and of course the environmental factor will probably promote or suppress their expression.

The HoIIB model was validated in Yeast. Of course, it is good evidence for the model. But the topic in this MS is focused on rice heterosis. There is a gap between gene expression and yield heterosis in rice. If the action model of some key heterosis genes like Ghd7 and Ghd8/RH8 fit the model both at gene expression level and phenotype level yield heterosis), the conclusion will be convincing in rice heterosis. I know it is very hard to quickly provide the evidence. At least, the authors should take some important heterosis genes as examples for discussing the possible link between phenotypic trait and gene expression.

Line 96: Additive has nothing to do with heterosis, but it is associated with hybrid performance.

Line 230: How to estimate dominance and overdominance of QTLs detected in the parental panel?

**Reviewer 2**

I would like to thank the authors for their detailed answers, which are very helpful for me to better understand their ideas in the manuscript. They did clarify an important issue due to misunderstandings, while I found that many statements were indeed misleading. Therefore, I think the manuscript still needs substantial revisions, especially the interpretation of the results from the rice heterosis data. Overall, I think this is a very interesting study. Below I inserted my reply in red color directly next to their answers. For convenience, I kept my original comments in bold font.

1. The authors ignored the genetic expectations of the QTL effects being tested in their GWAS models, which could lead to incorrect interpretations of the results. As the authors explained, GWAS was performed based on the two subpopulations of parental lines (Indica and Japonica), and on the four subpopulations of hybrids (Indica/Japonica x 9311/Nipponbare) respectively. And in the latter case, the hybrid performance and midparent values were used as the response variable respectively (lines 172-176). Based on quantitative genetics theory, it is easy to see that in the GWAS with parental lines, the genetic expectation of the QTL effect (P_QTL) is the additive effect, in the GWAS with test-cross hybrids using the mid-parent heterosis as the response, the genetic expectation of the QTL effect (Hmp_QTL) is the dominance effect (assuming absence of epistasis), while in the GWAS with test-cross hybrids using the F1 performance as the response, the genetic expectation of the QTL effect (F1_QTL) is d−a or d+a (depending on whether the tester genotype is superior or inferior). Thus, the three sets of QTL (P_QTL, F1_QTL and Hmp_QTL) each has specific genetic effects. However, this fact was completely ignored in the comparison of results (lines 186-188). And because of this, some incorrect interpretations were made by the authors. Please see the next point.

*Reply: You put forward one very interesting topic in genetics and quantitative genetics, i.e. the genetic expectations of the QTL. As you said, it involves the same question to the next point. So we give the reply together below the next point.*

2. In the section “Non-additive, which is more variable than additive, is the main contributor to heterosis”, the authors focused on mid-parent heterosis (lines 197-199). But actually, this is a trivial statement as additive effects do not contribute to mid-parent heterosis at all, simply from the definition of additive and dominance effects. And this is true regardless whether epistasis is present or not. An easy two-locus example: *Gp*1=*a*1+*a*2+*aa*12, *Gp*2=−*a*1−*a*2+*aa*12, *GF*1=*d*1+*d*2+*dd*12, then *MPH*=*GF*1−12(*Gp*1+*Gp*2)=*d*1+*d*2+*dd*12−*aa*12. We can easily see that the additive effects cancelled out. From the authors’ argumentation line, I realized that they didn’t noticed the type of genetic effects represented by the three sets of QTL (P_QTL, F1_QTL and Hmp_QTL) are different. For example, the authors re-estimated the additive and dominance effects for all three sets of QTL and observed that F1_QTL and Hmp_QTL showed more non-additive pattern than P_QTL (lines 203-206). Indeed, this is expected because a P_QTL must have significant additive effects and an Hmp_QTL must have significant dominance effects. For an F1_QTL, the *d*−*a* or *d*+*a* effect is significant. Because both additive and dominance is involved, it is natural that it is more likely to show a nonadditive pattern.

*Reply: Firstly, we completely agree with your opinion about the theory of quantitative genetics. In comparison of results including in lines 186-188 and other parts of the original manuscript, we did consider the traditional theory of quantitative genetics. However, we are glad to show an interesting explanation that combined our HoIIB model and the traditional theory of (quantitative) genetics and may be somewhat different from the traditional idea on the genetic expectations or genetic effect of the QTL.*

I have no objections in your attempt of using the HoIIB model or any other model/theory to explain the observed behavior of different types of QTL. But what I meant is that the genetic expectations of these QTL should not be ignored. At least I did not see any word about this point (neither in lines 186-188 or other parts in the original manuscript). More details are in my comments below.

*Secondly, we are so sorry for the wrong statement of the subtitle (lines 197-199 of original manuscript). As you said, the QTL can cause heterosis of course if it is nonadditive as the definition of heterosis and nonadditive QTL in genetics, and we also pointed out this in lines 490-491 of the original manuscript and in lines 518-519 of the revised manuscript. In fact, the original expression was not what we would have aimed to express in the content under this subtitle. We meant that “Nonadditive QTLs, which are more variable than additive ones, are the main contributors to heterosis”.*

I suppose you mean “Nonadditive-preferred QTL”? Otherwise there is no difference.

*Thirdly, we are glad to explain the above two replies as following: In genetics and quantitative genetics, the so called additive, dominance, overdominance are the mathematical estimation according the phenotypic relationship among parents and their F1. So one QTL has both additive (a), dominant (d) or even overdominant (od) effects, which may or may not be detected depending on the investigated populations (parents, or cross combination including parents and F1) and the effect size of the QTL. It is curious for us whether there is difference in the additive effect and the (over-)dominant effect among QTLs detected in different populations, and if so, what the mechanisms are or what they imply.*

Good point. But isn’t it natural to first have a look at the genetic expectations of the QTL effects in your GWAS model, because this is how you identified the QTL?

*To investigate this, we classified them as additive, dominant, or overdominant preferred QTLs when |d/a|<0.5, 0.5≤|d/a|<1.5, or |d/a|≥1.5, respectively. Our results indicated that the majority of the F1_QTLs and Hmp_QTLs are overdominant-preferred (69.27% and 77.71%, respectively), with only a small portion of QTL represented additive effect (10.66% and 7.55%, respectively); and 44.16% P_QTLs are additive-preferred but more than 55% (37.08% dominant ones and 18.74% overdominant ones) are nonadditive-preferred. From the results, we can see that P_QTLs do not show the expected proportion of additive-preferred QTLs and Hmp_QTLs also contain a relative proportion of additive-preferred QTLs.*

First of all, I think it is not surprising that the F1_QTL and Hmp_QTL also contain a certain proportion of additive-preferred QTL. Secondly, the proportion of additive-preferred QTL might indeed be a bit lower than expected. But we need to note two important factors: 1) The classification of additive-preferred and non-additive-preferred QTL was based on the re-estimated additive and dominance effects in the F1 population. The F1_QTL and Hmp_QTL were identified in the same population but the P_QTL were detected in a different population (namely the paretal lines). Therefore, we would expect a bit more inconsistency between the genetic expectation of QTL effects and the classification of additive-/non-additive-preferred QTL for the P_QTL than for the F1_QTL and Hmp_QTL. 2) The significance of additive/dominance effect in GWAS depends not only on the effect size but also the allele/genotype frequency, while in the classification of additive-/non-additive-preferred QTL, only the effect size played a role. Therefore, it is possible, for example, to have a marker with significant additive effect, non-significant dominance effect but classified as non-additive-preferred, or to have a marker with non-significant additive effect, significant dominance effect but classified as additive-preferred. Overall, I would think the classification of additive-/non-additive-preferred QTL is consistent, at least for a large part, with the genetic expectations of the QTL effects. The proportion of additive-preferred QTL in the P_QTL may warrant further explanations.

*No one explained why the proportion of additive preferred P_QTLs is higher than F1_QTL and Hmp_QTL, and why the proportion of nonadditive-preferred F1_QTL and Hmp_QTL is higher than P_QTL.*

Now you are talking about another type of comparison, namely comparing the proportion of additive-/non-additive-preferred QTL separately across P_QTL, F1_QTL and Hmp_QTL. And in fact, the results are perfectly consistent with the genetic expectations of the QTL effects. Namely, the proportion of additive-preferred P_QTL is higher than F1_QTL and Hmp_QTL and the proportion of non-additive-preferred F1_QTL and Hmp_QTL is higher than P_QTL because it is the additive effect makes a P_QTL significant while for an F1_QTL or Hmp_QTL to be significant, the dominance effect played a key role.

*But our HoIIB model can explain these results. According to HoIIB model, the larger dominant effect (d) was resulted from the insufficient function of homozygote under insufficient background, and thus this kind of QTL will show smaller additive effect (a); then the QTL will be easily detected among F1 or when using Hmp phenotype due to large d but difficult to be detected among parents due to small a.* *Similarly, the larger a represents one scenario where the background is sufficient enough to support the full function of homozygote, and thus this kind of QTL will show smaller d; then the QTL will be easily detected among parents due to large a but difficult to be detected among F1 or when using Hmp due to small d. This also explained why dominant QTL is more variable than additive one. The background is insufficient when d is preferred, now the QTL effect will response easily to even the smaller change of background; and the background is sufficient when a is preferred especially when there is no dominant effect, now the QTL effect will be affected less by the smaller change of background.*

As I said, further explanations on the behavior of different types of QTL are of course welcomed. But the insights from the genetic expectations of the QTL effects should be investigated at the first place.

3. The HoIIB model provide an interesting explanation of the additive and non-additive pattern at the level of gene expression, but the bridge between non-additive gene expression and non-additive genetic effects on the phenotype (hence on heterosis) is missing. The theory of the HoIIB model presented in Additional File 3 follows the idea of the interpretation of additive, dominance and epistasis with gene regulatory networks (Omholt et al. 2000). The experimental validation in yeast verified Scenario 1, where one null-allele and one functional allele is presented at a polymorphic site (But the other two scenarios were not experimentally validated). In the validation, the ligand (input of the Hill equation, which is the core of the HoIIB model) is the expression level of transcription factor (FZF1), and the receptor (output of the Hill equation) is the expression level of the target gene (SSU1). Thus, the validation is convincing for the non-additive pattern at the level of gene expression. However, the authors proposed that the HoIIB model is able to explain the nonadditive effects for the heterosis in phenotypes. This has not been experimentally validated yet. Of course, a direct validation would much more difficult than the level of gene expressions, as one first need to identify the potential ligand factors (the input of the Hill equation). Based on the results already obtained by the authors, a possible indirect way is to build up a link between the non-additive pattern in the gene expression and the nonadditive effects on phenotypes. Unfortunately, this link is also missing in the current study. The authors did try to establish the link, but using a single-cross hybrid 9311 x PA64S (subsection “Non-additive expressed genes are highly dependent on the expression of their upstream transcription factors”) rather than using the same hybrid population for GWAS. This is in my opinion not convincing because both the genes which are differentially expressed and the QTL effects may vary in different bi-parental populations. Thus, the expression pattern of genes observed in this specific hybrid may not necessarily be the same as in the four test-cross hybrid populations.

Ideally, the gene expressions should be taken from the same population whose phenotype was evaluated. For example, if one observes that the mid-parent heterosis at the level of gene expression is positively correlated with the mid-parent heterosis at the level of phenotype, then it is much more convincing.

*Reply: You and the other reviewer both put forward one critical question, that is, how to link gene expression with phenotype (heterosis). As our reply to the other reviewer’s comment (referred to Line 370-372 in the original manuscript and in lines of 384-386 in the revised version), we are indeed curious the molecular mechanisms of heterosis of observed phenotype such as yield. We also know that, for biologist or agronomist, the most interesting phenotype is such trait as plant height, biomass, and especially grain yield for rice. However, it really is difficult to show the example that can directly link gene expression with phenotype (heterosis); and you also noted “a direct validation would much more difficult than the level of gene expressions, as one first need to identify the potential ligand factors (the input of the Hill equation)”. As we said in the original manuscript (Line 370-372 in the original manuscript), the heterosis at the phenotype level are often resulted from the integrated effects of multi-factors at various intermediate and fundamental levels (such as different genes, QTLs, gene expression, and physiological traits), thus it is challenging to directly link the gene expression with* *heterosis of complex trait.*

Agreed.

*In fact, our HoIIB model illustrates one molecular mechanism of the heterosis, which shows how the background of one gene shapes the relationship between potential function (the expected performance or phenotype when the background is sufficient) and observed function (observed performance or phenotype under different background) of the gene in parents and their F1.*

The HollB model has two parts, namely “homo-insufficiency” and “insufficient background”. The former is about the output product (the receptor in the Hill equation) and the latter is about the input (the concentration of ligand). Therefore, if you intend to conclude that the HollB model is able to explain the “molecular mechanism of heterosis”, you would have to be precise on both the ligand and receptor. I realized I have misunderstood some points in the manuscript (see my reply to Point 4). More details are provided below.

*For simplicity, it’s better to choose the phenotype as simple as to investigate the relationship. The protein content is one kind of phenotype when we investigate the quality trait. Similarly, transcription level is also one kind of performance or phenotype when we investigate the transcription of one gene. As your comment noted, “the validation is convincing for the nonadditive pattern at the level of gene expression”, thus it also was experimentally validated that the HoIIB model is able to explain the nonadditive effects for the heterosis in phenotypes (here they are transcription levels). Of course, in the revised manuscript, we also experimentally validated the model using the maximum growth rate as phenotype. We observed the decrease of dominance or heterosis of the target gene (SSU1) along with the upregulation of its TF FZF1 (Figure S46 and line 455-468 of the revised manuscript).*

The added results on the experimental validation of HollB model for maximum growth rate is indeed very interesting and convincing. Here, the ligand and receptor are quite clear and the results well-matched your theory.

*We will compare Omholt’s results (Omholt et al. 2000) and our HoIIB model below next point. Here, we just say that the core ideas are distinctly different.*

*We agree that it will be ideal to select the samples for gene expression and the QTLs identification from the same population when trying to link nonadditive gene expression with the nonadditive genetic effect or heterosis. However, we do not try to link nonadditive gene expression pattern with nonadditive genetic effect or heterosis. Because the HoIIB model does not aim to bridge nonadditive gene expression and nonadditive genetic effects on the phenotype (hence on heterosis), and it aims to show how the background of one gene shapes the relationship between potential function and observed function of the gene in parents and their F1, and further results the heterosis phenomenon.*

Yes, I understood that the HollB model is not designed for bridging the non-additive gene expression and non-additive genetic effects on phenotype. But as you said, the aim is to show that the HollB model explain the molecular mechanism of heterosis. You either prove it directly, which is difficult for complex traits as we agreed, or provide an indirect proof through some intermediate step. As you have very convincing results that HollB model could explain the non-additive effects on the gene expression, if you could establish a link between non-additive gene expression and non-additive phenotype effects, then the argumentation line is completed. That is why I asked the question. Otherwise, you can only make the hypothesis that HollB model might be able to explain the heterosis of complex traits, given the results that you validated for gene expression and for a simpler trait (the maximum growth rate) in yeast, and given that there is some evidence of homo-insufficiency (but not the entire HollB model, please see my reply to Point 4) observed at the phenotype level.

*The comparison between gene expression of cross 9311 x PA64S and GWAS QTLs aims to show the speculation that was inferred by HoIIB model. We speculated that the QTLs that were repeatedly identified as additive under many conditions (or backgrounds) represent the systematic limiting factors, which will be the insufficient background of most factors and thus usually show lower expression relative to most other factors. We really observed the decreased expression in repeatedly detected additive QTLs compared to the occasionally detected additive QTLs (Figure S52 and line 611-615 of the revised manuscript). Similarly, the QTLs that were repeatedly identified as dominance under many conditions (or backgrounds) represent the systematic limited factors, which will be the sufficient background of most factors and thus usually show higher expression relative to most other factors. We really observed the increased expression in repeatedly detected dominant QTLs compared to the occasionally detected ones (Figure S52 and line 611-615 of the revised manuscript).*

I am a bit confused about your interpretation of the results in Figure S52. According to your HollB model, “homo-insufficiency” is observed when there is insufficient background factors and the gene shows dominance effects. But in three of four panels in Figure S52, the expression of candidate genes in the QTL classified as OD is much higher than those classified as additive. Where have I misunderstood?

4. There are ambiguous interpretations on the “observed evidence” for the HoIIB model. For example, in the section “The HoIIB model was supported by different levels of evidence”, it was stated that “homo-insufficient expression was substantially observed in the higher parent for genes with dominant and overdominant transcription, compared to those with additive transcription” (lines 375-377). Here, unlike the experimental validation in yeast, the authors did not distinguish the transcript factors and the target genes. If I understood correctly, the additive or non-additive pattern and the homo-insufficient phenomenon was based on the expression of the same gene. Therefore, implicitly, the authors switched to the scenario in which the ligand and receptor are in a “dynamic balance” (meaning that in the Hill equation the input and output are NOT independent). This is different from the basic assumption of the HoIIB model “the ligand background concentration keeps constant among two homozygotes and the heterozygote” (Additional File 3, lines 66-67). Actually, the “dynamic balance” case has already been treated in Omholt et al. (2000). If this is what the authors meant to argue, then it is correct, but not novel and not new. The same ambiguous arguments occurred in lines 381-383, where the authors meant that the observed low phenotype of a parent served as an evidence of homo insufficiency of the background of the non-additive pattern of the phenotype in the parent-hybrid combination.

*Reply: Yes, you are right, the additive or nonadditive pattern and the homo-insufficient phenomenon was based on the expression of the same gene. But, it does not describe the scenario of self-regulation (we guess you mean the self-regulation according to your comment), and our simulation and descriptions are based on sequential regulation model rather than the self-regulation one similar to the excellent work described in Omholt et al. (2000). The core idea of HoIIB model is that the gene with additive effect will show homo-insufficient phenomenon (lower down transcription at the transcription level) FOR THE SAME GENE and the gene with nonadditive effect will do not. And the background sufficiency of the considered gene will determine whether it shows nonadditive pattern and homo-insufficient phenomenon.*

After repeatedly reading your explanations here and the last paragraph below, I finally realized that you actually meant “homo-insufficiency” instead of “insufficient background” when you talked about the gene expression data for 9311, PA64S and their hybrid LYP9, the same happened when you talked about the observed phenotypes for different types of QTL identified in GWAS. As I have ready mentioned in the reply to Point 3, “homo-insufficiency” is just an observation about the output, while “HollB” describes the mechanism underlying the output so it includes both the output product and the ligand. In this sense, your experiments in yeast (both gene expression and the growth rate) indeed validated the entire HollB model, while in the gene expression data for 9311, PA64S and their hybrid LYP9, as well as the GWAS results for the yield-related traits in the rice hybrid populations, what you observed is “homo-insufficiency” instead of the entire “HollB” model. Of course, the observed “homo-insufficiency” suggests insufficient background according to your HollB model, but at this point you have no validations and no direct evidence for the “insufficient background”. Therefore, I am afraid that many descriptions, statements and claims in the manuscript are a bit misleading (for example, some statements sound like you have proved that the HollB model explains the mechanism of heterosis) and subject to revisions. Even the title is misleading because the rice data just provide some evidence of homo-insufficiency. Although the HollB model has been validated for two cases in yeast, for heterosis of rice yield it is still a hypothesized mechanism.

*As for “dynamic balance”, we guess you mean the “equilibrium state”, i.e. the balance between association and disassociation of ligand and receptor in Hill equation. If so, we agree that the output was also measured at the “equilibrium state” in our simulation. But the ligand and the receptor are different molecules in our simulation, as being different from Omholt et al. (2000).*

Yes, I understood that in your theory/simulation, the ligand and the receptor are independent factors and this makes your HollB model different from Omholt et al. (2000). As I mentioned above, what I misunderstood is your interpretation of the results.

*We are sorry for that we gave a misleading description about the ligand background concentration in two homozygotes and the heterozygote (lines 66-67 of original Additional file 3). We would have meant that we assume that the input of ligand background concentration is consistent among two homozygotes and the heterozygote. Depending on the input of ligand background concentration, the receptor shows different genetic effect, including additive, partial dominance, dominance and overdominance. At last, the HoIIB model was deduced according to these simulated results. According to our HoIIB model, dominant and over-dominant mainly occurred when the background supply is relative insufficient, under this condition, the function of homozygous genotypes should not be completely realized; in comparison, additive mainly happened when the background supply is relatively sufficient, under this condition, the function of homozygous genotype should be nearly completely realized. Based on these speculations, HoIIB model predict that the homozygous performance of nonadditive factors may be smaller than that of additive factors in overall. In the real data, comparing the QTLs or expressed genes with different genetic effect types, we found that the above predictions could be observed at both expression and QTL level (Fig 4a and Fig 4b). For example, at the transcription level, homo-insufficient expression (as the performance) was substantially observed in the higher parent for genes with dominant and overdominant expressed genes, compared to those with additive expressed genes (Fig 4a). It needed to be pointed out that the above predictions are just according to the performance of the target factor, it did not need to investigate the performance of the upstream backgrounds. Therefore, we did not distinguish the transcript factors and the target genes; and TF vs its target gene is just one set of relationships between background and the target factors, which may include any directly or indirectly depended factors, such as TF vs its target gene, small RNA vs its target, signaling molecule vs its target, some gene vs the downstream gene of its pathway, and so on.*

Thank you very much for the explanations. It helped to clarify an important issue due to misunderstandings.

**Authors Response**

**Point-by-point responses to the reviewers’ comments:**

Reviewer #1:

In this study, the authors suggested a new model HoIIB explaining heterosis in rice. For easy understanding, the authors should briefly define the term Homo-insufficiency and background insufficiency. Any strategy to measure or evaluate them?

*Reply：Thank you for your suggestions. We described the HoIIB in last graph of introduction (line 111 to 116 in the revised version) as: As indicated by HoIIB, it is the genetic background insufficient to maximize the function of two homo-alleles in parents, but relatively or even completely sufficient to maximize the function of one-allele in F1, thus resulting in the insufficient function of two homo-alleles in parents, but the relatively or completely sufficient function of one-allele in F1, that renders the target locus nonadditive in effect, as contributing to heterosis.*

*It is not easy to measure homo-insufficiency and background insufficiency directly. But several results have evaluated them indirectly. For homo-insufficiency, we observed the lower expression level in parent for genes with non-additive expression pattern than that for genes with additive expression pattern, the smaller effect in homozygous genotype of non-additive QTL than that of additive QTL, the lower performance in parent for trait showing non-additive than that showing additive. For background insufficiency, the yeast experiment has represented its effect. In the experiment, when the background (FZF1) of homozygous SSU1/SSU1 was relative insufficient (here, we use the expression of FZF1 to reflect the background supply level), gene SSU1 expressed as non-additive performance (Fig. 3e and Fig. 4e). On the contrary, when the background of homozygous was relative sufficient, gene SSU1 mainly expressed as additive performance (Fig. 3e and Fig. 4e).*

Two experiments were done in Changsha and Sanya, in which there are very different day length typical long day in Chang sha and short day in Shanya. Thus, the range of heading date should be very different between two enviroments. It is noted that heading date is the most important factor contributing to yield and yield heterosis. I suggest that the QTLs for heading date should be included or the flowering genes detected for other traits like SPP should be specifically mentioned, and they could be used to validate the model HoIIB by linking gene expression and yield/heterosis.

*Reply：Thank you for the very nice suggestions. We were aware that heading date is an important factor contributing to yield heterosis. As you mentioned, our experiments were done in Changsha (long day) and Sanya (short day) environments, the variation of heading date between the two environments was quite different, and this difference could be indirectly observed from the performance of spikelet number per panicle (SPP) and grain yield. In the revised version, we checked the heading date genes identified in SPP trait, and found that Ghd7 (Figure S10) and Hd3a (Figure S9) could be detected in SPP related traits. As for ghd7, the famous pre stop mutation site of Chr7_9154664 was a rare mutation in germplasm panel, its MAF was 0.0273, thus its association signal was not high and we could not effectively estimating its genetic effect; the other functional mutation of site Chr7_9155733 was located in the promoter regions, and with MAF as 0.295, yet the genetic effect of this site was mainly expressed as additive under both Changsha (short day) and Sanya (long day) environments, thus we did not put the results of Ghd7 in the manuscripts.*

*As for Hd3a, we observed that it mainly showed as additive effect under short day condition of Sanya, in both of our MCC panel and the assembled data of 1439 indica rice hybrids (Additional file1: Figure S52 d-e). However, under the long day condition of Changsha, Hd3a showed partial dominant effect, in which the function of Hd3a was not completely realized due to the insufficient backgrounds (Additional file1: Figure S52 f-g; line 610 to 634 in the revised version). It is apparently one case to validate the model HoIIB by linking gene expression and yield/heterosis. The more detailed description of Hd3a was conducted in Reply after next two questions.*

*In addition, we also tried to link gene expression and yield heterosis by comparing the expression of candidate genes in the detected QTLs, including additive and overdominant ones. In theory, the frequently detected additive factors may represent the systematic limiting factors (showing relative lower effect) that constrain the dominant or even overdominant factors from maximizing their functions; and in reverse, the frequently detected overdominant factors may represent the systematic limited factors (showing relative higher effect) that was constrained by the limiting factor. When comparing the frequently detected additive vs overdominant QTLs affecting yield traits, we observed the expected results that the candidate genes within the frequently detected additive QTLs displayed distinctly lower expression, compared to those within the frequently detected overdominant ones (Additional file1: Figure S53) (line 646 to 655 in the revised version).*

It is not clear the young panicle samples for the transcriptome profile was made in Changsha or Sanya environments or other independent conditions. The same condition as Changsha or Sanya is better because the hybrid PA64S/9311 was not included in the hybrid panel. Otherwise, the profiling results could not directly support the GWAS results done in both environments.

**Differential expression in 1-mm, 2-mm and 3-mm panicles does not indicate the stability of genes. In fact, many genes are tissue specific, they stably expressed in the specific tissues in any genotypes. But they are not expressed in other tissues or the same tissues with differential developmental stages, and of course the environmental factor will probably promote or suppress their expression.**

*Reply: Agree with your points in general. However, we do not focus on whether the gene expresses or not in different tissues, but we mainly focused on its different expression levels in different tissues. When the expression levels of gene A changed among tissues (as may be due to differential developmental stages or different environmental factor), the background of gene B that was regulated by A directly or indirectly also changed. So differential expression in 1- mm, 2-mm and 3-mm panicles may reflect certain aspect of the stability of one gene.*

The HoIIB model was validated in Yeast. Of course, it is good evidence for the model. But the topic in this MS is focused on rice heterosis. There is a gap between gene expression and yield heterosis in rice. If the action model of some key heterosis genes like Ghd7 and Ghd8/RH8 fit the model both at gene expression level and phenotype level yield heterosis), the conclusion will be convincing in rice heterosis. I know it is very hard to quickly provide the evidence. At least, the authors should take some important heterosis genes as examples for discussing the possible link between phenotypic trait and gene expression.

*Reply：Thank for your nice suggestions. We tried to make link between rice phenotypic traits and the HoIIB model as following. In order to find the possible link between rice yield gene and HoIIB model, we set three standards to screen rice gene: (1) the regulation relationship between the target gene and its upstream gene had been clarified in literature; (2) the upstream gene is strongly affected by environment condition, and we thus don’t have to take a long time to manipulate the background gene like the experiment conducted in yeast; (3) the genetic effect of the alleles had been verified and the gene expression level and phenotypic performance is linear correlated. We checked the heading date genes identified in SPP related QTLs and found that Hd3a basically meets the above mentioned three criteria. Firstly, the expression level of Hd3a shows significantly negative correlation with SPP, and its expression level highly depends on its background regulator Ehd1 (Additional file1: Figure S52 a-c). Secondly, it was reported that under the long day environment Ehd1 is severely repressed and under the short day environment Ehd1 will be less repressed and keeps a relatively high expression level. So, Hd3a will be in a relatively insufficient background under long day environment but sufficient background under short day environment, and we may expect that Hd3a will show nonadditive effect under long day environment but additive effect under short day environment. We investigated the SPP performance of indica×Nipponbare hybrids in Changsha (long day) and Sanya (short day) and 1439 hybrids in Hangzhou (long day) and Sanya (short day). The results showed that Hd3a appears nonadditive phenomenon under long day environment, where its background factor Ehd1 is seriously insufficient (Additional file1: Figure S52 d-e). And under short day environment, where the background regulator Ehd1 is relatively sufficient, Hd3a mainly expresses as additive (Additional file1: Figure S52 f-g). The phenotypic performance of Hd3a under long day and short day environments is basically consistent with the expectation of the HoIIB model. This content has been added as the section of “The performances of rice yield gene Hd3a can validate the HoIIB model” in DISCUSSION (line 610 to 634 in the revised version).*

*In addition, we also tried to link gene expression and yield heterosis by comparing the expression of candidate genes in the detected QTLs, including additive and overdominant ones. The detailed content has been described above (see also line 646 to 655 in the revised version).*

Line 96: Additive has nothing to do with heterosis, but it is associated with hybrid performance.

*Reply：Yes, we totally agree with you.*

Line 230: How to estimate dominance and overdominance of QTLs detected in the parental panel?

*Reply：The detail of estimating dominance and overdominance of QTLs detected in the parental panel are described in the section of Estimation of additive and dominance effects for each significant SNP locus and QTL in the Materials and methods parts. We estimated the genetic effect of each QTL based on the phenotypes of inbred parents and hybrids together, not just using inbred parents or hybrids.*

*In detail, we defined the tester’s genotype (Nipponbare or 9311) of each SNP as A, the nontester’s genotype as B. When some varieties in the MCC parental panel show as A and the others show as B, their F_1_ will show the genotypes A and H (the heterozygous genotype). For the investigated trait, we set P_A_ as the mean phenotype of parents with genotype A, P_B_ as that of parents with genotype B, F_A_ as that of F_1_ with genotype A and FH as that of F1 with genotype H. The additive effect of each SNP was a = |P_A_ – (P_A_ +P_B_)/2 |. The traditional estimation for dominance effect was expressed as d = F_H_ – (P_A_ +P_B_)/2, here we rescaled the dominance effects identified as d = F_H_ – (F_A_ – P_A_) – (P_A_ +P_B_)/2, in which F_A_ – P_A_ means the background heterozygous effects. For each QTL, the additive and dominant effects was estimated by the average additive and dominant effects of the significant SNPs within the QTL region.*

**Reviewer 2**

I would like to thank the authors for their detailed answers, which are very helpful for me to better understand their ideas in the manuscript. They did clarify an important issue due to misunderstandings, while I found that many statements were indeed misleading. Therefore, I think the manuscript still needs substantial revisions, especially the interpretation of the results from the rice heterosis data. Overall, I think this is a very interesting study. Below I inserted my reply in red color directly next to their answers. For convenience, I kept my original comments in bold font.

1. The authors ignored the genetic expectations of the QTL effects being tested in their GWAS models, which could lead to incorrect interpretations of the results. As the authors explained, GWAS was performed based on the two subpopulations of parental lines (Indica and Japonica), and on the four subpopulations of hybrids (Indica/Japonica x 9311/Nipponbare) respectively. And in the latter case, the hybrid performance and midparent values were used as the response variable respectively (lines 172-176). Based on quantitative genetics theory, it is easy to see that in the GWAS with parental lines, the genetic expectation of the QTL effect (P_QTL) is the additive effect, in the GWAS with test-cross hybrids using the mid-parent heterosis as the response, the genetic expectation of the QTL effect (Hmp_QTL) is the dominance effect (assuming absence of epistasis), while in the GWAS with test-cross hybrids using the F1 performance as the response, the genetic expectation of the QTL effect (F1_QTL) is d−a or d+a (depending on whether the tester genotype is superior or inferior). Thus, the three sets of QTL (P_QTL, F1_QTL and Hmp_QTL) each has specific genetic effects. However, this fact was completely ignored in the comparison of results (lines 186-188). And because of this, some incorrect interpretations were made by the authors. Please see the next point.

*Reply: You put forward one very interesting topic in genetics and quantitative genetics, i.e. the genetic expectations of the QTL. As you said, it involves the same question to the next point. So we give the reply together below the next point.*

*Reply: You put forward one very interesting topic in genetics and quantitative genetics, i.e. the genetic expectations of the QTL. As you said, it involves the same question to the next point. So we give the reply together below the next point.*

2. In the section “Non-additive, which is more variable than additive, is the main contributor to heterosis”, the authors focused on mid-parent heterosis (lines 197-199). But actually, this is a trivial statement as additive effects do not contribute to mid-parent heterosis at all, simply from the definition of additive and dominance effects. And this is true regardless whether epistasis is present or not. An easy two-locus example: *Gp*1=*a*1+*a*2+*aa*12, *Gp*2=−*a*1−*a*2+*aa*12, *GF*1=*d*1+*d*2+*dd*12, then *MPH*=*GF*1−12(*Gp*1+*Gp*2)=*d*1+*d*2+*dd*12−*aa*12. We can easily see that the additive effects cancelled out. From the authors’ argumentation line, I realized that they didn’t noticed the type of genetic effects represented by the three sets of QTL (P_QTL, F1_QTL and Hmp_QTL) are different. For example, the authors re-estimated the additive and dominance effects for all three sets of QTL and observed that F1_QTL and Hmp_QTL showed more non-additive pattern than P_QTL (lines 203-206). Indeed, this is expected because a P_QTL must have significant additive effects and an Hmp_QTL must have significant dominance effects. For an F1_QTL, the *d*−*a* or *d*+*a* effect is significant. Because both additive and dominance is involved, it is natural that it is more likely to show a nonadditive pattern.

*Reply: Firstly, we completely agree with your opinion about the theory of quantitative genetics. In comparison of results including in lines 186-188 and other parts of the original manuscript, we did consider the traditional theory of quantitative genetics. However, we are glad to show an interesting explanation that combined our HoIIB model and the traditional theory of (quantitative) genetics and may be somewhat different from the traditional idea on the genetic expectations or genetic effect of the QTL.*

I have no objections in your attempt of using the HoIIB model or any other model/theory to explain the observed behavior of different types of QTL. But what I meant is that the genetic expectations of these QTL should not be ignored. At least I did not see any word about this point (neither in lines 186-188 or other parts in the original manuscript). More details are in my comments below.

*Reply: We agree with your opinion, the genetic expectations of QTLs identified in parent, hybrid and the calculated middle parent heterosis (Hmp) should not be ignored before further analysis. In the current version of revised manuscripts, we added the description of different genetic expectations for the QTLs identified in inbred parents, hybrids and middle parent heterosis (line 214 to 222 in the revised version).*

*Secondly, we are so sorry for the wrong statement of the subtitle (lines 197-199 of original manuscript). As you said, the QTL can cause heterosis of course if it is nonadditive as the definition of heterosis and nonadditive QTL in genetics, and we also pointed out this in lines 490-491 of the original manuscript and in lines 518-519 of the revised manuscript. In fact, the original expression was not what we would have aimed to express in the content under this subtitle. We meant that “Nonadditive QTLs, which are more variable than additive ones, are the main contributors to heterosis”.*

I suppose you mean “Nonadditive-preferred QTL”? Otherwise there is no difference.

*Reply: Yes, you given a more accurate statement, we used the suggested term in the revised version.*

*Thirdly, we are glad to explain the above two replies as following: In genetics and quantitative genetics, the so called additive, dominance, overdominance are the mathematical estimation according the phenotypic relationship among parents and their F1. So one QTL has both additive (a), dominant (d) or even overdominant (od) effects, which may or may not be detected depending on the investigated populations (parents, or cross combination including parents and F1) and the effect size of the QTL. It is curious for us whether there is difference in the additive effect and the (over-)dominant effect among QTLs detected in different populations, and if so, what the mechanisms are or what they imply.*

Good point. But isn’t it natural to first have a look at the genetic expectations of the QTL effects in your GWAS model, because this is how you identified the QTL?

*Reply: Yes, as you suggested, we described the genetic expectations for the QTLs identified in inbred parents, hybrids and middle parent heterosis (line 214 to 222 in the revised version).*

*To investigate this, we classified them as additive, dominant, or overdominant preferred QTLs when |d/a|<0.5, 0.5≤|d/a|<1.5, or |d/a|≥1.5, respectively. Our results indicated that the majority of the F1_QTLs and Hmp_QTLs are overdominant-preferred (69.27% and 77.71%, respectively), with only a small portion of QTL represented additive effect (10.66% and 7.55%, respectively); and 44.16% P_QTLs are additive-preferred but more than 55% (37.08% dominant ones and 18.74% overdominant ones) are nonadditive-preferred. From the results, we can see that P_QTLs do not show the expected proportion of additive-preferred QTLs and Hmp_QTLs also contain a relative proportion of additive-preferred QTLs.*

First of all, I think it is not surprising that the F1_QTL and Hmp_QTL also contain a certain proportion of additive-preferred QTL. Secondly, the proportion of additive-preferred QTL might indeed be a bit lower than expected. But we need to note two important factors: 1) The classification of additive-preferred and non-additive-preferred QTL was based on the re-estimated additive and dominance effects in the F1 population. The F1_QTL and Hmp_QTL were identified in the same population but the P_QTL were detected in a different population (namely the paretal lines). Therefore, we would expect a bit more inconsistency between the genetic expectation of QTL effects and the classification of additive-/non-additive-preferred QTL for the P_QTL than for the F1_QTL and Hmp_QTL. 2) The significance of additive/dominance effect in GWAS depends not only on the effect size but also the allele/genotype frequency, while in the classification of additive-/non-additive-preferred QTL, only the effect size played a role. Therefore, it is possible, for example, to have a marker with significant additive effect, non-significant dominance effect but classified as non-additive-preferred, or to have a marker with non-significant additive effect, significant dominance effect but classified as additive-preferred. Overall, I would think the classification of additive-/non-additive-preferred QTL is consistent, at least for a large part, with the genetic expectations of the QTL effects. The proportion of additive-preferred QTL in the P_QTL may warrant further explanations.

*Reply: You put forward one key question related to the classification of additive-preferred and non-additive-preferred QTL. We completely agree with your opinion. Here, we would like to explain some aspects about the methods and results.*

*Firstly, the QTL identification and genetic effect estimation are different tasks, so we do not warry about the consistency between QTL identification and genetic effect estimation if we carry out them according to their respective theory and expectation. We really worry about the consistency between estimations of additive effect and dominant effect, because we must estimate them in different populations: the additive effect was estimated using the inbred parental population, but the dominant effect was done using the inbred parental population and F_1_ population together. To avoid the possible bias in estimating the additive effect and dominant effect, we used the modified formula that considered the genome background. In detail, we defined A as tester’s homozygous genotype (Nipponbare or 9311), B as non-tester’s homozygous genotype, H as heterozygous genotypes, P and F means the phenotypes of parent and F_1_ respectively. The additive effect was calculated as a = |P_A_ – (P_A_+P_B_)/2|. The dominant effect was calculated by the modified formula d = F_H_-(F_A_-P_A_) - (P_A_+P_B_)/2. Here, we used FAPA to estimate the genome background of dominant effect (or heterozygous effect) that was afforded by the other QTLs except for the calculated QTL in the F_1_ genome, in which FA is the phenotype of F1 with homozygous genotype A of the calculated QTL.*

*Secondly, it is really the truth that the effect type (additive or nonadditive) is determined by both the effect size and the allele/genotype frequency, and it is possible that a marker with significant additive effect, non-significant dominance effect but classified as non-additivepreferred, or vice versa. However, according to our current HoIIB model, the degree and value of dominance in real data is usually continuous depending on the background, it is difficult to make a rigid distinction between additive, dominant, and overdominant; in addition, as we all agreed, one QTL usually has both additive effect and dominant effect. Thus, we used a gradient division method same to Zhou et al. (2012) to classify the QTLs as additive-preferred, dominance-preferred and overdominance-preferred ones, rather than as additive, dominance and overdomiance ones. (Zhou, et al. Genetic composition of yield heterosis in an elite rice hybrid, Proceedings of the National Academy of Sciences of the United States of America, 2012, 109(39): 15847–15852).*

*We can not completely avoid the possible bias in classification of effect types and thus the biased proportion of additive-preferred QTL in the P_QTL. But, as analyzed theoretically above, we do not think the proportion of additive-preferred P_QTL is lower much than expectation.*

*No one explained why the proportion of additive preferred P_QTLs is higher than F1_QTL and Hmp_QTL, and why the proportion of nonadditive-preferred F1_QTL and Hmp_QTL is higher than P_QTL.*

Now you are talking about another type of comparison, namely comparing the proportion of additive-/non-additive-preferred QTL separately across P_QTL, F1_QTL and Hmp_QTL. And in fact, the results are perfectly consistent with the genetic expectations of the QTL effects. Namely, the proportion of additive-preferred P_QTL is higher than F1_QTL and Hmp_QTL and the proportion of non-additive-preferred F1_QTL and Hmp_QTL is higher than P_QTL because it is the additive effect makes a P_QTL significant while for an F1_QTL or Hmp_QTL to be significant, the dominance effect played a key role.

*Reply: Yes, we totally agree with you.*

*But our HoIIB model can explain these results. According to HoIIB model, the larger dominant effect (d) was resulted from the insufficient function of homozygote under insufficient background, and thus this kind of QTL will show smaller additive effect (a); then the QTL will be easily detected among F1 or when using Hmp phenotype due to large d but difficult to be detected among parents due to small a.* *Similarly, the larger a represents one scenario where the background is sufficient enough to support the full function of homozygote, and thus this kind of QTL will show smaller d; then the QTL will be easily detected among parents due to large a but difficult to be detected among F1 or when using Hmp due to small d. This also explained why dominant QTL is more variable than additive one. The background is insufficient when d is preferred, now the QTL effect will response easily to even the smaller change of background; and the background is sufficient when a is preferred especially when there is no dominant effect, now the QTL effect will be affected less by the smaller change of background.*

As I said, further explanations on the behavior of different types of QTL are of course welcomed. But the insights from the genetic expectations of the QTL effects should be investigated at the first place.

*Reply: Yes, we have described the genetic expectations of the QTL effect in the revised manuscript (line 214 to 222 in the revised version).*

3. The HoIIB model provide an interesting explanation of the additive and non-additive pattern at the level of gene expression, but the bridge between non-additive gene expression and non-additive genetic effects on the phenotype (hence on heterosis) is missing. The theory of the HoIIB model presented in Additional File 3 follows the idea of the interpretation of additive, dominance and epistasis with gene regulatory networks (Omholt et al. 2000). The experimental validation in yeast verified Scenario 1, where one null-allele and one functional allele is presented at a polymorphic site (But the other two scenarios were not experimentally validated). In the validation, the ligand (input of the Hill equation, which is the core of the HoIIB model) is the expression level of transcription factor (FZF1), and the receptor (output of the Hill equation) is the expression level of the target gene (SSU1). Thus, the validation is convincing for the non-additive pattern at the level of gene expression. However, the authors proposed that the HoIIB model is able to explain the nonadditive effects for the heterosis in phenotypes. This has not been experimentally validated yet. Of course, a direct validation would much more difficult than the level of gene expressions, as one first need to identify the potential ligand factors (the input of the Hill equation). Based on the results already obtained by the authors, a possible indirect way is to build up a link between the non-additive pattern in the gene expression and the nonadditive effects on phenotypes. Unfortunately, this link is also missing in the current study. The authors did try to establish the link, but using a single-cross hybrid 9311 x PA64S (subsection “Non-additive expressed genes are highly dependent on the expression of their upstream transcription factors”) rather than using the same hybrid population for GWAS. This is in my opinion not convincing because both the genes which are differentially expressed and the QTL effects may vary in different bi-parental populations. Thus, the expression pattern of genes observed in this specific hybrid may not necessarily be the same as in the four test-cross hybrid populations.

Ideally, the gene expressions should be taken from the same population whose phenotype was evaluated. For example, if one observes that the mid-parent heterosis at the level of gene expression is positively correlated with the mid-parent heterosis at the level of phenotype, then it is much more convincing.

*Reply: You and the other reviewer both put forward one critical question, that is, how to link gene expression with phenotype (heterosis). As our reply to the other reviewer’s comment (referred to Line 370-372 in the original manuscript and in lines of 384-386 in the revised version), we are indeed curious the molecular mechanisms of heterosis of observed phenotype such as yield. We also know that, for biologist or agronomist, the most interesting phenotype is such trait as plant height, biomass, and especially grain yield for rice. However, it really is difficult to show the example that can directly link gene expression with phenotype (heterosis); and you also noted “a direct validation would much more difficult than the level of gene expressions, as one first need to identify the potential ligand factors (the input of the Hill equation)”. As we said in the original manuscript (Line 370-372 in the original manuscript), the heterosis at the phenotype level are often resulted from the integrated effects of multi-factors at various intermediate and fundamental levels (such as different genes, QTLs, gene expression, and physiological traits), thus it is challenging to directly link the gene expression with* *heterosis of complex trait.*

Agreed.

*Reply: Thanks.*

*In fact, our HoIIB model illustrates one molecular mechanism of the heterosis, which shows how the background of one gene shapes the relationship between potential function (the expected performance or phenotype when the background is sufficient) and observed function (observed performance or phenotype under different background) of the gene in parents and their F1.*

The HollB model has two parts, namely “homo-insufficiency” and “insufficient background”. The former is about the output product (the receptor in the Hill equation) and the latter is about the input (the concentration of ligand). Therefore, if you intend to conclude that the HollB model is able to explain the “molecular mechanism of heterosis”, you would have to be precise on both the ligand and receptor. I realized I have misunderstood some points in the manuscript (see my reply to Point 4). More details are provided below.

*Reply: Thank you for your comments.*

*For simplicity, it’s better to choose the phenotype as simple as to investigate the relationship. The protein content is one kind of phenotype when we investigate the quality trait. Similarly, transcription level is also one kind of performance or phenotype when we investigate the transcription of one gene. As your comment noted, “the validation is convincing for the nonadditive pattern at the level of gene expression”, thus it also was experimentally validated that the HoIIB model is able to explain the nonadditive effects for the heterosis in phenotypes (here they are transcription levels). Of course, in the revised manuscript, we also experimentally validated the model using the maximum growth rate as phenotype. We observed the decrease of dominance or heterosis of the target gene (SSU1) along with the upregulation of its TF FZF1 (Figure S46 and line 455-468 of the revised manuscript).*

The added results on the experimental validation of HollB model for maximum growth rate is indeed very interesting and convincing. Here, the ligand and receptor are quite clear and the results well-matched your theory.

*Reply: Thank you for your nice comments.*

*We will compare Omholt’s results (Omholt et al. 2000) and our HoIIB model below next point. Here, we just say that the core ideas are distinctly different.*

*We agree that it will be ideal to select the samples for gene expression and the QTLs identification from the same population when trying to link nonadditive gene expression with the nonadditive genetic effect or heterosis. However, we do not try to link nonadditive gene expression pattern with nonadditive genetic effect or heterosis. Because the HoIIB model does not aim to bridge nonadditive gene expression and nonadditive genetic effects on the phenotype (hence on heterosis), and it aims to show how the background of one gene shapes the relationship between potential function and observed function of the gene in parents and their F1, and further results the heterosis phenomenon.*

Yes, I understood that the HollB model is not designed for bridging the non-additive gene expression and non-additive genetic effects on phenotype. But as you said, the aim is to show that the HollB model explain the molecular mechanism of heterosis. You either prove it directly, which is difficult for complex traits as we agreed, or provide an indirect proof through some intermediate step. As you have very convincing results that HollB model could explain the non-additive effects on the gene expression, if you could establish a link between non-additive gene expression and non-additive phenotype effects, then the argumentation line is completed. That is why I asked the question. Otherwise, you can only make the hypothesis that HollB model might be able to explain the heterosis of complex traits, given the results that you validated for gene expression and for a simpler trait (the maximum growth rate) in yeast, and given that there is some evidence of homo-insufficiency (but not the entire HollB model, please see my reply to Point 4) observed at the phenotype level.

*Reply: Yes, we totally agree with you.*

*We really tried to link gene expression and yield heterosis by comparing the expression of candidate genes in the detected QTLs, including additive and overdominant ones. In theory, the frequently detected additive factors may represent the systematic limiting factors (showing relative lower effect) that constrain the dominant or even overdominant factors from maximizing their functions; and in reverse, the frequently detected overdominant factors may represent the systematic limited factors (showing relative higher effect) that was constrained by the limiting factor. When comparing the frequently detected additive vs overdominant QTLs affecting yield traits, we observed the expected results that the candidate genes within the frequently detected additive QTLs displayed distinctly lower expression, compared to those within the frequently detected overdominant ones (Additional file1: Figure S53) (line 646 to 655 in the revised version).*

*In addition, we also tried to make link between rice phenotypic traits and the HoIIB model using Hd3a as an example (line 610 to 634 in the revised version, please see the fifth reply to reviewer 1 for detail).*

*The comparison between gene expression of cross 9311 x PA64S and GWAS QTLs aims to show the speculation that was inferred by HoIIB model. We speculated that the QTLs that were repeatedly identified as additive under many conditions (or backgrounds) represent the systematic limiting factors, which will be the insufficient background of most factors and thus usually show lower expression relative to most other factors. We really observed the decreased expression in repeatedly detected additive QTLs compared to the occasionally detected additive QTLs (Figure S52 and line 611-615 of the revised manuscript). Similarly, the QTLs that were repeatedly identified as dominance under many conditions (or backgrounds) represent the systematic limited factors, which will be the sufficient background of most factors and thus usually show higher expression relative to most other factors. We really observed the increased expression in repeatedly detected dominant QTLs compared to the occasionally detected ones (Figure S52 and line 611-615 of the revised manuscript).*

I am a bit confused about your interpretation of the results in Figure S52. According to your HollB model, “homo-insufficiency” is observed when there is insufficient background factors and the gene shows dominance effects. But in three of four panels in Figure S52, the expression of candidate genes in the QTL classified as OD is much higher than those classified as additive. Where have I misunderstood?

*Reply: We are so sorry that we didn’t describe it more clearly. We have modified the description in the revised manuscript as following.*

*In theory, the frequently detected additive factors may represent the systematic limiting factors that constrain the dominant or even overdominant factors from maximizing their functions, because the function (such as expression level) of the frequently detected additive factors is usually lower than those of the other factors in the regulation pathway; and in reverse, the frequently detected overdominant factors may represent the systematic limited factors (showing relative higher effect) that was constrained by the limiting factor, because the function (such as expression level) of the frequently detected overdominant factors is usually higher than those of the other factors in the regulation pathway. When comparing the frequently detected additive vs overdominant QTLs affecting yield traits, we observed the expected results that the candidate genes within the frequently detected additive QTLs displayed distinctly lower expression, compared to those within the frequently detected overdominant ones (Additional file1: Figure S53) (line 646-655 of the revised manuscript).*

4. There are ambiguous interpretations on the “observed evidence” for the HoIIB model. For example, in the section “The HoIIB model was supported by different levels of evidence”, it was stated that “homo-insufficient expression was substantially observed in the higher parent for genes with dominant and overdominant transcription, compared to those with additive transcription” (lines 375-377). Here, unlike the experimental validation in yeast, the authors did not distinguish the transcript factors and the target genes. If I understood correctly, the additive or non-additive pattern and the homo-insufficient phenomenon was based on the expression of the same gene. Therefore, implicitly, the authors switched to the scenario in which the ligand and receptor are in a “dynamic balance” (meaning that in the Hill equation the input and output are NOT independent). This is different from the basic assumption of the HoIIB model “the ligand background concentration keeps constant among two homozygotes and the heterozygote” (Additional File 3, lines 66-67). Actually, the “dynamic balance” case has already been treated in Omholt et al. (2000). If this is what the authors meant to argue, then it is correct, but not novel and not new. The same ambiguous arguments occurred in lines 381-383, where the authors meant that the observed low phenotype of a parent served as an evidence of homo insufficiency of the background of the non-additive pattern of the phenotype in the parent-hybrid combination.

*Reply: Yes, you are right, the additive or nonadditive pattern and the homo-insufficient phenomenon was based on the expression of the same gene. But, it does not describe the scenario of self-regulation (we guess you mean the self-regulation according to your comment), and our simulation and descriptions are based on sequential regulation model rather than the self-regulation one similar to the excellent work described in Omholt et al. (2000). The core idea of HoIIB model is that the gene with additive effect will show homo-insufficient phenomenon (lower down transcription at the transcription level) FOR THE SAME GENE and the gene with nonadditive effect will do not. And the background sufficiency of the considered gene will determine whether it shows nonadditive pattern and homo-insufficient phenomenon.*

After repeatedly reading your explanations here and the last paragraph below, I finally realized that you actually meant “homo-insufficiency” instead of “insufficient background” when you talked about the gene expression data for 9311, PA64S and their hybrid LYP9, the same happened when you talked about the observed phenotypes for different types of QTL identified in GWAS. As I have ready mentioned in the reply to Point 3, “homo-insufficiency” is just an observation about the output, while “HollB” describes the mechanism underlying the output so it includes both the output product and the ligand. In this sense, your experiments in yeast (both gene expression and the growth rate) indeed validated the entire HollB model, while in the gene expression data for 9311, PA64S and their hybrid LYP9, as well as the GWAS results for the yield-related traits in the rice hybrid populations, what you observed is “homo-insufficiency” instead of the entire “HollB” model. Of course, the observed “homo-insufficiency” suggests insufficient background according to your HollB model, but at this point you have no validations and no direct evidence for the “insufficient background”. Therefore, I am afraid that many descriptions, statements and claims in the manuscript are a bit misleading (for example, some statements sound like you have proved that the HollB model explains the mechanism of heterosis) and subject to revisions. Even the title is misleading because the rice data just provide some evidence of homo-insufficiency. Although the HollB model has been validated for two cases in yeast, for heterosis of rice yield it is still a hypothesized mechanism.

*Reply: We totally agree with your opinion. In last version, we did not provide strong direct evidence to validate the relationship between “insufficient background” and “homoinsufficiency”, but some indirect evidences. In the revised manuscript, we tried to make link between rice phenotypic traits and the HoIIB model using Hd3a as an example. Although we have described this above, we would like to attach it here again for your convenience.*

*In order to find the possible link between rice yield gene and HoIIB model, we set three standards to screen rice gene: (1) the regulation relationship between the target gene and its upstream gene had been clarified in literature; (2) the upstream gene is strongly affected by environment condition, and we thus don’t have to take a long time to manipulate the background gene like the experiment conducted in yeast; (3) the genetic effect of the alleles had been verified and the gene expression level and phenotypic performance is linear correlated. We checked the heading date genes identified in SPP related QTLs and found that Hd3a basically meets the above mentioned three criteria. Firstly, the expression level of Hd3a shows significantly negative correlation with SPP, and its expression level highly depends on its background regulator Ehd1 (Additional file1: Figure S52 a-c). Secondly, it was reported that under the long day environment Ehd1 is severely repressed and under the short day environment Ehd1 will be less repressed and keeps a relatively high expression level. So, Hd3a will be in a relatively insufficient background under long day environment but sufficient background under short day environment, and we may expect that Hd3a will show nonadditive effect under long day environment but additive effect under short day environment. We investigated the SPP performance of indica×Nipponbare hybrids in Changsha (long day) and Sanya (short day) and 1439 hybrids in Hangzhou (long day) and Sanya (short day). The results showed that Hd3a appears nonadditive phenomenon under long day environment, where its background factor Ehd1 is seriously insufficient (Additional file1: Figure S52 d-e). And under short day environment, where the background regulator Ehd1 is relatively sufficient, Hd3a mainly expresses as additive (Additional file1: Figure S52 f-g). The phenotypic performance of Hd3a under long day and short day environments is basically consistent with the expectation of the HoIIB model. This content has been added as the section of “T The performances of rice yield gene Hd3a can validate the HoIIB model” in DISCUSSION (line 610 to 634 in the revised version).*

*In addition, we also tried to link gene expression and yield heterosis by comparing the expression of candidate genes in the detected QTLs, including additive and overdominant ones. The detailed content has been described above (see also line 646 to 655 in the revised version).*

*Of course, we changed the manuscript title from “Large scale genomic and transcriptomic profiles of rice hybrids revealed a core mechanism underlying yield heterosis” to “Large scale genomic and transcriptomic profiles of rice hybrids revealed a core mechanism underlying heterosis” for more precise.*

*As for “dynamic balance”, we guess you mean the “equilibrium state”, i.e. the balance between association and disassociation of ligand and receptor in Hill equation. If so, we agree that the output was also measured at the “equilibrium state” in our simulation. But the ligand and the receptor are different molecules in our simulation, as being different from Omholt et al. (2000).*

Yes, I understood that in your theory/simulation, the ligand and the receptor are independent factors and this makes your HollB model different from Omholt et al. (2000). As I mentioned above, what I misunderstood is your interpretation of the results.

*Reply: Thanks for your comment.*

*We are sorry for that we gave a misleading description about the ligand background concentration in two homozygotes and the heterozygote (lines 66-67 of original Additional file 3). We would have meant that we assume that the input of ligand background concentration is consistent among two homozygotes and the heterozygote. Depending on the input of ligand background concentration, the receptor shows different genetic effect, including additive, partial dominance, dominance and overdominance. At last, the HoIIB model was deduced according to these simulated results. According to our HoIIB model, dominant and over-dominant mainly occurred when the background supply is relative insufficient, under this condition, the function of homozygous genotypes should not be completely realized; in comparison, additive mainly happened when the background supply is relatively sufficient, under this condition, the function of homozygous genotype should be nearly completely realized. Based on these speculations, HoIIB model predict that the homozygous performance of nonadditive factors may be smaller than that of additive factors in overall. In the real data, comparing the QTLs or expressed genes with different genetic effect types, we found that the above predictions could be observed at both expression and QTL level (Fig 4a and Fig 4b). For example, at the transcription level, homo-insufficient expression (as the performance) was substantially observed in the higher parent for genes with dominant and overdominant expressed genes, compared to those with additive expressed genes (Fig 4a). It needed to be pointed out that the above predictions are just according to the performance of the target factor, it did not need to investigate the performance of the upstream backgrounds. Therefore, we did not distinguish the transcript factors and the target genes; and TF vs its target gene is just one set of relationships between background and the target factors, which may include any directly or indirectly depended factors, such as TF vs its target gene, small RNA vs its target, signaling molecule vs its target, some gene vs the downstream gene of its pathway, and so on.*

Thank you very much for the explanations. It helped to clarify an important issue due to misunderstandings.

*Reply: Thank you for your nice comments.*

**Third round of review**

**Reviewer 1**

My questions were well addressed in this revision. I think it is acceptable for publication.

**Reviewer 2**

In the second revision, the quality of the manuscript has been further improved. Most of my points have been addressed in a satisfying way. The added results of rice Hd3a gene is quite convincing. I would like to thank the authors for carefully answering my comments. I just have a few more points below. The third one is about an important point in the Discussion section.

Line 212: "additive" -> "additive-preferred ones"

Line 216-218: "The above results indicated that one QTL can be detected in different panels, as implies that one QTL can take effect as additive or nonadditive in different panels." I think this sentence is not clear. I suggest "This means that QTL are detected based on different types of genetic effects in different panels".

Line 648-657: I think the description is still confusing. I don't understand the logic behind your arguments. On the other hand, I think the situation is very complicated. In your HoIIB model, one basic assumption is that the ligand background keeps constant across the three genotypes. Therefore, I suppose you meant the receptor instead of the background in HoIIB model when you talked about "additive factor" or "dominance factor". Then, according to your model, if you observed a dominant-preferred QTL, it means that it may have insufficient background. In contrast, if you observed an additive-preferred QTL, it should have sufficient background. But besides this, I think a very important point is the sign of the correlation (namely positive or negative) between the ligand and the receptor. This would determine which genotype has the best trait performance. Suppose that larger trait values are in favor and we focused on just one QTL, then if the correlation is positive, we should increase the ligand concentration. In this case additive is preferred and the trait value is maximized in one of the two homozygous genotypes (because sufficient background leads to additive). For example, the expression of SUU1 in yeast as you have showed in the manuscript. However, if the correlation is negative, we should decrease the ligand concentration. In this case dominance is preferred and the optimal genotype depends on the degree of dominance (if it is positive overdominance than the heterozygous genotype is the best, otherwise still one of the two homozygous genotypes). The Hd3a gene in rice provided an example as you added in the revised version. So, in my opinion, unless we have clearly identified the background, we cannot say in general whether the additive- or dominant-preferred QTL should be focused. Maybe I have misunderstood some points? But I would really appreciate if the authors could clarify.

**Authors Response**

**Point-by-point responses to the reviewers’ comments:**

Reviewer #1:

My questions were well addressed in this revision. I think it is acceptable for publication.

*Reply: Thanks for your time and nice comments.*

Reviewer #2:

In the second revision, the quality of the manuscript has been further improved. Most of my points have been addressed in a satisfying way. The added results of rice Hd3a gene is quite convincing. I would like to thank the authors for carefully answering my comments. I just have a few more points below. The third one is about an important point in the Discussion section.

*Reply: Thanks for your nice comments.*

Line 212: "additive" -> "additive-preferred ones"

*Reply: Very pertinent suggestion, and we have changed it in the revised version (line 212 in the revised versions).*

Line 216-218: "The above results indicated that one QTL can be detected in different panels, as implies that one QTL can take effect as additive or nonadditive in different panels." I think this sentence is not clear. I suggest "This means that QTL are detected based on different types of genetic effects in different panels".

*Reply: Yes, agree with you. In the revised version, we described it as “The above results indicated that one QTL can be detected based on different types of genetic effects (additive and nonadditive) in different types of panels by GWAS”.*

Line 648-657: I think the description is still confusing. I don't understand the logic behind your arguments. On the other hand, I think the situation is very complicated. In your HoIIB model, one basic assumption is that the ligand background keeps constant across the three genotypes. Therefore, I suppose you meant the receptor instead of the background in HoIIB model when you talked about "additive factor" or "dominance factor". Then, according to your model, if you observed a dominant-preferred QTL, it means that it may have insufficient background. In contrast, if you observed an additive-preferred QTL, it should have sufficient background. But besides this, I think a very important point is the sign of the correlation (namely positive or negative) between the ligand and the receptor. This would determine which genotype has the best trait performance. Suppose that larger trait values are in favor and we focused on just one QTL, then if the correlation is positive, we should increase the ligand concentration. In this case additive is preferred and the trait value is maximized in one of the two homozygous genotypes (because sufficient background leads to additive). For example, the expression of SUU1 in yeast as you have showed in the manuscript. However, if the correlation is negative, we should decrease the ligand concentration. In this case dominance is preferred and the optimal genotype depends on the degree of dominance (if it is positive overdominance than the heterozygous genotype is the best, otherwise still one of the two homozygous genotypes). The Hd3a gene in rice provided an example as you added in the revised version. So, in my opinion, unless we have clearly identified the background, we cannot say in general whether the additive- or dominant-preferred QTL should be focused. Maybe I have misunderstood some points? But I would really appreciate if the authors could clarify.

*Reply: Yes, the situation of heterosis and the HoIIB model are all very complicated. We are so sorry that we didn’t describe it more clearly in last version.*

*Firstly, we meant the investigated factor rather than only the receptor when we talked about “additive factor” or “nonadditive factor”. Of course, we really meant the receptor when we talked about “additive factor” or “nonadditive factor” in most cases of our manuscript, because we, for simplicity, took the receptor as the investigated factor and then its ligand is the background when constructing the model. However, we also can take the ligand as the investigated factor, and now its receptor will be the background. After all, the realistic situation is that the investigated factor and the background factor are just relative, and either of them can be served as the background of the other.*

*Secondly, we should distinguish the performance (the observed phenotype) and potential function (the potential phenotype) in understanding the HoIIB model. Homo-insufficiency means the insufficient phenotype of the investigated factor (observed phenotype less than potential phenotype), and “insufficient background” means that the potential function of the background is insufficient relative to the potential function of the investigated factor. In the context when we compared the repeatedly detected additive-preferred or overdominant-preferred QTLs, we compared the potential function of the investigated factor rather than the background and it does not have to mean the receptor.*

*Thirdly, let us understand the logic that the repeatedly detected additive factor has lower expression level than the repeatedly detected overdominant factor as following. Let us suppose a situation: one receptor and its ligand are independent to all other factors. If the ligand is insufficient relative to the receptor, the receptor will be sufficient relative to the ligand, and this means that the ligand has lower potential function relative to the receptor. Now, the receptor will be nonadditive and the ligand will be additive. That is, the potential function of the additive factor is lower than that of the nonadditive factor. However, in the real organism, one receptor may have more than one ligands and one ligand may also can bind to more than one receptors. It depends on the genetic background, or environment, or developmental stages whether some one of the receptors encounters some one of the ligands. Let us suppose two situations for one receptor with more than one ligands. Situation 1, if one receptor always is additive no matter the ligands, we think that all ligands are sufficient relative to the receptor and the receptor is insufficient relative to all its ligands, and it also means that the potential function of the receptor is lower than those of all its backgrounds. Situation 2, if one receptor is additive under some ligands but nonadditive under other ligands, we think that some ligands are sufficient relative to the receptor and the receptor is insufficient relative to these ligands, but other ligands are insufficient relative to the receptor and the receptor is sufficient relative to those ligands. This situation also means that the potential function of the receptor is lower than these of some backgrounds but higher than those of other backgrounds. In statistics, we expect that the potential of the receptor in situation 1 is lower than that in situation 2. If the expression level can represent the potential function of one gene to some extent, we expect that the receptor that repeatedly appears additive under different ligands or backgrounds (situation 1) will express lower than the one that appears additive under a few ligands or backgrounds (situation 2). In the same way, we expect that the expression level of the receptor will get higher when the times that it was detected as nonadditive get more. According to the above logic, when we compared the expression level of the candidate genes within an additive-preferred QTL or an overdominant-preferred QTL, we compared the potential function of the investigated factor rather than the background, and we expect that the repeatedly detected additive-preferred QTL will show lower expression level and would represent one systematic limiting factor usually (Figure S54). This is true in spite of positive or negative regulations because the HoIIB model is true in both positive and negative regulations as showed in Figure S27, S30 and S34-35.*

*Of course, you put forward one very important point, i.e. positive or negative correlation between the ligand and the receptor. Agree with you, we should upregulate one positive factor and downregulate the negative factor to improve the trait. For the positive regulation, when we upregulate the systematic limiting factor, we can release a batch of factors that were limited. For the negative regulation, it may be more efficient to downregulate the systematic negative factor than the non-systematic negative factor (the non-systematic factor implies higher potential function that can not be easily downregulated or more alternative factors that can compensate each other). Thus, the frequently detected additive factors, rather than the overdominant factors, should be focused in future breeding programs. Of course, we understand that further studies are needed to validate the claims related to the currently observed phenomena and results.*

*We hope the above explanation has answered your question and we described the above logic simply in the revised manuscript in Line 644-650, 663-668 and 673-674.*

**Fourth round of review**

**Reviewer 2**

The authors have addressed all my comments and suggestions in the revised manuscript. I have no further points.
